# Research progress on non-coding RNA regulatory networks and targeted therapy in diabetic nephropathy

**DOI:** 10.3389/fendo.2025.1625307

**Published:** 2025-07-29

**Authors:** Xiaxia Wang, Ruge Jing, Tong Yang, Ruiwen Shao, Fan Yang, Yangyang Shi, Xiujuan Yang, Dong An, Yonglin Liang

**Affiliations:** ^1^ School of Basic Medicine, Gansu University of Chinese Medicine, Lanzhou, Gansu, China; ^2^ Teaching Experiment Training Center, Gansu University of Chinese Medicine, Lanzhou, Gansu, China; ^3^ School of Pharmacy, Gansu University of Chinese Medicine, Lanzhou, Gansu, China

**Keywords:** diabetic nephropathy, non-coding RNAs, ceRNA networks, podocyte, mesangial cells, renal tubular epithelial cells, cell death, fibrosis

## Abstract

Diabetic Nephropathy (DN), a leading cause of disability and mortality in patients with diabetes, has become a complex global clinical issue that poses a severe challenge to public health. Research indicates that Non-coding RNAs (ncRNAs) participate in cell death and fibrosis through an endogenous competitive RNA (ceRNA) network. This network regulates kidney-specific cells such as podocytes, mesangial cells, and renal tubular epithelial cells, thereby establishing a multifaceted regulatory mechanism in DN progression. Furthermore, exosomal ncRNAs and their ceRNA networks, stem cell-derived exosomal ncRNAs, related biomolecules, and the targeted regulation of ncRNAs and ceRNA networks by traditional Chinese medicine all play significant roles in the advancement of DN. This review systematically summarizes the content of ncRNAs, ceRNA networks and DN, exosome ncRNA intervention in DN progression, and targeted regulation of ncRNA intervention in DN progression. Concurrently, it discusses the research progress and therapeutic status of ncRNAs as clinical biomarkers, challenges facing ncRNA-targeted therapy, therapeutic efficacy of exosomal ncRNAs and stem cell-derived exosomal ncRNAs, pharmacokinetic limitations of Chinese medicine components in regulating DN progression through ncRNA intervention, and analyses the bottlenecks in ncRNA-based diagnosis and cross-species conservation of circRNAs/lncRNAs. This study aimed to provide new insights for the in-depth exploration of the molecular mechanisms underlying DN and the development of targeted therapeutic strategies.

## 1 Introduction

As a complex endocrine condition, diabetes mellitus (DM) manifests through impaired glucose homeostasis and sustained hyperglycemia (HG), leading to long-term damage, functional impairment and multi-organ failure, particularly the kidneys (1). Epidemiological projections indicate that the worldwide prevalence of diabetes mellitus is anticipated to exceed 780 million cases by the year 2024, with DM emerging as a serious global health challenge that imposes substantial socioeconomic burdens on individuals, families, and societies (2). DN, a grave microvascular complications of DM affecting 20–50% of DM patients, stands as a predominant contributor to end-stage renal disease (3). The pathological sign of DN are characterized by extracellular matrix (ECM) protein pile up, basement membrane thickening, renal tubulointerstitial fibrosis, and epithelial–mesenchymal transition (EMT) of renal tubular epithelium, with inflammation, oxidative stress (OS), apoptosis, and pyroptosis act as key mediators in the pathophysiological progression of DN (4–6). Substantial evidence indicates that despite continuous updates in DN prevention and therapeutic approaches, its incidence rates persist in escalating, underscoring the incomplete elucidation of DN pathogenesis. Thus, unravelling the underlying mechanisms driving DN advancement and formulating novel intervention strategies hold paramount clinical significance (7, 8). Mounting evidence demonstrates that dysfunction of podocytes, glomerular mesangial cells (GMCs), and renal tubular epithelial cells (TECs) critically influences DN progression by modulating its pathological hallmarks (9–11). Consequently, the development of therapeutic approaches prioritizing either the prevention of podocyte injury, GMCs proliferation, and TECs damage, or the facilitation of functional recovery in these cellular compartments, represents a promising strategic avenue for DN management.

ncRNAs, once considered transcriptional ‘noise’ due to their lack of protein-coding capacity, have garnered substantial scientific attention across various disease pathologies with advancements in transcriptomic sequencing and genomic research (12). Comprising primarily miRNAs, lncRNAs, and circRNAs, ncRNAs regulate a wide range of biological processes at post-transcriptional and epigenetic levels, demonstrating marked relevance to DN progression (13, 14). Critically, emerging studies reveal that lncRNAs and circRNAs function as miRNA sponges through ceRNA mechanisms, thereby modulating pathological processes—inflammation, OS, apoptosis, and pyroptosis. This regulatory axis exerts targeted effects on podocytes, GMCs, and TECs, thereby driving DN pathogenesis (15–20). Consequently, systematic elucidation of ceRNA network dynamics within these renal cellular compartments not only advances our understanding of cell-type-dependent pathogenic mechanisms in DN but also provides new perspectives for developing targeted therapeutic strategies.

Emerging evidence highlights a novel exosome-mediated intercellular communication mechanism that has recently gained significant interest (21). Exosomes—small bilayer lipid membrane vesicles measuring 40–100 nm in diameter—are recognized as innovative biomarkers containing complex cargoes of RNAs and proteins (22). Exosomes, secreted through exocytotic processes into the interstitial compartment and absorbed by recipient cells, act as critical mediators in various disease states through their role in intercellular communication and molecular cargo delivery (23). Notably, although exosomes are now acknowledged as critical carriers of circRNAs and as potential therapeutic targets for fibrosis mitigation, their precise mechanistic roles in DN progression remain unclear (24). Consequently, further elucidation of exosomal mechanisms mediates the disease progression of DN holds considerable promise for advancing molecularly targeted therapeutic strategies against this debilitating condition.

To date, numerous therapeutic strategies targeting pathogenic factors in DN, including glycemic control, administration of angiotensin-converting enzyme inhibitors, and blockade of the RAAS, have been implemented and are partially effective in slowing DN progression. However, the complexity of its pathogenesis, coupled with the predominantly single-target focus of current medical approaches, imposes significant limitations, importantly, these strategies are incapable of reversing established structural renal damage (25, 26). As a result, novel multitarget methodologies are gaining increasing scientific attention. Notably, traditional Chinese medicine (TCM) has been extensively used in China for managing diverse pathologies, including DM, with demonstrated clinical efficacy (27, 28). Moreover, TCM’s inherent advantages of multi-purpose, multi-level, multi-stage therapeutic modulation have propelled growing research focus on its potential role in DN intervention (29). Recent studies suggest that TCM may exert protective effects against DN progression through the regulation of ncRNA or ceRNA networks, thereby representing a promising complementary therapeutic approach (30, 31).

This review provides a systematic summary of ncRNAs, ceRNA networks and DN, as well as the intervention of exosomal ncRNAs in DN progression and the targeted regulation of ncRNAs in DN progression. This will establish the key role of ncRNAs in DN, with the aim of providing new insights into the mechanisms of DN and targeted treatment.

## 2 ncRNA

### 2.1 lncRNA/circRNA/miRNA

LncRNAs represent a distinct category of RNA molecules exceeding 200 nucleotides, characterized by minimal or absent protein-coding capacity (32). Once considered biologically insignificant, advancements in genomic research have revealed that lncRNAs possess diverse regulatory functions, predominantly modulating gene expression through mechanisms such as alternative splicing, translational control, epigenetic regulation, and chromatin remodeling, these processes critically influence cellular proliferation, apoptosis, chromatin dynamics, and cell cycle progression, thereby contributing to both physiological and pathological processes (33, 34). Notably, emerging evidence highlights lncRNAs’ roles not only in embryonic development, tissue differentiation, and organogenesis but also in the pathogenesis of cardiovascular and metabolic disorders (35, 36). For instance, comprehensive lncRNA profiling has identified aberrantly expressed lncRNAs in DN, which are implicated as risk factors in disease progression, these transcripts accelerate renal interstitial fibrosis, promote cellular proliferation and EMT, and intersect with key DN-associated pathways, including mitochondrial bioenergetics, endoplasmic reticulum stress, inflammation, fibrosis, and apoptosis (15, 37, 38). Targeted modulation of lncRNAs thus emerges as a viable therapeutic avenue for DN treatment and prophylaxis.

Distinct from their lncRNAs, microRNAs constitute a class of compact, intrinsically derived non-protein-coding transcripts (spanning 19–22 nucleotide residues) that exert precise transcriptional modulation through post-transcriptional control mechanisms (39). miRNAs mediate sequence-specific gene silencing through complementary annealing to 3’ UTRs of cognate mRNAs, touching off either translational blockade or transcript destabilization via RISC-mediated mechanisms (40). Beyond this canonical role, miRNAs orchestrate critical biological processes including metabolism, cellular differentiation, proliferation, and apoptosis, with dysregulation implicated in cancer and metabolic disorders (41, 42). Notably, studies have established correlations between podocyte injury in DN and aberrant miRNA expression in both circulating and renal tissues, with experimental modulation of specific miRNAs demonstrating protective effects against podocyte damage in DN models (43). Furthermore, miRNAs are mechanistically involved in DN-associated inflammation, OS, apoptosis, aberrant cell proliferation, and renal fibrosis. Emerging evidence highlights miRNA-mediated crosstalk influencing cellular plasticity in the kidney, encompassing epithelial-myofibroblastic transdifferentiation, endothelial-fibrogenic conversion, and macrophage phenotypic reprogramming—central mechanisms underpinning renal fibrogenesis in DN pathogenesis (44–46). Collectively, these findings emphasize the central regulatory roles of miRNAs in both cell-specific pathophysiological mechanisms and molecular cascades underlying DN.

CircRNAs, a more recently identified class of endogenous ncRNAs, are mechanistically distinct from other ncRNAs such as lncRNAs. Their biogenesis primarily involves back-splicing, wherein canonical splice donor-acceptor pairing is subverted through non-linear ligation, establishing phosphodiester bonds between downstream 5’ donors and upstream 3’ acceptors in retrograde orientation across exonic domains, thereby generating covalently circularized RNA species, this circular architecture confers exceptional stability by rendering circRNAs resistant to exonuclease degradation, underpinning their potent regulatory capacity in gene expression modulation (47, 48). The latest evidence suggests that circRNAs play pivotal regulatory roles in the pathogenesis of diverse diseases, including cardiovascular disorders, neurological conditions, and malignancies (49–51). Consequently, their pathologically distinct molecular signatures are progressively validated as dual-utility modalities, functioning both as clinical detection indices and precision-targeted therapeutic nodes. Recent studies further show that DM perturbs circulatory circRNA expression patterns, with dysregulated circRNAs directly implicated in DN progression (52). Additionally, cumulative circRNA microarray analyses have revealed extensive circRNA dysregulation in DN, where specific circRNAs functionally modulate GMCs activity and mediate pathological cascades in DN (53). Nevertheless, circRNA research in DN remains in its infancy, with fundamental questions regarding their biosynthesis, regulatory networks, and mechanistic contributions to renal pathophysiology largely unresolved. Systematic elucidation of circRNA-mediated molecular mechanisms in DN thus represents a critical avenue for developing novel therapeutic strategies against this condition.

### 2.2 miRNA ‘sponge’ effect

The ceRNA mechanism represents a novel regulatory model of gene expression. In contrast to miRNA networks that exert negative regulation, ceRNA-mediated regulation exhibits greater refinement and complexity, encompassing a broader spectrum of RNA species including mRNA, lncRNA, circRNA, and miRNA itself (54, 55). Consequently, this mechanism has attracted considerable scientific interest in recent years.

**Figure 1:**
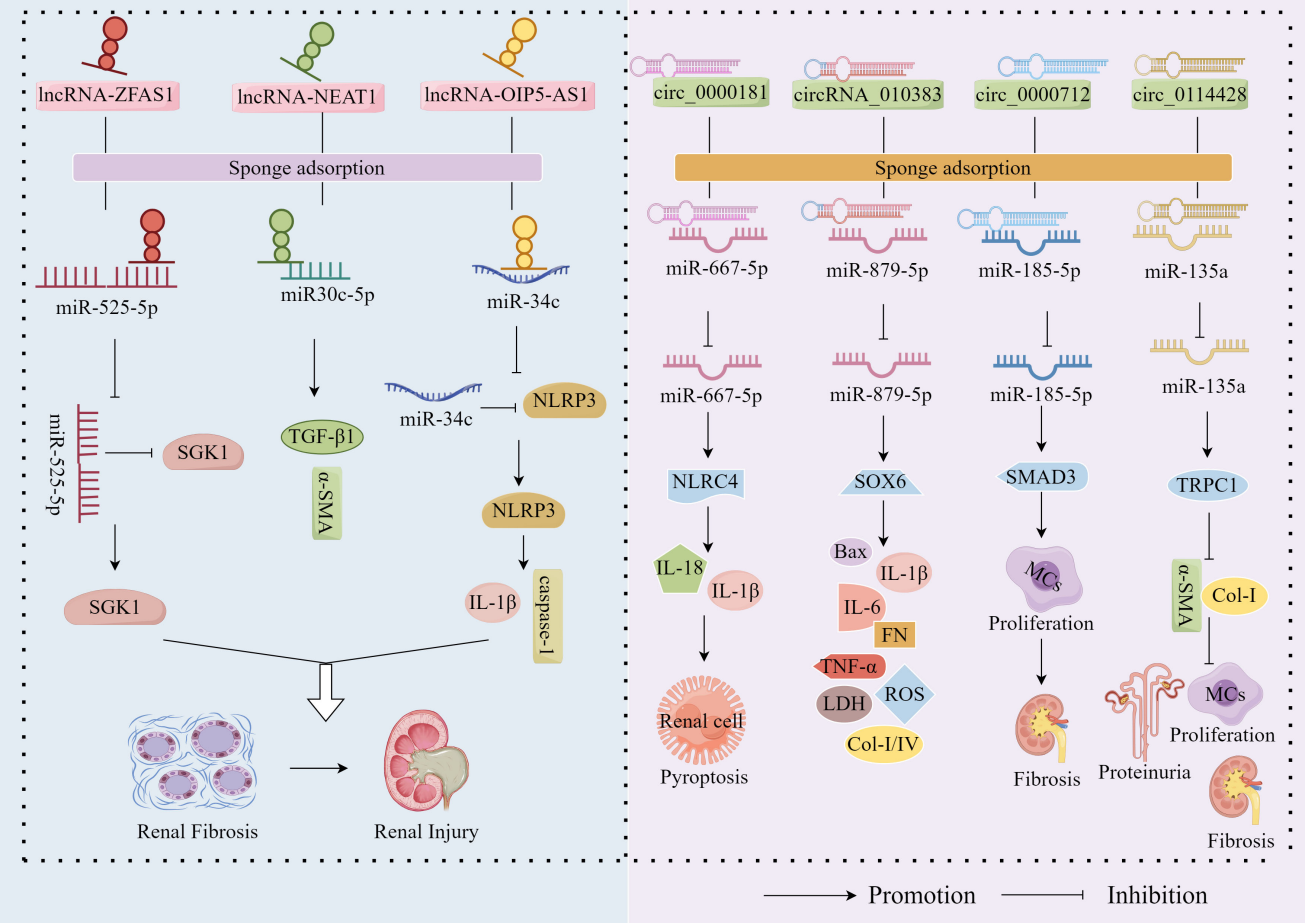
miRNA ‘sponge’ effect. LncRNA-ZFAS1, lncRNA-OIP5-AS1 and lncRNA-NEAT1 exacerbated the fibrotic progression of DN by sponge adsorption of miR-525-5p, miR30c-5p and miR-34c, respectively, which deregulated the inhibition of SGK1 by miR-525-5p and the inhibition of NLRP3 by miR-34c. In addition, circ_0000181, circ_0000712 and circ_0114428 up-regulated the expression of NLRC4, SOX6 and SMAD3 by sponge adsorption of miR-667-5p, miR-879-5p and miR-185-5p, respectively, which promoted inflammation, apoptosis, OS and fibrosis and accelerated DN progression; however, circRNA_010383 inhibited DN progression by sponge adsorption of miR-135a, which deregulated the inhibition of TRPC1 by miR-135a.

The ceRNA hypothesis provides a foundational framework for understanding miRNA function, proposing that RNA transcripts can bind to complementary miRNA sequences and regulate target mRNA expression through competitive occupation of shared miRNA binding sites (56). Recent investigations have unveiled intricate reciprocal regulation between miRNAs and lncRNAs, with emerging evidence demonstrating lncRNAs’ capacity to function as miRNA sponges. Through ceRNA-mediated interactions, these lncRNAs sequester miRNAs, thereby allowing target mRNAs to escape miRNA-mediated repression (57). Contemporary research has further established the critical involvement of the lncRNA-miRNA-mRNA regulatory axis in DN pathogenesis (58) (**Figure 1**). Exemplifying this mechanism, lncRNA-ZFAS1 acts as a molecular sponge for miR-525-5p, effectively neutralizing its suppressive effect on SGK1. This interaction culminates in SGK1 overexpression that drives fibrosis and inflammasome activation, thereby exacerbating DN progression (59). Similarly, lncRNA-OIP5-AS1 facilitates EMT in HG-stimulated HK2 cells by downregulating miR-30c-5p expression, which consequently upregulates fibrogenic mediators TGF-β1 and α-SMA, promoting renal fibrosis in DN (60). Furthermore, lncRNA-NEAT1, by adsorbing miR-34c, deregulated its inhibitory effect on NLRP3, which in turn activated NLRP3 inflammatory vesicles and promoted caspase-1-dependent pyroptosis and IL-1β release, exacerbating inflammation and kidney injury in DN (61).

In addition to the well-characterised sponge functions of lncRNAs, accumulating evidence reveals that circRNAs—also functioning as a chelator of cytoplasmic RNA-binding proteins—harbor multiple miRNA-binding sites and act as ceRNAs to modulate miRNA activity (62). Mechanistically, the circRNA-miRNA-mRNA regulatory network represents the prototypical operational paradigm, wherein circRNAs exert their biological effects by acting as miRNA sponges to alleviate miRNA-mediated suppression of target mRNAs (63). To date, this circRNA-miRNA-mRNA axis has not only been implicated in the pathogenesis of diverse disorders including malignancies, cardiovascular/cerebrovascular diseases, and bone metabolic abnormalities (64–66), but also in DN progression (**Figure 1**). Notably, circ_0000181 has been shown to competitively bind and downregulate miR-667-5p expression to promote NLRC4 inflammasome activation along with IL-1β and IL-18 release (67). These findings establish that circ_0000181 facilitates pyroptotic progression in DN through regulation of the miR-667-5p/NLRC4 axis. Similarly, circ_0000712 has been found to promote MC apoptosis, inflammation, OS and fibrosis by sponging miR-879-5p to upregulate SOX6 expression, thereby increasing the levels of Bax, IL-1β, IL-6, TNF-α, ROS, LDH, FN, Col-I, and Col-IV (68). Li et al. demonstrated that circ_0114428 exacerbates HG-induced MC proliferation, fibrosis, and EMT in DN by sequestering and downregulating expression of miR-185-5p while promoting SMAD3 expression (69). Beyond their pathogenic roles, circRNA_010383 has been shown to inhibit miR-135a activity by acting as a sponge, thereby up-regulating TRPC1—the target protein of miR-135a—inhibiting ECM FN accumulation, Col-I, and α-SMA, and ameliorating proteinuria, GMCs matrix expansion and kidney fibrosis, inhibiting DN progression, and exerting a protective effect against DN (52).

Collectively, the ceRNA hypothesis offers a novel perspective for disease research, facilitating more comprehensive insights into biological regulation. Targeting the lncRNA/circRNA–miRNA–mRNA axis not only provides new directions for exploring DN pathogenesis and therapeutic development but also contributes to elucidating the molecular functions of ncRNAs in DN pathogenesis.

## 3 ceRNA network and DN

Contemporary studies have confirmed that DN constitutes a primary etiological factor in the global burden of end-stage kidney failure (70). HG, recognized as a primary driver of DN progression, induces podocyte injury, mesangial cell proliferation and TECs damage through triggering inflammatory responses and directly or indirectly mediating OS, apoptosis, pyroptosis, and endoplasmic reticulum stress, these pathological processes culminate in ECM accumulation, renal fibrosis, basement membrane thickening and glomerular hypertrophy, ultimately reducing glomerular filtration rate (GFR), exacerbating proteinuria, and accelerating DN advancement (4–6, 15–20).

As a terminally differentiated epithelial cell situated outside the glomerular capillaries, Podocytes represent critical structural elements within the glomerular filtration apparatus, orchestrating precise permeability regulation through their dynamic control of filtration selectivity (71). Studies have shown that, owing to their limited regenerative and reparative capacities, podocytes undergo characteristic pathological changes – including foot process effacement and slit diaphragm disruption–when exposed to external stimuli such as HG and HG-induced inflammatory responses, OS, apoptosis, autophagy, and endoplasmic reticulum stress, ultimately leading to podocyte injury (72–74). The consequent impairment of the glomerular filtration barriers precipitates proteinuria and progressive renal function deterioration, a pathogenic cascade widely recognized as central to DN pathogenesis (75). Consequently, investigating therapeutic approaches to prevent podocyte injury or enhance cellular repair mechanisms may yield critical insights for identifying novel therapeutic targets and developing prospective strategies in DN management.

Aberrant proliferation of GMCs is recognized as another critical factor in the pathogenesis of DN (76). As the predominant cellular component of the glomerular mesangium, these cells not only synthesize matrix proteins but also play an indispensable role in regulating glomerular filtration through an interconnected fibrillar network, thus coordinating glomerular architectural stability with physiological performance homeostasis (77). Experimental evidence confirms that in HG environments, GMCs exhibit ectopic expression of cytokines and filamentous proteins, driving pathological MC proliferation and renal fibrosis (78). Therefore, targeting HG-induced MC dysfunction may offer therapeutic value in DN prevention. However, the molecular mechanisms underlying mesangial cell proliferation within the glomerular microenvironment remain largely uncharacterized. Thus, deeper investigation into MC biology is imperative for advancing targeted therapeutic strategies for DN.

The extent of renal tubular injury is a critical determinant of DN prognosis. As essential target cells, TECs—regulated by HG-induced OS, inflammatory responses, and apoptotic pathways—disrupt proximal tubule solute transport homeostasis and drive nephrogenic fibrogenesis, consequently exacerbating diabetic glomerulopathy trajectories (79, 80). However, current multipronged strategies focused on proteinuria control have failed to fully prevent DN development (81, 82). Elucidating the molecular mechanisms underlying HG-mediated tubular epithelial cell injury remains essential for identifying novel therapeutic approaches to combat DN.

Therefore, inflammation, OS, cell death (apoptosis and pyroptosis), and fibrosis caused by inflammation and OS are the primary pathways that lead to DN. Renal cells (podocytes, GMCs, and TECs) were the primary target tissues. Thus, the ceRNA network may be the key to treating DN by blocking cell death and fibrosis pathways and alleviating renal cell damage.

### 3.1 ceRNA network regulates renal cell progression of DN through intervention in cell death pathways

#### 3.1.1 ceRNA networks intervene in the regulation of DN by podocytes through cell death pathways

**Figure 2:**
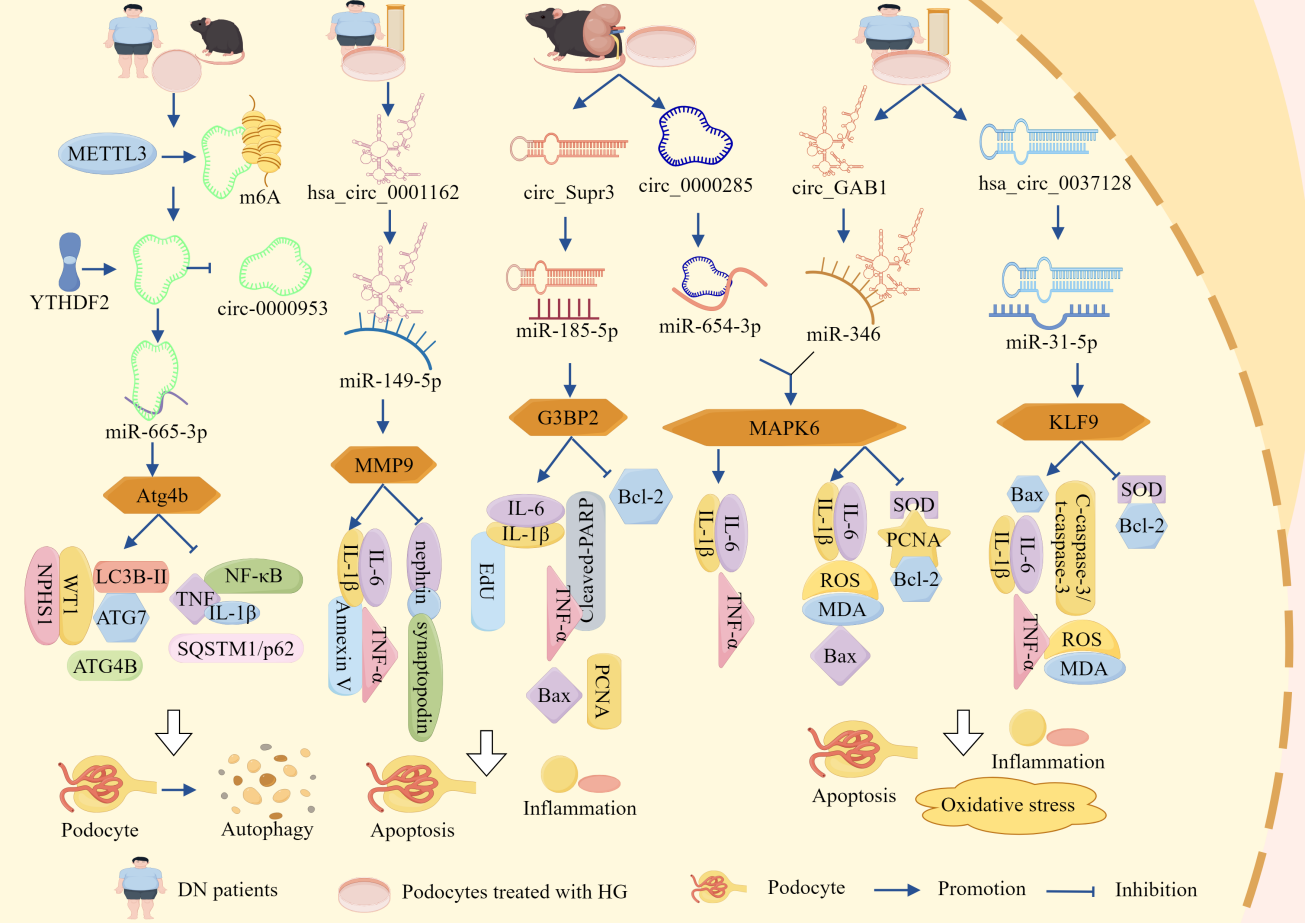
ceRNA networks influence podocyte-dependent regulation of DN via cell death pathways. LncRNA-DLX6-AS1, lncRNA-SNHG5, lncRNA-MIAT, lncRNA-TCF7, lncRNA-1500026H17Rik, lncRNA-XIST, hsa_circ_0001162, circ_Supr3, circ_0000285, circ_GAB1, and hsa_circ_0037128 respectively sponge miR-346-5p, miR-26a-5p, miR-130a-3p, miR-16-5p, miR-205-5p, miR-30, miR-149-5p, miR-185-5p, miR-654-3p, miR-346, and miR-31-5p. This promotes the expression of downstream miRNA target molecules GSK-3β, TRPC6, TLR4, SEMA3A, EGR1, AVEN, MMP9, G3BP2, MAPK6, and KLF9, thereby activating inflammation, OS, and apoptosis. Consequently, podocyte survival rate is reduced and podocyte injury is accelerated, ultimately exacerbating DN progression via cell death pathways.

In recent years, a large body of evidence has indicated that inflammation, OS, and apoptosis are the primary causes of diabetic kidney injury. The lncRNA/circRNA–miRNA–mRNA network plays a central role in promoting the development of diabetic nephropathy by regulating inflammation, OS, and apoptosis in podocytes (**Figure 2**, **Table 1**).

**Table 1:** The ceRNA network regulates renal cell progression of DN through intervention in cell death pathways.

Spongy molecule	miRNA	mRNA	Mechanism of action	Pathway	Study subjects	Functional experiments	Result	Refes
lncRNA- DLX6-AS1	346-5p	GSK-3β	Down-regulated: podocin, WT-1, SYNPO Up-regulated: IL-17	Cell death	Kidney tissue of DN patients and *db/db* mice	Loss	Aggravate inflammation and promote podocyte damage	(83)
lncRNA- SNHG5	26a-5p	TRPC6	Down-regulated: nephrin, podocin, Bax, caspase-3, Bcl-2 Up-regulated: TNF-α, IL-6, IL-1β		HG-treated podocytes and *db/db* mice	Gain and loss	Promotes podocyte inflammation and apoptosis	(84)
lncRNA- MIAT	130a-3p	TLR4	Down-regulated: Bax, caspase-3, Bcl-2 Up-regulated: TNF-α, IL-6, IL-1β		HG-treated podocytes	Loss	Increased the survival rate of podocytes, reduce the apoptosis rate and inflammatory reaction	(85)
lncRNA- TCF7	16-5p	SEMA3A	Down-regulated: Bcl-2, SOD, CAT Up-regulated: Bax, ROS, MDA, IL-1β, TNF-α, IL-6		Serum of DN patients	Loss	Leading to increased podocyte apoptosis, enhanced OS and inflammatory response, and decreased activity	(86)
lncRNA- 1500026H17Rik	205-5p	EGR1	Down-regulated: Bcl-2, Podocin, SOD Up-regulated: Bax, caspase-3, α-SMA, FN, Col-I, MDA, ROS, IL-6, IL-1β, TNF-α		DN mouse kidney tissue and HG-induced podocytes	Gain and loss	Promoted HG induced podocyte apoptosis, fibrosis, OS and inflammatory response	(87)
lncRNA- XIST	30	AVEN	Down-regulated: cytochrome c, Bax, caspase-3 Up-regulated: Bcl-2		HG-treated podocytes	Gain	Reduced the apoptosis of podocytes induced by HG and improve the cell survival rate	(88)
hsa_circ_ 0001162	149-5p	MMP9	Down-regulated: Annexin V, IL-6, TNF-α, IL-1β Up-regulated: nephrin, synaptopodin	Cell death	Peripheral blood of DN patients and HG-treated podocytes	Loss	Inhibition the apoptosis of podocytes induced by HG and alleviated the inflammatory reaction	(89)
circ_ Supr3	185-5p	G3BP2	Down-regulated: Bcl-2 Up-regulated: EdU, PCNA, Bax, Cleaved-PARP, TNF-α, IL-6, IL-1β		Kidney tissues of DM mice and HG-treated MPC5 cells	Gain and loss	Inhibition of podocyte apoptosis and inflammatory response	(90)
circ_ 0000285	654-3p	MAPK6	Down-regulated: apoptosis Up-regulated: IL-6, IL-1β, TNF-α		Kidney tissues of DM mice and HG-treated podocytes	Gain and loss	Inhibition of podocyte proliferation and promotion of podocyte apoptosis	(91)
circ_ GAB1	346	MAPK6	Down-regulated: SOD, PCNA, Bcl-2 Up-regulated: IL-6, TNF-α, ROS, MDA, caspase-3, Bax		DN patient serum and HG-treated podocytes	Gain and loss	Inhibition HG induced apoptosis, inflammatory reaction and OS, and promote cell proliferation	(92)
hsa_circ_ 0037128	31-5p	KLF9	Down-regulated: Bcl-2, SOD Up-regulated: Bax, C-caspase-3/ t-caspase-3, TNF-α, IL-1β, IL-6, ROS, MDA		DN patient serum and HG-treated podocytes	Gain and loss	Aggravation HG induced podocyte apoptosis, inflammation and OS.	(93)
lncRNA- XIST	423-5p	HMGA2	Up-regulated: apoptosis		Kidney tissue of DN patients and HG-treated HK-2 cells	Gain and loss	Promoted HG-induced renal cell proliferation and inhibit apoptosis	(94)
lncRNA- TUG1	29c-3p	SIRT1	Down-regulated: apoptosis, GRP78, caspase-12, CHOP, p-PERK/p-Eif-2α		HG-treated HK-2 cells	Gain	Inhibited HG-induced cell proliferation apoptosis and endoplasmic reticulum stress	(95)
lncRNA- SNHG5	26a-5p	/	Down-regulated: eGFR, albumin Up-regulated: FBG, IL-6, TNF-α, proteinuria, ROS	Cell death	DN patient serum and HG-treated HK-2 cells	Loss	Promoted inflammation, OS and apoptosis	(84)
lncRNA- MALAT1	30c	NLRP3	Up-regulated: caspase-1, IL-1β, IL-18, LDH		HG-treated HK-2 cells	Gain and loss	Promoted inflammation and pyroptosis	(96)
lncRNA- UCA1	206	/	Down-regulated: caspase-1, IL-1β, NLRP3		DN rat renal tubular epithelial tissue and HG-treated HK-2 cells	Gain and loss	Mitigated apoptosis and reduced inflammatory response	(97)
circ_ 0003928	506-3p	HDAC4	Down-regulated: SOD, Bcl-2 Up-regulated: ROS/MDA, Bax, caspase-3		DN patients and HG-treated HK-2 cells	Gain and loss	Promoted HG-induced OS and apoptosis	(98)
circ_ 0000064	532-3p		Down-regulated: MDA, Bcl2 Up-regulated: Bax, caspase-3, α-SMA, Col-I, SOD		DN patient serum and HG-treated HK-2 cells	Gain and loss	Promoted HG-induced OS, apoptosis and fibrosis	(99)

Clinical trials have documented a marked elevation of lncRNA-DLX6-AS1 in renal biopsies from patients with DN, demonstrating a quantitative correlation between transcript abundance and urinary albumin–creatinine ratio (uACR) progression, thereby implicating DLX6-AS1 in DN progression (83). Further animal studies demonstrated that markedly elevated DLX6-AS1 expression in *db/db* murine models was associated with reduced podocyte integrity markers (podocin, WT-1, and SYNPO) and concomitant increases in the pro-inflammatory cytokine IL-17. These data mechanistically link DLX6-AS1 overexpression to podocyte injury, inflammatory exacerbation, and proteinuria, whereas DLX6-AS1 knockout (KO) substantially attenuates these pathological manifestations (83). Mechanistically, miR-346-5p directly targets the 3′ untranslated region (UTR) of GSK-3β, whereas DLX6-AS1 competitively sequesters miR-346-5p, thereby derepressing GSK-3β expression. This regulatory axis drives podocyte dysfunction and inflammatory cascades, thereby accelerating the pathogenesis of DN. Consequently, therapeutic strategies targeting DLX6-AS1 suppression or modulation of its downstream signaling pathways may be promising for alleviating DN pathology (83).

Additional studies revealed significantly elevated expression of lncRNA-SNHG5 and TRPC6 in the kidney tissues of *db/db* mice and HG-treated podocytes, concomitant with marked reductions in miR-26a-5p levels and the podocyte-specific markers, nephrin and podocin. Functional studies demonstrated that miR-26a-5p overexpression attenuated TRPC6 expression and mitigated podocyte apoptosis and inflammation, whereas KO-SNHG5 upregulated miR-26a-5p, suppressed TRPC6, and significantly ameliorated proteinuria, restored podocyte ultrastructure, and alleviated HG-induced podocyte injury in *db/db* mice (84). These findings indicate that SNHG5 acts as a molecular sponge for miR-26a-5p, thereby derepressing TRPC6 expression and driving podocyte dysfunction and DN progression. In addition, lncRNA-MIAT expression was markedly upregulated in HG-stimulated podocytes, whereas miR-130a-3p expression was suppressed, MIAT silencing substantially reduces HG-induced TNF-α, IL-6, and IL-1β secretion; downregulates pro-apoptotic markers, Bax and caspase-3; and upregulates anti-apoptotic marker, Bcl-2, thereby promoting podocyte survival under HG conditions (85). Mechanistically, KO-MIAT elevates miR-130a-3p expression, which subsequently inhibits TLR4 signaling and attenuates HG-mediated inflammation and apoptosis in podocytes (85). Therefore, MIAT functions as a sponge for miR-130a-3p and regulates TLR4 expression by competitively binding to miR-130a-3p. Inhibition of MIAT or modulation of its downstream pathway could help attenuate the pathological changes in DN.

Clinical investigations revealed significant downregulation of miR-16-5p and a concurrent rise of lncRNA-TCF7 and SEMA3A in serum samples from patients with DN, with these aberrant expression profiles demonstrating positive correlations with hyperglycemic indices (blood glucose and glycated hemoglobin) and uACR (86). Experimental studies have further shown that HG conditions elevate TCF7 and SEMA3A expression, while suppressing miR-16-5p, but also increase Bax, ROS, MDA, IL-1β, TNF-α, and IL-6. Concurrently, HG exposure reduced anti-apoptotic Bcl-2 expression and antioxidant enzyme activity (SOD and CAT), culminating in podocyte apoptosis, OS exacerbation, inflammatory activation, and diminished cellular viability. The genetic silencing of TCF7 or SEMA3A reversed these pathological alterations (86). Notably, TCF7 acts as a negative regulator of miR-16-5p and a positive modulator of SEMA3A, the latter being a direct miR-16-5p target. miR-16-5p directly binds to the 3′ UTR of SEMA3A to suppress its expression, establishing a regulatory triad. Collectively, these findings indicated that lncRNA-TCF7 functions as a ceRNA, sequestering miR-16-5p and derepressing SEMA3A expression (86). This axis drives HG-induced podocyte injury by amplifying OS, apoptosis, and inflammatory cascades, thereby positioning the TCF7/miR-16-5p/SEMA3A axis as a potential therapeutic target in DN.

Xia et al. showed that lncRNA-1500026H17Rik and EGR1 were significantly elevated, miR-205-5p was significantly reduced in the kidney tissues and HG-induced podocytes of DN mice, and 1500026H17-Rik was positively correlated with EGR1 and negatively correlated with miR-205-5p (87). KO-1500026H17Rik significantly downregulated Bax; cleaved caspase-3, α-SMA, FN, Col I, MDA, ROS, IL-6, IL-1β, and TNF-α expression; and upregulated Bcl-2, Podocin, and SOD. It also attenuates apoptosis, fibrosis of HG-induced podocytes, OS, and inflammatory responses, whereas inhibition of miR-205-5p or overexpression of EGR1 reversed the protective effects of KO-1500026H17Rik. In addition, 1500026H17Rik acts as a ‘sponge’ for miR-205-5p and inhibits miR-205-5p activity, whereas EGR1 is a downstream target of miR-205-5p and can be directly targeted by it to inhibit EGR1 expression (87). It has been shown that lncRNA-1500026H17Rik, by adsorbing miR-205-5p, deregulates the inhibition of EGR1, which in turn exacerbates podocyte injury.

Emerging evidence has indicated that although certain lncRNA–miRNA networks drive DN progression, others may exert protective effects. For instance, studies have demonstrated that HG conditions significantly impair podocyte survival and enhance apoptotic rates, concomitant with the downregulation of lncRNA-XIST expression. Notably, XIST overexpression not only attenuated HG-induced podocyte apoptosis and improved cellular viability but also suppressed the pro-apoptotic mediators such as cytochrome c, Bax, and caspase-3, while upregulating anti-apoptotic Bcl-2 expression (88). Mechanistically, miR-30 overexpression suppressed XIST, thereby abolishing its cytoprotective effects and promoting apoptosis, whereas miR-30 inhibition restored XIST-mediated podocyte protection. Furthermore, XIST overexpression rescues AVEN expression, a direct miR-30 target, by establishing a regulatory axis central to this pathway (88). These findings collectively suggest that lncRNA-XIST is a molecular sponge for miR-30 that modulates AVEN expression to govern podocyte survival and apoptosis. This axis highlights the potential therapeutic relevance of XIST in DN and offers novel targets for intervention.

In addition to the lncRNA–miRNA–mRNA network, the circRNA–miRNA–mRNA network had the same function (**Figure 2**, **Table 1**). It has been reported that that hsa_circ_00162 was upregulated in the peripheral blood and HG-induced podocytes of patients with DN. KO-hsa_circ_00162 suppressed MMP9 expression; downregulated IL-6, TNF-α, and IL-1β expression; restored nephrin and synaptopodin expression; inhibited HG-induced podocyte apoptosis; attenuated the inflammatory response; and alleviated podocyte injury (89). In addition, miR-149-5p inhibition reversed the inhibitory effect of KO-hsa_circ_0001162 on MMP9, which in turn upregulated MMP9 expression by adsorbing miR-149-5p to deregulate its inhibition of MMP9 (89). Thus, hsa_circ_0001162 has been reported to promote DN podocyte injury by regulating MMP9 expression through a ceRNA mechanism, which lays the groundwork for the molecular diagnosis and targeted therapy of DN.

Circ_Supr3 and G3BP2 were significantly upregulated and miR-185-5p was significantly downregulated in the kidney tissues of DM mice and HG-treated MPC5 cells in a previous study. KO-circ_Supr3 upregulated miR-185-5p expression and thus inhibited G3BP2; reversed HG-induced cell proliferation; and downregulated EdU, PCNA, Bax, Cleaved-PARP, TNF-α, IL-6, and IL-1β expression; increased Bcl-2 expression; and inhibited apoptosis and inflammatory responses, whereas G3BP2 overexpression counteracted the protective effect of miR-185-5p on HG-induced injury, and miR-185-5p inhibitor in turn reversed the protective effect of KO-circ_Supr3 on cell injury (90). Circ_Supr3 exacerbates HG-induced podocyte injury by adsorbing miR-185-5p to upregulate G3BP2 expression. Similarly, in the kidney tissues of the STZ-induced DN mouse model and HG-treated podocytes, circ_0000285 expression was significantly elevated and miR-654-3p expression was significantly reduced, overexpression of circ_0000285 inhibited podocyte proliferation, promoted podocyte apoptosis, and blocked the cell cycle. KO-circ_0000285 inhibited the release of IL -6, IL-1β, and TNF-α, whereas inhibition of miR-654-3p reversed the protective effect of KO-circ_0000285 on podocyte injury (91). In addition, MAPK6 is a direct target of miR-654-3p, and circ_0000285 activates the MAPK6 signaling pathway through the adsorption of miR-654-3p, leading to podocyte injury, an inflammatory response, and promotion of DN progression (91).

Research has identified significantly elevated expression levels of circ_GAB1 and MAPK6 in the serum of both patients with DN and HG-treated podocytes, concomitant with a marked reduction in miR-346 expression (92). Silencing circ_GAB1 suppressed MAPK6 expression, resulting in decreased levels of IL-6, TNF-α, ROS, MDA, cleaved caspase-3, and Bax within podocytes, conversely, this intervention elevated SOD, PCNA, and Bcl-2 expression, thereby mitigating HG-induced apoptosis, inflammatory responses, and OS, while promoting cellular proliferation and attenuating podocyte injury, notably, the administration of miR-346 inhibitors reversed the cytoprotective effects of KO-circGAB1. Furthermore, both miR-346 overexpression and MAPK6 suppression alleviated podocyte damage (92). These findings collectively indicated that circGAB1 acts as a molecular sponge for miR-346, thereby relieving its inhibitory effect on MAPK6 and subsequently activating the MAPK6-mediated pathways of podocyte apoptosis, inflammation, and OS mechanisms that ultimately drive DN progression.

In addition, Fang et al. demonstrated that sera from patients with DN and HG-induced podocytes exhibited significant upregulation of KLF9 and hsa_circ_0037128, along with marked downregulation of miR-31-5p. A negative correlation was observed between hsa_circ_0037128 and miR-31-5p, whereas a positive correlation existed with KLF9, KO-hsa_circ_0037128 reduces Bax and caspase-3 levels and increases Bcl-2 expression in HG-stimulated podocytes, this intervention suppressed HG-induced podocyte apoptosis as well as decreased inflammatory mediators such as TNF-α, IL-1β, and IL-6, reduced OS markers (ROS and MDA), and enhanced SOD activity, thereby collectively ameliorating podocyte injury (93). This study further revealed that miR-31-5p is a direct target of hsa_circ_0037128 and that KLF9 functions as a downstream target of miR-31-5p, notably, miR-31-5p inhibition or KLF9 overexpression counteracted the protective effects of KO-hsa_circ_0037128 in podocytes (93). These findings collectively indicate that hsa_circ_0037128 acts as a competitive endogenous RNA by sequestering miR-31-5p, thereby relieving the miR-31-5p-mediated suppression of KLF9. Subsequent KLF9 upregulation exacerbated HG-induced inflammatory responses, OS, and podocyte apoptosis.

Although quantitative studies have questioned the physiological effects of ceRNA interactions under typical conditions, the above-mentioned ceRNA network studies were conducted using *in vivo* and *in vitro* models that were functionally validated and supported by loss-of-function and gain-of-function experiments.

#### 3.1.2 ceRNA networks intervene in the regulation of DN by TECs through cell death pathways

**Figure 3:**
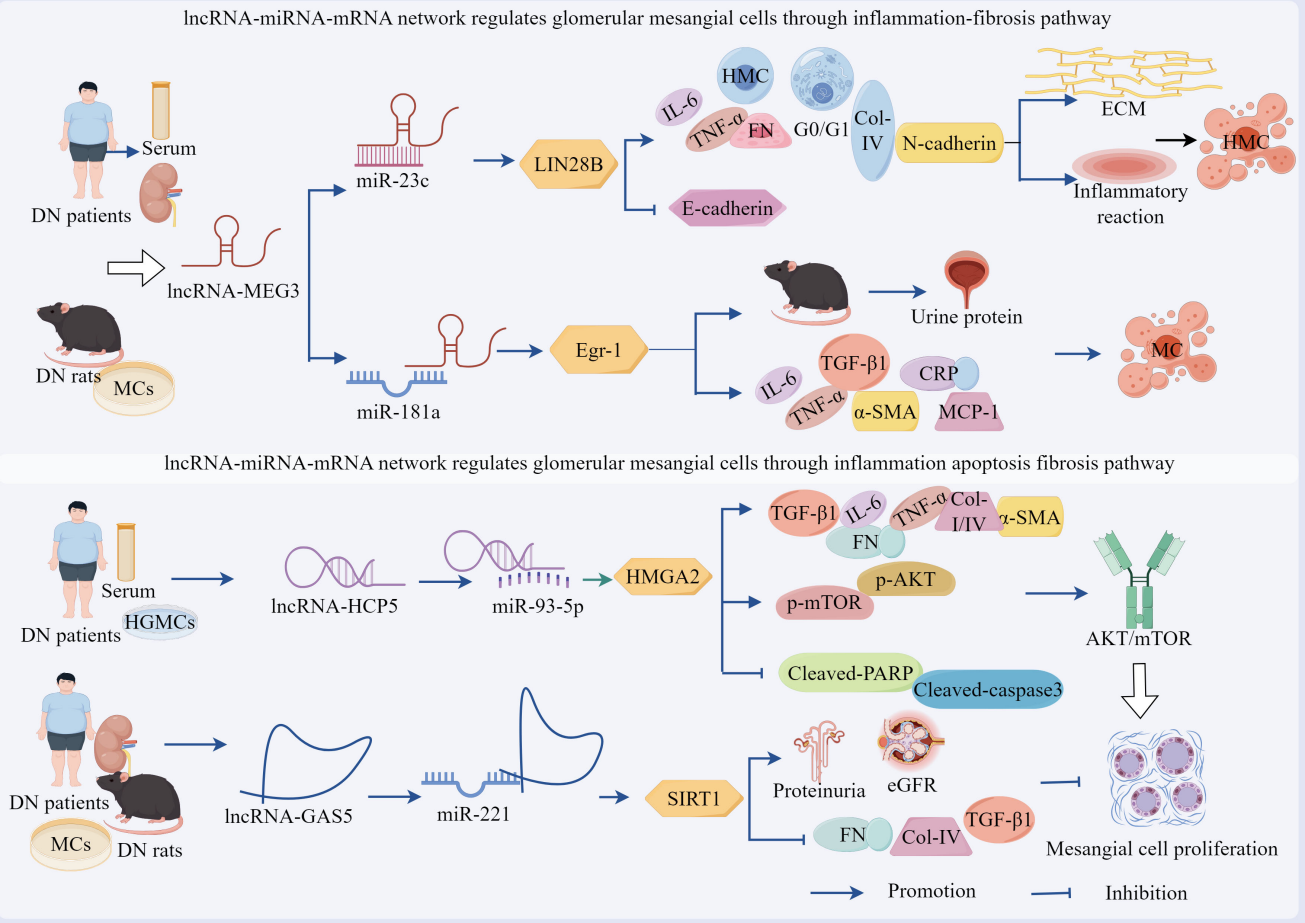
ceRNA networks intervene in the regulation of DN by TECs through cell death pathways. lncRNA-XIST sponges miR-423-5p to upregulate HMGA2 expression, promoting HG-induced proliferation of HK2 cells while suppressing apoptosis. lncRNA-TUG1 acts as a molecular sponge for miR-29c-3p, upregulating SIRT1 to inhibit cellular apoptosis and attenuate DN progression. lncRNA-SNHG5 and lncRNA-UCA1 accelerate DN progression by directly upregulating factors associated with inflammation, OS, and apoptosis through sponging miR-26a-5p and miR-206, respectively. lncRNA-MALAT1 targets miR-30c and upregulates NLRP3, promoting increased expression of inflammatory and apoptotic factors that exacerbate cellular injury. Additionally, circ_0003928 and circ_0000064 exacerbate TECs injury and DN progression by sponging miR-506-3p and miR-532-3p to upregulate HDAC4 and ROCK1 expression, respectively, thereby promoting apoptosis and OS.

In addition to its regulatory effects on podocytes in DN progression through cell death pathways, the lncRNA–miRNA–mRNA network also modulated TECs to influence disease progression (**Figure 3**, **Table 1**). Investigations revealed a dose-dependent upregulation of lncRNA-XIST and HMGA2 in the renal tissues of patients with DN and HG-treated renal tubular HK-2 cells. Silencing of XIST or HMGA2 inhibited HG-induced cellular proliferation and enhanced apoptosis. Dual-luciferase reporter assays further demonstrated direct binding between XIST and miR-423-5p, with miR-423-5p targeting the 3′ UTR of HMGA2, KO-XIST upregulated miR-423-5p expression, consequently reducing HMGA2 levels—an effect partially reversed by miR-423-5p inhibition (94). Furthermore, miR-423-5p overexpression suppresses HMGA2 expression, attenuates cellular proliferation, and promotes apoptosis. These effects were counteracted by HMGA2 overexpression (94). Collectively, these findings indicate that lncRNA-XIST functions as a molecular sponge to sequester miR-423-5p, thereby derepressing HMGA2 expression. This regulatory axis promotes HG-driven renal cell proliferation, while suppressing apoptotic processes during DN pathogenesis.

The lncRNA–miRNA–mRNA network exhibits dual regulatory capacities in DN, demonstrating both promotive and suppressive effects on disease progression. Studies revealed that HG treatment significantly increased apoptotic rates and upregulated eERS-associated proteins GRP78, caspase-12, CHOP, p-PERK, p-eIF2α in HK-2 cells, concurrent with lncRNA-TUG1 downregulation, TUG1 overexpression suppressed miR-29c-3p expression, markedly attenuated HG-induced apoptosis, and reduced ERS-related protein levels (95). Notably, miR-29c-3p overexpression exacerbated HG-driven apoptosis and ERS protein expression, counteracting TUG1-mediated protection; this effect was reversed by miR-29c-3p inhibition. Mechanistic investigations have identified SIRT1 as a direct target of miR-29c-3p, whose expression is maintained through TUG1-dependent miR-29c-3p suppression (95). Collectively, these findings demonstrate that lncRNA-TUG1 functions as a molecular sponge to inhibit miR-29c-3p activity, thereby preserving SIRT1 expression and mitigating ERS-mediated cellular injury.

Tubular epithelial cell dysfunction is a pivotal mechanism in DN pathogenesis, in which inflammatory–apoptotic/pyroptotic signaling cascades contribute to dysregulated tubular reabsorptive processes and fibrotic changes in the kidney, leading to pathological advancement (**Figure 3**, **Table 1**). Emerging evidence highlights the critical regulatory role of ncRNAs in the modulation of these pathological processes. Studies have identified the significant upregulation of lncRNA-SNHG5 and concomitant downregulation of miR-26a-5p in the sera from patients with DN and HG-treated HK-2 cells, SNHG5 exhibited negative correlations with miR-26a-5p, eGFR, and albumin levels, whereas positive associations with fasting blood glucose, IL-6, TNF-α, and proteinuria. SNHG5 inhibition reversed HG-induced reductions in cell viability, attenuated apoptotic rates, and normalized IL-6, TNF-α, and ROS levels (84). Mechanistic investigations confirmed that miR-26a-5p is a direct target of SNHG5. SNHG5 silencing upregulates miR-26a-5p expression and ameliorates HG-induced tubular injury. Collectively, these findings demonstrate that lncRNA-SNHG5 exacerbates HG-mediated tubular damage through miR-26a-5p sequestration (84). Targeted SNHG5 suppression mitigates inflammatory responses, OS, and apoptotic signaling, thereby identifying novel therapeutic targets for DN.

Furthermore, Liu et al. demonstrated that HG treatment significantly upregulated lncRNA-MALAT1 alongside NLRP3, caspase-1, IL-1β, and IL-18 expression, while suppressing miR-30c levels in HK-2 cells. This dysregulation elevates pyroptotic rates and lactate dehydrogenase (LDH) release, MALAT1 silencing markedly attenuated HG-induced pyroptosis and reduced NLRP3 inflammasome components such as caspase-1, IL-1β, and IL-18, whilst restoring miR-30c expression—suggesting that KO-MALAT1 suppresses hyperglycemia-induced pyroptosis through miR-30c upregulation and NLRP3 downregulation (96). Dual-luciferase reporter assays confirmed direct binding between MALAT1 and miR-30c, with miR-30c targeting the 3′ UTR of NLRP3. miR-30c overexpression suppresses NLRP3 expression and ameliorates pyroptosis and inflammatory responses. Critically, co-transfection of MALAT1 with miR-30c inhibitors reversed the anti-pyroptotic effects of MALAT1 silencing (96). These findings establish that lncRNA-MALAT1 exacerbates inflammatory pyroptosis by sequestering miR-30c, thereby derepressing NLRP3 expression. Targeted disruption of the MALAT1/miR-30c/NLRP3 axis may provide novel therapeutic opportunities for the management of DN.

Further investigation revealed significant downregulation of lncRNA-UCA1 and concomitant upregulation of miR-206 in the renal tubular epithelial tissues of DN rats and HG-treated HK-2 cells, with an inverse correlation between miR-206 and UCA1 expression. UCA1 overexpression markedly reduced protein levels of pyroptosis-associated markers caspase-1, IL-1β, and NLRP3 under HG conditions, thereby alleviating apoptotic cell death and inflammatory responses. Conversely, KO-UCA1 exacerbated these pathological manifestations (97). Mechanistic validation using dual-luciferase reporter assays identified binding complementarity between UCA1 and miR-206. miR-206 mimics counteract UCA1 overexpression-mediated suppression of apoptosis, whereas miR-206 inhibition partially rescues the pro-apoptotic effects induced by UCA1 silencing (97). These findings demonstrated that lncRNA-UCA1 exerts cytoprotective effects by directly binding to and suppressing miR-206 activity.

The circRNA–miRNA–mRNA network regulates DN progression through the apoptosis–OS pathway, demonstrating dual therapeutic potential in both podocytes and TEC (**Figure 3**, **Table 1**). Experimental evidence revealed significant upregulation of circ_0003928 and HDAC4, along with marked downregulation of miR-506-3p in patients with DN and in HG-treated models. In HG-stimulated HK-2 cells, the characteristic pathological manifestations include reduced cell viability, elevated ROS and MDA levels, diminished SOD activity, upregulation of Bax and caspase-3 expression, and downregulation of Bcl-2 expression (98). Notably, KO-circ_0003928 ameliorated HG-induced reduction in cell viability and proliferation, attenuated ROS and MDA generation, restored SOD activity, and reduced apoptosis. Mechanistic studies further established that circ_0003928 silencing upregulates miR-506-3p expression, whereas miR-506-5p overexpression significantly suppresses HDAC4 expression at both the transcriptional and translational levels (98). Collectively, these findings demonstrated that circ_0003928 exacerbates OS and apoptosis by competitively sequestering miR-506-3p, thereby relieving its inhibitory effect on HDAC4. This novel mechanistic insight delineates the role of the circ_0003928/miR-506-3p/HDAC4 regulatory axis in pathogenesis of DN.

Wang et al. demonstrated that circ_0000064 is significantly upregulated in both serum samples from patients with DN and HG-stimulated HK-2 cells, concomitant with the downregulation of miR-532-3p and that circ_0000064 expression is positively correlated with disease severity (albuminuria levels) (99). Experimental interventions showed that either circ_0000064 silencing or miR-532-3p overexpression attenuated HG-induced pathological manifestations by reducing MDA activity; downregulating pro-apoptotic Bax, caspase-3, pro-fibrotic α-SMA, and Col-I markers; elevating SOD and anti-apoptotic Bcl-2 levels; mitigating OS, apoptosis, and fibrosis, while enhancing cellular proliferation. Notably, these protective effects were reversed by miR-532-3p inhibitors or ROCK1 overexpression, confirming the functional interplay within this regulatory axis (99). Mechanistic analyses revealed that under HG conditions, circ_0000064 acts as a competitive endogenous RNA by sponging miR-532-3p, thereby relieving its post-transcriptional repression of ROCK1 and leading to ROCK1 upregulation, which subsequently activates OS-related pathways, apoptotic cascades, and fibrotic signaling, collectively exacerbating tubular epithelial injury and DN progression. This study delineated the pathomechanistic landscape of circRNA-driven disease progression, establishing the circ_0000064/miR-532-3p/ROCK1 molecular interplay as a druggable pathway for DN intervention through multimodal regulatory network disruption (99).

In conclusion, the lncRNA/circRNA–miRNA–mRNA network exerts significant regulatory effects on podocytes and TECs through cell death, inflammation, OS, and apoptosis, with the cell death pathway serving as the common terminal pathway for ceRNA action. Regardless of their specific molecular mechanisms, all ceRNA networks studied ultimately converged directly to exacerbate or alleviate inflammatory responses, OS, and apoptosis in podocytes and TECs. These three processes constitute an inseparable ‘trinity’ of terminal effect axes through which ceRNA networks cause podocyte and TECs damage and DN progression, providing new insights into the molecular mechanisms and therapeutic strategies for DN. However, although the aforementioned ceRNA networks have been functionally validated and their interactions have been supported by loss-of-function/gain-of-function experiments, the roles of the lncRNA-TUG1/miR-29c-3p/SIRT1 and lncRNA-MALAT1/miR-30c/NLRP3 axes have only been validated *in vitro* and lack *in vivo* experiments. Therefore, their robustness and roles need to be further verified.

### 3.2 ceRNA network mediates the regulatory role of GMCs in the progression of DN through fibrotic pathways

**Figure 4:**
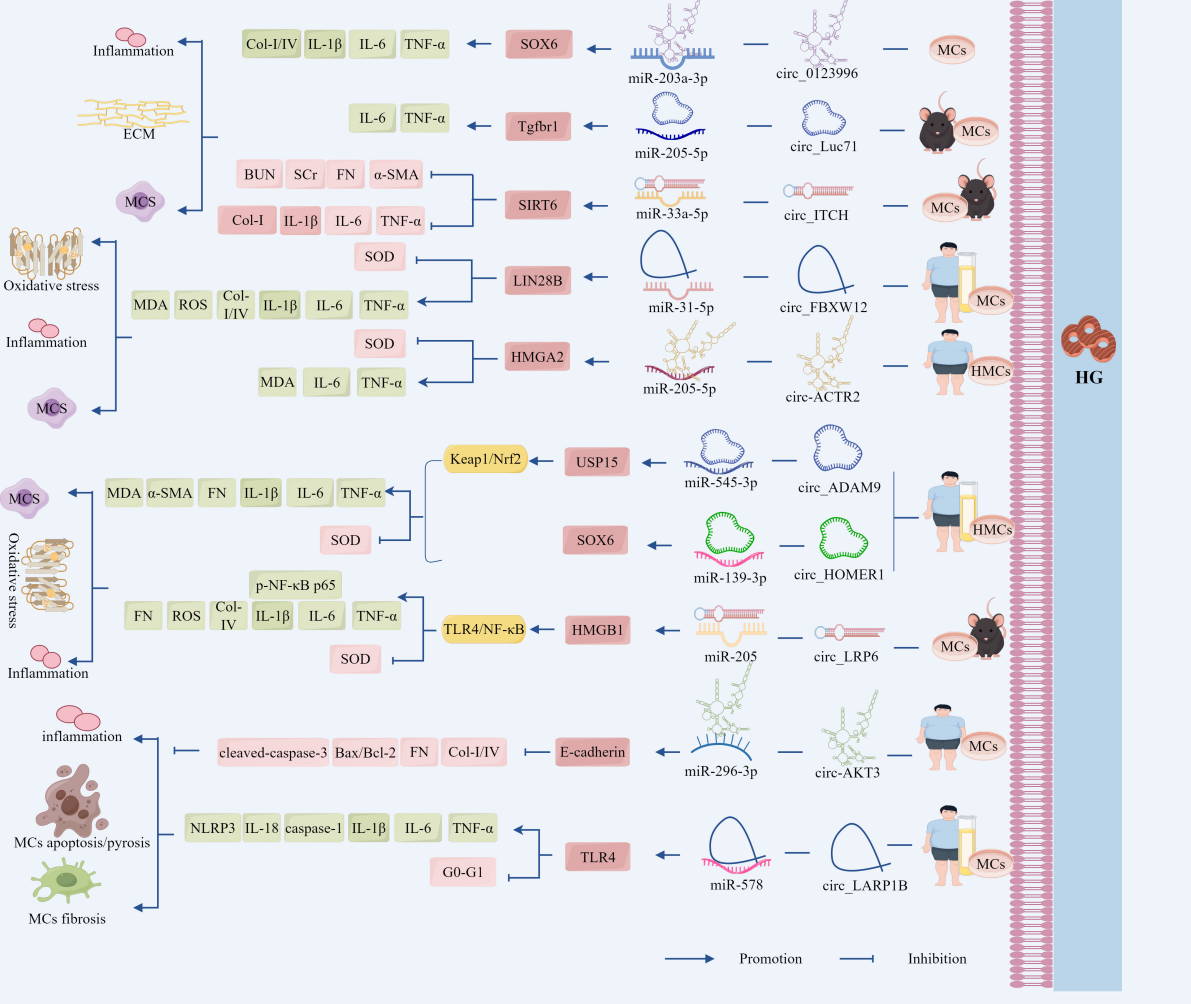
The ceRNA network mediates the regulatory role of GMCs in the progression of DN through fibrotic pathways. LncRNA-MEG3 deregulated its inhibition of LIN28B and Egr-1 by adsorbing miR-23c and miR-181a, respectively, leading to the up-regulation of LIN28B and TLR4 expression, which promoted fibrotic and inflammatory responses in DN. Circ_0123996, circ_Luc71 deregulated SOX6 and Tgfbr1 by adsorbing miR-203a-3p and miR-205-5p, respectively, which promoted DN progression through the inflammatory and fibrotic. However, circ_ITCH up-regulated SIRT6 expression and inhibited DN progression by adsorbing miR-33a-5p. In addition, circ_FBXW12, circ_ACTR2, circ_ADAM9, circ_HOMER1, and circ_LRP6 deregulated LIN28B, HMGA2, USP15, SOX6, and SIRT6 expression by adsorbing miR-31-5p, miR-205-5p, miR-545-3p, miR-137, and miR-205, respectively, which deregulated LIN28B, HMGA2, USP15, SOX6 and HMGB1 inhibition, increased the expression of inflammation, OS, and fibrosis-related factors, activated the fibrosis pathway, and regulated DN progression.

Emerging evidence demonstrates that the lncRNA–miRNA network exerts regulatory effects on podocytes as well as plays a pivotal role in modulating MCs (**Figure 4**, **Table 2**). Studies have revealed a significant upregulation of lncRNA-MEG3 and concomitant downregulation of miR-23c in serum samples from patients with DN, renal tissues, and HG-treated human MCs (HMCs), with a negative correlation observed between these two molecules, silencing of lncRNA-MEG3 suppressed HG-induced HMC proliferation and induced G0–G1 phase cell cycle arrest, while reducing the expression levels of fibrotic markers FN and Col-IV, inflammatory cytokines IL-6 and TNF-α, and mesenchymal marker N-cadherin. Concurrently, this intervention elevated E-cadherin expression, attenuated ECM accumulation, mitigated inflammatory responses, and reversed EMT, thereby ameliorating HMC injury (100). Importantly, miR-23c inhibition abolished the protective effects of lncRNA-MEG3 silencing in HMCs. Mechanistically, miR-23c overexpression suppressed LIN28B expression, whereas LIN28B overexpression neutralized the miR-23c-mediated protective effects. Collectively, these findings indicated that lncRNA-MEG3 functions as a ceRNA by sequestering miR-23c, thereby relieving its inhibitory effect on LIN28B, the subsequent upregulation of LIN28B drives HMC proliferation, ECM deposition, and inflammatory cascades (100).

**Table 2:** The ceRNA network mediates the regulatory role of GMCs in the progression of DN through fibrotic pathways.

Spongy molecule	miRNA	mRNA	Mechanism of action	Pathway	Study subjects	Functional experiments	Result	Refes
lncRNA- MEG3	23c	LIN28B	Down-regulated: E-cadherin Up-regulated: FN, Col-IV, IL-6, TNF-α, N-cadherin	Fibrosis	Serum, kidney tissue of DN patients and HG-treated HMCs	Gain and loss	Promoted HMC proliferation, ECM accumulation and inflammatory response	(100)
lncRNA- MEG3	181a	Egr-1	Up-regulated: TNF-α, α-SMA, TGF-β1, CRP, IL-1β, IL-6, MCP-1, TLR4		DN rat and HG-stimulated MCs	Gain and loss	Promoted fibrosis and inflammatory response	(101)
circ_ 0123996	203a-3p	SOX6	Up-regulated: IL-6, IL-1β, TNF-α, Col-I/IV	Fibrosis	HG-treated GMCs	Gain and loss	Promoted HG-induced GMCs proliferation, inflammation and fibrosis	(102)
circ_ Luc71	205-5p	Tgfbr1	Up-regulated: IL-6, TNF-α, ECM		HG-treated GMCs and *db/db* mice	Loss	Promoted GMCs proliferation, inflammatory response and ECM accumulation	(103)
circ_ ITCH	33a-5p	SIRT6	Up-regulated: BUN, SCr, α-SMA, Col-I, FN, IL-6, IL-1β, TNF-α		DM mouse kidney tissue and HG-treated GMCs	Gain and loss	Slows down fibrosis and inflammatory response	(104)
circ_ FBXW12	31-5p	LIN28B	Down-regulated: SOD Up-regulated:IL-6, TNF-α, Col-I/IV, TGF-β1, MDA		Serum from DN patients and HG-treated HMCs	Gain and loss	Promoted inflammatory response, fibrosis, OS	(105)
Circ_ ACTR2	205-5p	HMGA2	Up-regulated: IL-6, TNF-α, ECM, SOD, MDA		Kidney tissue of DN patients and HG-treated HRMCs	Gain and loss	Reduces inflammatory response and OS	(106)
circ_ ADAM9	545-3p	USP15	Down-regulated: SOD, Nrf2 Up-regulated: IL-1β, IL-6, TNF-α, α-SMA, FN, Col-I/IV, MDA, Keap1		Serum from DN patients and HG-treated HMCs	Gain and loss	Induced OS, inflammation and ECM deposition in HMCs	(107)
circ_ HOMER1	137	SOX6						(108)
circ_ LRP6	205	HMGB1	Down-regulated: SOD Up-regulated: TLR4, p-NF-κB, p65, ROS, FN, Col-IV, IL-6, TNF-α, IL-1β		HG-treated HMCs	Gain and loss	Induced OS, ECM accumulation and inflammatory response	(109)

Zha et al. demonstrated a significant upregulation of lncRNA-MEG3 in both DN rat models and HG-stimulated GMCs, showing positive correlations with hyperglycemic severity and disease progression. MEG3 overexpression exacerbated pathological manifestations in DN rats, including elevated urinary albumin excretion rates, upregulated expression of fibrotic markers TNF-α, α-SMA, and TGF-β1, as well as that of inflammatory mediators CRP, IL-1β, IL-6, and MCP-1 (101). In addition, histological damage was aggravated and, conversely, KO-MEG3 produced opposing effects. Notably, miR-181a inhibition phenocopied MEG3 overexpression, whereas miR-181a restoration or KO-Egr-1 effectively counteracted MEG3-mediated effects (101). Mechanistically, lncRNA-MEG3 directly binds to and inhibits miR-181a functionality, with miR-181a normally suppressing its target gene, Egr-1. The subsequent Egr-1 activation promotes fibrotic and inflammatory responses through activation of the TLR4 signaling pathway (101). These findings collectively reveal that lncRNA-MEG3 functions as a molecular sponge for miR-181a, thereby relieving the miR-181a-mediated suppression of Egr-1. This cascade ultimately upregulates TLR4 expression, which drives renal fibrosis and inflammatory progression in patients with DN.

Therefore, lncRNA-MEG3 can not only adsorb miR-23c in a sponge-like manner, but also adsorb miR-181a, thereby regulating different miRNA downstream target molecules and interfering with the proliferation and fibrosis process of GMCs. Thus, further investigation is required to determine whether the same lncRNA can adsorb different miRNAs and thereby interfere with the disease process.

Recent advances in ceRNA network research have revealed the critical regulatory role of circRNA–miRNA–mRNA axes in modulating GMCs behavior during DN progression (**Figure 4**, **Table 2**). For instance, studies have identified significant upregulation of circ_0123996 and the transcription factor SOX6, accompanied by miR-203a-3p downregulation in HG-treated GMCs. Silencing circ_0123996 suppressed SOX6 expression through miR-203a-3p upregulation, effectively inhibiting HG-induced cellular proliferation, reducing the release of IL-6, IL-1β, and TNF-α, and decreasing the expression of Col-I/IV, thereby alleviating cellular injury. Conversely, SOX6 overexpression and miR-203a-3p inhibition reversed the suppressive effects of KO-circ_0123996 on cell proliferation, inflammation, and fibrosis (102). These findings collectively demonstrate that circ_0123996 acts as a molecular sponge for miR-203a-3p, thereby derepressing SOX6 expression (102). This regulatory cascade potentiates HG-driven mesangial cell proliferation, inflammatory responses, and fibrotic remodeling, which are pathological processes that ultimately exacerbate DN progression.

Studies have also revealed a significant increase in circ_Luc71 expression in *db/db* mice and in HG-stimulated GMCs, KO-circ_Luc71 reduced blood glucose levels, suppressed mesangial cell proliferation, attenuated inflammatory cytokine release (IL-6 and TNF-α), and diminished ECM accumulation. Notably, miR-205-5p inhibition or Tgfbr1 overexpression reversed the suppressive effects of circ_Luc71 silencing on cell proliferation, inflammatory response, and ECM deposition (103). Mechanistic studies confirmed direct binding between circ_Luc71 and miR-205-5p, with miR-205-5p exerting post-transcriptional repression on Tgfbr1 by targeting its 3′ UTR (103). These findings collectively demonstrate that under hyperglycemic conditions, circ_Luc71 acts as a molecular sponge to sequester miR-205-5p, thereby relieving miR-205-5p-mediated suppression of Tgfbr1. The subsequent Tgfbr1 upregulation drives mesangial cell proliferation, inflammatory cascades, ECM remodeling, and pathological processes that ultimately accelerate DN progression.

Activation of the circRNA–miRNA–mRNA network promoted GMCs proliferation, inflammatory response, and ECM accumulation, accelerating DN progression, as well as reversed these effects (**Figure 4**, **Table 2**). Studies revealed significant downregulation of circ_ITCH in HG-treated GMCs and in the kidneys of STZ-induced diabetic mice, circ_ITCH overexpression markedly reduced blood glucose levels in diabetic mice, suppressed HG-induced MC proliferation and migration, and downregulated the expression of renal dysfunction markers, including BUN, SCr, α-SMA, Col-I, FN, TNF-α, IL-6, and IL-1β. These interventions improve renal function, attenuate glomerular hypertrophy, and mitigate fibrotic and inflammatory pathology (104). Mechanistically, circ_ITCH directly binds to and negatively regulates miR-33a-5p, and its overexpression counteracts circ_ITCH-mediated cytoprotection. Furthermore, miR-33a-5p suppresses SIRT6 expression through direct targeting, and KO-SIRT6 abolishes the therapeutic effects of miR-33a-5p inhibition in RMCs (104).

HG-induced inflammation and OS are the inseparable pathological drivers of DN. Elucidating the mechanisms through which HG-regulated circRNA–miRNA networks modulate mesangial cell injury through the inflammation–OS–fibrosis axis is critical for understanding the progression of DN.

Our investigations revealed significant upregulation of circ_FBXW12 and LIN28B, along with miR-31-5p downregulation in the sera of patients with DN and HG-treated HMCs. Silencing circ_FBXW12 or LIN28B suppressed HG-induced HMC proliferation, attenuated IL-6 and TNF-α release, and reduced ECM marker accumulation (Col-I/IV and TGF-β1). Concurrently, this intervention diminished SOD activity, elevated MDA levels, mitigated OS, and normalized HG-accelerated cell cycle progression (105). miR-31-5p inhibition upregulated LIN28B expression and abrogates the cytoprotective effects of circ_FBXW12 silencing. Conversely, miR-31-5p overexpression suppresses LIN28B levels, whereas LIN28B overexpression neutralizes miR-31-5p-mediated inhibition in HMCs (105). Elevated expression of circ_ACTR2 and HMGA2, along with concomitant suppression of miR-205-5p, was observed in the kidney tissues of patients with DN and HG-treated human renal MCs (HRMCs), circ_ACTR2 showed a negative correlation with miR-205-5p expression, silencing circ_ACTR2 partially restored miR-205-5p levels, thereby inhibiting HG-induced HRMC proliferation, attenuating inflammatory cytokine release (IL-6 and TNF-α), reducing ECM accumulation, and modulating OS markers through increased SOD activity and decreased MDA levels (106). Notably, HMGA2 overexpression and miR-205-5p inhibition reversed the cytoprotective effects of miR-205-5p overexpression and circ_ACTR2 silencing (106). These findings demonstrate that circ_ACTR2 functions as a molecular sponge to sequester miR-205-5p, thereby derepressing its inhibitory effect on HMGA2. The resulting upregulation of HMGA2 exacerbated HG-induced HRMC dysfunction by amplifying the inflammatory and OS pathways, ultimately accelerating DN progression (**Figure 4**, **Table 2**).

Studies have revealed significant upregulation of circ_ADAM9 and circ_HOMER1, along with downregulation of miR-545-3p and miR-137 in the serum from patients with DN and HG-treated HMCs, while negative correlations were observed between miR-545-3p and circ_ADAM9 and between miR-137 and circ_HOMER1. Dual KO-circ_ADAM9 and circ_HOMER1 suppressed HG-induced cellular proliferation and migration; reduced TNF-α, IL-1β, and IL-6 release; downregulated fibrotic markers α-SMA, FN, and Col-I/IV. These interventions concurrently enhanced SOD activity, reduced MDA levels, attenuated OS, and ameliorated HMC injury (107, 108). Mechanistic investigations identified USP15 as a direct target of miR-545-3p, with elevated USP15 expression under HG conditions activating Keap1 and suppressing Nrf2. Notably, miR-545-3p inhibition or USP15 overexpression nullified the protective effects of circ_ADAM9 silencing in HMCs. Similarly, the KO-circ_HOMER1 mediated cytoprotection was reversed by miR-137 inhibition and SOX6 overexpression (107, 108). These findings indicate that circ_ADAM9 upregulates USP15 through the sponge adsorption of miR-545-3p, which in turn inhibits the Keap1/Nrf2 pathway, whereas circ_HOMER1 upregulates SOX6 through the sponge adsorption of miR-137, both of which promote HG-induced OS, inflammation, and ECM deposition in HMCs, providing novel insights into circRNA-mediated DN pathogenesis (**Figure 4**, **Table 2**).

Chen et al. demonstrated significant upregulation of HMGB1, TLR4, and p-NF-κB p65 in HG-treated murine GMCs, mechanistically, HMGB1 activates NF-κB through TLR4 binding, evidenced by enhanced p-NF-κB nuclear translocation and DNA-binding activity, HMGB1 inhibition attenuated HG-induced cellular proliferation, reduced ROS generation, suppressed FN, Col-IV, TNF-α, IL-6, and IL-1β, whilst elevating SOD activity to mitigate OS, ECM accumulation, and inflammatory responses, exogenous HMGB1 exacerbates these pathological changes, an effect reversed by TLR4 inhibition (109). Further investigation revealed the downregulation of miR-205 and upregulation of circ_LRP6 in HG-stimulated GMCs. miR-205 directly binds the 3′ UTR of HMGB1 to suppress its expression, miR-205 overexpression significantly inhibited HG-induced cellular injury, whereas HMGB1 overexpression neutralized this protective effect. Notably, circ_LRP6 silencing reduced HMGB1 expression and ameliorated cellular damage—effects counteracted by miR-205 inhibition (109). These findings delineate a sequential regulatory axis: circ_LRP6 functions as a molecular sponge to sequester miR-205, thereby derepressing HMGB1. The elevated HMGB1 subsequently activates the TLR4/NF-κB signaling cascade, driving DN-associated pathological processes. This mechanistic elucidation provided a novel therapeutic rationale for targeted DN intervention (**Figure 4**, **Table 2**).

Overall, SOX6 is a common target molecule downstream of the circ_0123996/miR-203a-3p and circ_HOMER1/miR-137 axes, whereas miR-205-5p is a common sponge-like target molecule for circ_Luc71 and circ-ACTR2. This indicates that in the ceRNA network, different circRNA–miRNA axes can target and regulate the same target molecules, whereas different lncRNAs can adsorb onto the same miRNAs in a sponge-like manner. However, by contrast, although the circ_0123996/miR-203a-203a-3p/SOX6 and circ_LRP6/miR-205/HMGB1 axes have undergone functional validation, their research has only been conducted *in vitro* experiments and lacks *in vivo* experiments. Therefore, the authenticity of these effects requires further investigation.

### 3.3 ceRNA network regulates the role of renal cells in the progression of DN through the cell death–fibrosis pathway

#### 3.3.1 ceRNA network regulates the role of GMCs in the progression of DN through the cell death–fibrosis pathway

As a pivotal pathological process in DN, fibrosis is a critical therapeutic target for preventing disease progression (110). In the sera of both patients with DN and HG-treated human glomerular MCs (HGMCs), elevated expression of lncRNA-HCP5 and HMGA2 was observed, along with reduced miR-93-5p levels. HCP5 silencing inhibited HG-induced HGMC proliferation as well as downregulated Col-I, Col-IV, FN, TNF-α, IL-6, and IL-1β, while simultaneously upregulating apoptosis indicators cleaved-PARP and cleaved-caspase3. This dual modulation attenuates fibrotic and inflammatory responses while promoting apoptosis (111). Furthermore, HG stimulation markedly enhanced p-AKT and p-mTOR levels, which were reversed by KO-HCP5 treatment. Notably, miR-93-5p inhibition restored p-AKT/p-mTOR expression in HG-challenged HGMCs (111). Mechanistic investigations revealed that miR-93-5p was a direct target of HCP5, with HMGA2 functioning downstream of miR-93-5p. miR-93-5p overexpression significantly suppressed HMGA2 expression, mirroring the anti-fibrotic effects of HCP5 silencing. Conversely, miR-93-5p inhibition or HMGA2 overexpression reverses these protective effects (111).

The activation of lncRNA–miRNA networks exhibits dual regulatory effects on GMCs proliferation, demonstrating both promotive and inhibitory capacities. For instance, studies have revealed significantly lower lncRNA-GAS5 expression in the renal tissues of type 2 diabetic patients with DN than in those without DN, showing negative correlations with DN severity markers (proteinuria and eGFR) (112). In DN rat models and HG-treated GMCs, GAS5 downregulation coincided with pathological manifestations including mesangial hyperplasia, enhanced collagen deposition, and elevated expression of fibrotic/inflammatory mediators (FN, Col-IV, and TGF-β1), GAS5 overexpression upregulated SIRT1 expression as well as normalized cell cycle progression in GMCs, concurrently suppressing apoptotic proteins p53 and p21. Conversely, KO-GAS5 exacerbated these pathological parameters (112). Mechanistic investigations have demonstrated that GAS5 directly sequesters miR-221, which normally suppresses its target gene, SIRT1. miR-221 inhibition reduced MC proliferation and expression of fibrotic markers, which was reversed by SIRT1 silencing (112).

**Figure 5:**
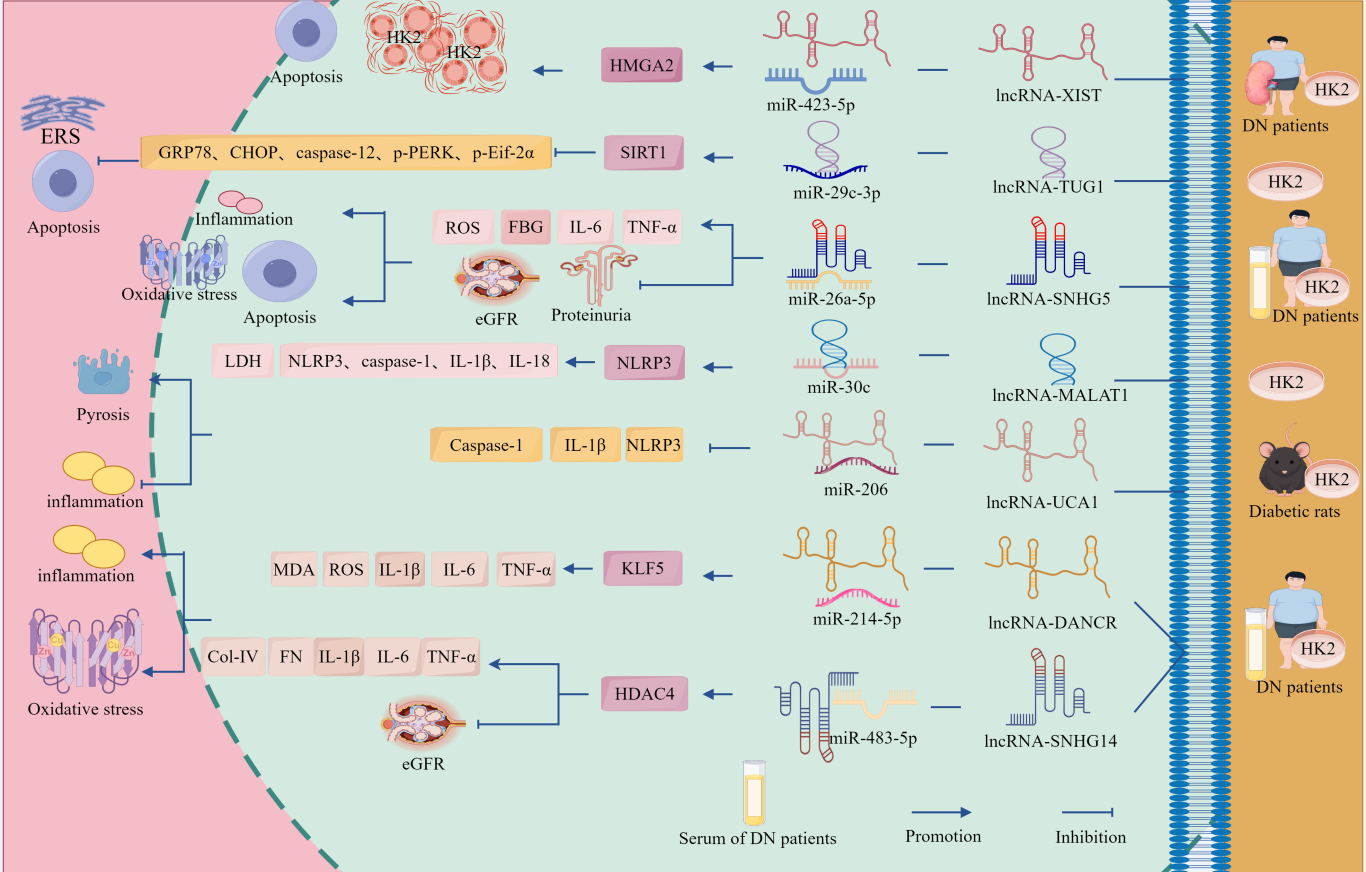
The ceRNA network regulates the role of GMCs in the progression of DN through the cell death-fibrosis pathway. In addition, lncRNA-HCP5 up-regulated HMGA2 expression through sponge adsorption of miR-93-5p, which activated the AKT/mTOR pathway, promoted inflammation, apoptosis and fibrosis, and drove DN progression; whereas lncRNA-GAS5 deregulated the inhibition of SIRT1 through sponge adsorption of miR-221, inhibited the expression of inflammation and fibrosis-related factors and, delayed DN progression. In addition, circ_AKT3 deregulated the inhibition of E-cadherin by miR-296-3p through adsorption of miR-296-3p, which in turn inhibited ECM-associated protein synthesis and apoptosis and suppressed DN progression. And circ_LARP1B, by adsorbing miR-578, deregulated the inhibitory effect of miR-578 on TLR4, which in turn activated the inflammation axis and promoted DN progression. circ_0060077, circ_0068087, circ_TAOK1, circ_0003928, and hsa_circ_0003928 adsorbed miR-145-5p, miR-106a-5p, miR-142-3p, miR-31-5p, and miR-151-3p via sponge, promoted the expression of VASN, ROCK2, SOX6, MAPK6, and Anxa2, which exacerbated HG-induced renal tubular cell injury through the cell death-fibrosis pathway.

To sum up, the lncRNA-HCP5/miR-93-5p/HMGA2 axis promotes DN progression through the activation of the AKT/mTOR pathway, driving inflammatory, apoptotic, and fibrotic cascades. By contrast, the lncRNA-GAS5/miR-221/SIRT1 axis counteracted these pathological processes by suppressing mesangial cell proliferation. This dual regulatory paradigm provides novel strategic insights for precision therapeutics in DN management, highlighting the therapeutic potential of targeting specific lncRNA–miRNA networks based on the disease stage and molecular profiles (**Figure 5**, **Table 3**).

**Table 3:** The ceRNA network regulates the role of renal cells in the progression of DN through the cell death-fibrosis pathway.

Spongy molecule	miRNA	mRNA	Mechanism of action	Pathway	Study subjects	Functional experiments	Result	Refes
lncRNA- HCP5	93-5p	HMGA2	Down-regulated: PARP, caspase-3 Up-regulated: Col-I/IV, FN, TNF-α, IL-6, IL-1β, p-AKT, p-mTOR	Cell death -Fibrosis	Serum from DN patients and HG-treated HGMCs	Gain and loss	Promoted fibrosis, inflammatory response while inhibiting apoptosis	(111)
lncRNA- GAS5	221	SIRT1	Down-regulated: FN, Col-IV, TGF-β1, p53, p21		Kidney tissue of DN patients, DN rats and HG-treated MCs	Gain and loss	Reduced fibrosis, inflammatory response, inhibited apoptosis	(112)
circ_ AKT3	296-3p	E-cadherin	Down-regulated: caspase-3, Bax/Bcl-2, FN, Col-I, Col-IV		*db/db* mouse kidney tissue and HG-treated GMCs	Gain	Reduced HG-induced apoptosis and fibrosis	(17)
circ_ LARP1B	578	TLR4	Up-regulated: IL-6, TNF-α, IL-1β, IL-18, NLRP3, caspase-1		Blood cells from DN patients and HG-induced MCs	Gain and loss	Exacerbated pyroptosis, inflammation and cell proliferation	(113)
circ_ 0060077	145-5p	VASN	Down-regulated: Bcl-2, SOD Up-regulated: Bax, caspase-3, ROS, MDA, IL-6, TNF-α, IL-1β, FN, Col-I/IV		Serum from DN patients and HG-treated HK-2 cells	Gain and loss	Inhibition of apoptosis, attenuation of OS and inflammatory responses, and alleviation of fibrosis	(114)
circ_ 0068087	106a-5p	ROCK2						(115)
circ_ TAOK1	142-3p	SOX6						(116)
circ_ 0003928	31-5p	MAPK6	Down-regulated: Bcl-2, SOD Up-regulated: Bax, TNF-α, IL-6, IL-1β, ROS, FN, TGF-β1, MDA		DN patients and HG-treated HK-2 cells	Gain and loss	Promoted apoptosis, OS, inflammatory response and fibrosis	(117)
hsa_circ _0003928	151-3p	Anxa2	Down-regulated: Bcl-2, SOD Up-regulated: Bax, TNF-α, IL-6, IL-1β, ROS, FN, TGF-β1, MDA, caspase-3					(118)

Emerging evidence has established apoptosis and inflammation as central pathogenic mechanisms in DN that play pivotal roles in disease acceleration and clinical deterioration. Recent advances have highlighted the ubiquitous involvement of ncRNAs in virtually all pathophysiological processes, including apoptotic regulation and cellular proliferation, that are directly relevant to DN progression (119, 120). Studies have revealed downregulation of circ_AKT3 and E-cadherin in the renal tissues of *db/db* mice and HG-treated GMCs, concomitant with miR-296-3p upregulation. A positive correlation was observed between circ_AKT3 and E-cadherin, whereas a negative correlation was observed between circ_AKT3 and miR-296-3p. Circ_AKT3 overexpression reduces the protein expression of caspase-3 and the Bax/Bcl-2 ratio, attenuates HG-induced apoptosis, and suppresses fibrotic protein accumulation in FN, Col-I, and Col-IV (17). Mechanistic studies showed that circ_AKT3 directly binds to miR-296-3p through complementary sequences, thereby effectively sequestering its activity. miR-296-3p in turn suppresses E-cadherin expression by targeting its 3′ UTR; therefore, E-cadherin overexpression reversed miR-296-3p-mediated enhancement of ECM deposition (17).

Distinct from apoptosis, pyroptosis is a novel pro-inflammatory apoptotic mechanism mediated by caspase-1 activation following NLRP3 inflammasome assembly, driving pyroptotic execution and inflammatory cytokine release (121, 122). Emerging evidence has implicated aberrant pyroptosis in DN pathogenesis, with ncRNAs critically regulating NLRP3 activation and pyroptosis pathways (4). For instance, studies identified significant upregulation of circ_LARP1B and TLR4 alongside downregulation of miR-578 in sera from patients with DN and in HG-stimulated renal GMCs. Overexpression of circ_LARP1B suppressed cellular proliferation, induced G0–G1 phase cell cycle arrest, and upregulated the expression of IL-6, TNF-α, IL-1β, and IL-18, as well as the pyroptosis-associated markers NLRP3 and caspase-1, thereby exacerbating pyroptosis and inflammatory pathology. Conversely, circ_LARP1B silencing promotes S-phase progression, attenuates pyroptotic cell death, and reduces inflammatory cytokine secretion (113). Mechanistically, TLR4 activation stimulated NLRP3 inflammasome formation and caspase-1 expression, thereby amplifying pyroptotic signaling. miR-578 directly suppresses TLR4 expression, with TLR4 overexpression negating the anti-inflammatory and anti-pyroptotic effects of miR-578; therefore, circ_LARP1B functions as a molecular sponge sequestering miR-578, thereby relieving miR-578-mediated TLR4 suppression. This cascade activates the TLR4/NLRP3/caspase-1 axis, ultimately accelerating the progression (113).

Collectively, circ_AKT3 deregulated the inhibition of E-cadherin by miR-296-3p through the adsorption of miR-296-3p, reducing the rate of HG-induced apoptosis and fibrosis-associated protein expression, whereas circ_LARP1B deregulated the inhibitory effect of miR-578 on TLR4 through the adsorption of miR-578, which in turn activated the TLR4/NLRP3/caspase-1 axis, promoting DN progression. The circRNA–miRNA–mRNA network plays a critical role in HG-induced apoptosis, pyroptosis, and fibrosis in renal GMCs (**Figure 5**, **Table 3**).

#### 3.3.2 ceRNA network regulates the role of TECs in the progression of DN through the cell death–fibrosis pathway

Emerging evidence has identified significant upregulation of circ_0060077, circ_0068087, circ_TAOK1, VASN, ROCK2, and SOX6 in peripheral blood samples from patients with DN and HG-treated HK-2 cells. KO-circ_0060077, circ_0068087, or circ_TAOK1 consistently showed therapeutic efficacy by enhancing cellular proliferation; downregulating pro-apoptotic markers (Bax and cleaved caspase-3); reducing ROS, MDA, IL-6, TNF-α, IL-1β, FN, Col-I, and Col-IV; elevating anti-apoptotic Bcl-2 expression and SOD activity; and concurrently attenuating apoptosis, OS, inflammation, and fibrosis, thereby collectively ameliorating HG-induced HK-2 cell injury (114–116). Furthermore, miR-145-5p, miR-106a-5p, and miR-142-3p inhibited VASN, ROCK2, and SOX6 expression by binding to their respective 3′ UTRs. Inhibition of these miRNAs or overexpression of VASN, ROCK2, and SOX6 reversed the protective effects of circ_0060077, circ_0068087, and KO-circ_TAOK1 (114–116).

Emerging studies have identified concomitant upregulation of circ_0003928, hsa_circ_0003928, MAPK6, and Anxa2 with downregulation of miR-31-5p and miR-151-3p in both patients with DN and HG-treated HK-2 cells, whereas KO-circ_0003928 and hsa_circ_0003928 upregulated miR-31-5p and miR-151-3p expression, respectively, both increased Bcl-2 expression and decreased Bax, TNF-α, IL-6, IL-1β, and ROS expression (117, 118). In addition, KO-circ_0003928 also downregulated FN, TGF-β1, and MDA expression and increased SOD activity (117), whereas KO-hsa_circ_0003928 decreased HG-induced cell viability and downregulated cleaved-caspase-3 expression (118), but both KOs reduced apoptosis, inhibited OS, and inflammatory responses (117, 118). Furthermore, circ_0003928 directly targeted miR-31-5p, and miR-31-5p overexpression inhibited the AKT signaling pathway by inhibiting MAPK6 and thus the AKT signaling pathway; overexpression of MAPK6 in turn reversed the protective effect of miR-31-5p (117), whereas inhibition of miR-151-3p reversed the KO-hsa_circ_0003928 effect, and overexpression of miR-151-3p significantly inhibited mRNA and protein expression of Anxa2 (118).

As shown, circ_0060077, circ_0068087, and circ_TAOK1 promoted the expression of VASN, ROCK2, and SOX6 by sequestering miR-145-5p, miR-106a-5p, and miR-142-3p, respectively, thereby exacerbating HG-induced renal tubular cell injury. Circ_0003928 deregulated the inhibition of MAPK6 by the adsorption of miR-31-5p, which in turn activated the AKT signaling pathway, whereas hsa_circ_0003928 deregulated the inhibition of Anxa2 by the adsorption of miR-151-3p, which led to HG-induced apoptosis, fibrosis, inflammation, and OS (**Figure 5**, **Table 3**).

In summary, cell death (such as apoptosis and pyroptosis) is a key pathological factor leading to podocyte damage, whereas fibrosis caused by inflammation and OS is the core driving factor of mesangial cell damage. Furthermore, the pathological changes caused by inflammation, OS, apoptosis, and fibrosis are the key factors in tubular epithelial cell damage, indicating that the terminal pathology of renal cells (podocytes, GMCs, and TECs) are closely coupled, and the key to regulating DN progression is to block this pathological process (such as inflammation and apoptosis/fibrosis). The ceRNA network regulates DN progression by acting on its key downstream molecules to activate or inhibit inflammation, OS, the inflammation–OS axis, cell death, and fibrosis processes, suggesting that the key molecules downstream of the ceRNA network and the dynamic balance within the network are central to DN progression. Therefore, research on target molecules and dynamic balance within the network should be prioritized for disease prevention and treatment. In addition, different ceRNA networks can target and regulate downstream target molecules. Different lncRNAs can adsorb the same miRNAs in a sponge-like manner, whereas the same lncRNA can adsorb different miRNAs. Therefore, it is questionable whether different circRNAs can adsorb the same miRNA in a sponge-like manner, whether different miRNAs can be adsorbed by the same circRNA to exert their functions, or whether this regulatory network plays a role in diseases with similar pathological processes.

## 4 Extracellular ncRNAs interfere with DN progression

Exosomes carry biomolecules such as proteins, lipids, DNA, mRNA, miRNA, lncRNA, and circRNA, mediate intercellular communication, and participate in physiological and pathological processes such as tissue repair (123, 124). ncRNAs play important roles in many molecular processes in DN, such as gene expression, differentiation, cell cycle regulation, and immune responses (125). With the continuous advancement of exosome-related research, exosome ncRNAs can stably exist in human body fluids (such as serum and urine) under the protection of vesicle structures (126). Therefore, investigating serum- and urinary exosome-derived ncRNAs may represent a critical avenue for the prevention and management of DN.

### 4.1 Exosomes interfere with DN progression through miRNA

miRNAs are more stable, more specific, and less susceptible to degradation than lncRNAs and mRNAs in tissues, urine, and blood and can be encapsulated in exosomes, which carry and release them into target cells or tissues to exert their effects (127). However, compared to free miRNAs in urine, miRNAs produced by urinary exosomes (UExos) are resistant to endogenous RNase activity, highly stable, and cannot be confused with miRNAs that pass through the glomerular filtration barrier (128). Therefore, UExos miRNAs may be more accurate diagnostic indicators of the disease.

In recent years, increasing evidence has suggested that urinary exosomes miRNAs are involved in the development and progression of DN. For example, Wang et al. found that the expression of miRNA-615-3p was upregulated in the urine exosomes of patients with T2DM and DN, and the expression of miRNA-615-3p in the urine exosomes of DN patients was more significant (129). Additionally, clinically relevant results showed that the expression levels of urinary exosome miRNA-615-3p were positively correlated with serum creatinine (Scr), urea, protein-to-creatinine ratio (PCR), 24-hour urine protein, cystatin C, and TGF-β1, and negatively correlated with eGFR and albumin (129), it shows that miRNA-615-3p in urine exosomes is a key molecule regulating DN.

**Figure 6:**
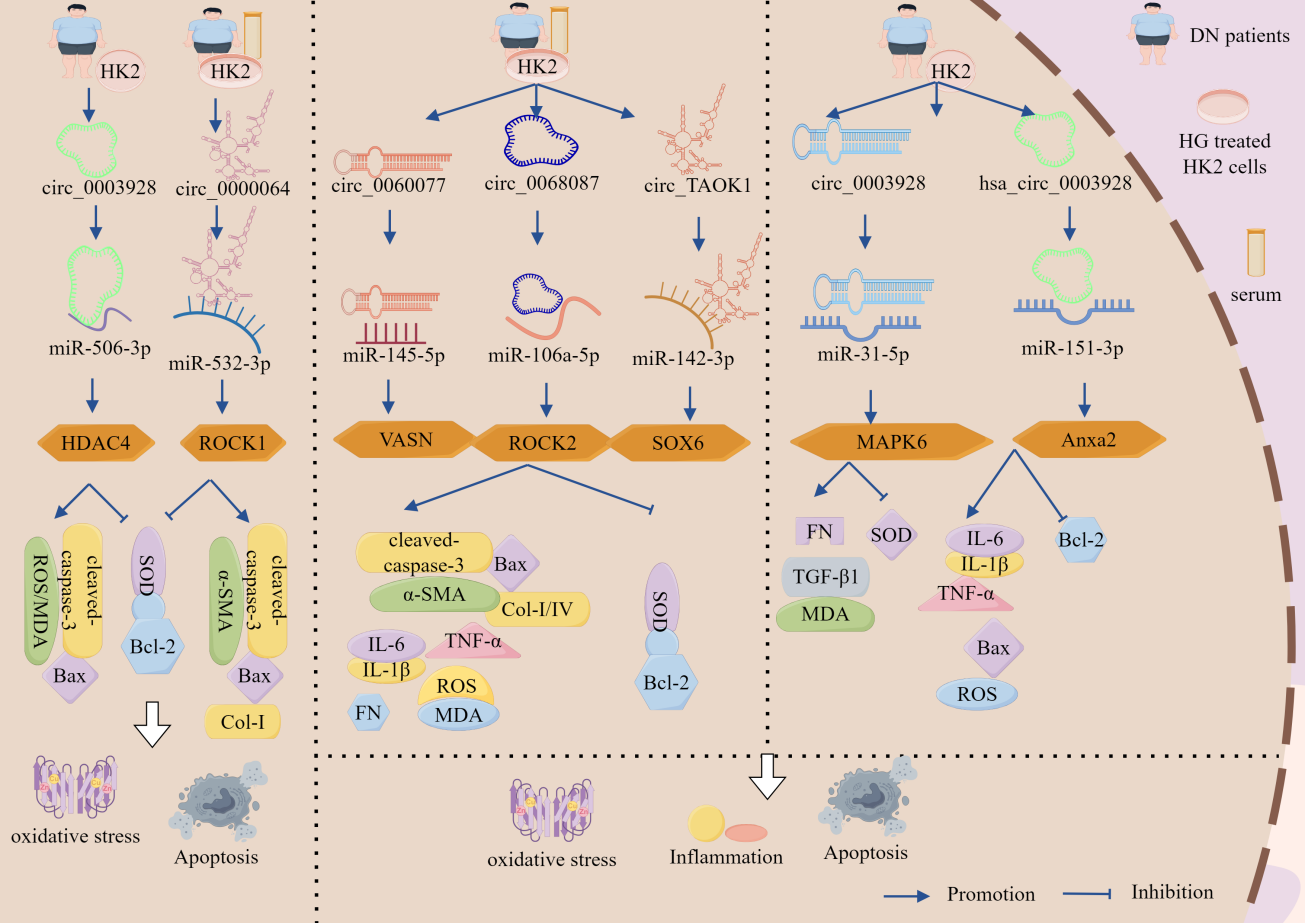
Exosomes interfere with DN progression through miRNA intervention. In DN patients, urinary exosome-derived miR-615-3p and miR-516b-5p are significantly upregulated.miR-615-3p expression positively correlates with Scr, urea, PCR, 24-hour urinary protein, cystatin C, and TGF-β1 levels, while negatively correlating with eGFR and albumin. miR-516b-5p upregulation significantly increases IL-18, IL-1β, caspase-1, and NLRP3 activity while inactivating the SIRT3/AMPK signaling pathway. SIRT3 is a direct target of miR-516b-5p, and either SIRT3 overexpression or miR-516b-5p silencing reverses these pathological effects. Additionally, urinary exosomal miR-145-5p in DN patients promotes podocyte apoptosis by suppressing Srgap2 and subsequently activating the RhoA/ROCK pathway. This effect is reversed by either miR-145-5p inhibitors or Srgap2 overexpression.

Zhang et al. showed *in vitro* experiments that miR-516b-5p derived from urinary exosomes of DN patients was significantly upregulated, with increased expression of IL-18 and IL-1β, as well as enhanced activity of Caspase-1 and NLRP3, the SIRT3/AMPK signaling pathway was inactivated; however, these effects were partially reversed by silencing miR-516b-5p (130). Furthermore, SIRT3 was identified as a target gene of miR-516b-5p, and SIRT3 overexpression reversed the effects of DN-Exo and miR-516b-5p mimics (130). This indicates that urinary exosome miR-516b-5p promotes inflammatory responses and activates NLRP3 inflammasomes through the SIRT3/AMPK pathway, playing a key role in DN, the study also found that urinary exosomes containing miR-145-5p in patients with DN promote podocyte apoptosis by inhibiting Srgap2 and subsequently activating the RhoA/ROCK signaling pathway, however, the presence of miR-145-5p inhibitors or Srgap2 overexpression partially reversed this effect (130). This suggests that exosomal miR-145-5p may play a role in the pathological process of DN. This suggests that urinary extracellular miRNA-615-3p, miR-516b-5p, and miR-145-5p may serve as novel non-invasive biomarkers for assessing DN progression, enabling early intervention to improve clinical management (**Figure 6**).

In addition to urine exosomes containing miRNAs, blood also contained miRNAs (**Figure 6**). miR-4449 is a carrier of exosomes in the serum of patients with DN, and its levels increase with the progression of DN. In addition, DN exosomes and miR-4449 treatment promote the secretion of IL-1β and IL-18 by renal cells, ROS accumulation, and pyroptosis, as well as significantly increase the expression of HIC-1, GSDMD-N, and NLRP3 (131). However, in the presence of a miR-4449 inhibitor, IL-1β and IL-18 section significantly reduced in DN-conditioned cell culture and pyroptosis in DN-conditioned cells was inhibited (131). This indicated that DN exosomes promote pyroptosis in HK-2 cells by transmitting miR-4449, which plays a key role in the pathogenesis of DN. In addition, studies by Wang et al. showed that plasma exosomes miR-320a and miR-27a are closely associated with metabolic syndrome and T2DM and are also closely related to the development of insulin resistance (132). Backes et al. revealed that compared to DM patients without DN, DM patients with DN had elevated levels of plasma exosomal miR-21 and miR-126 (133). This suggests that blood exosomal miR-4449, miR-320a, miR-27a, miR-21, and miR-126 are promising biomarkers for the diagnosis of DN.

### 4.2 Exosomes intervene in DN progression through the circRNA–miRNA–mRNA network

Fibrosis, apoptosis, inflammation, and OS are the key drivers of DN development. Renal cells, including mesangial and tubular epithelial cells, are important targets for DN (4–6). Therefore, regulation of these drivers and target tissues is crucial for the prevention and treatment of DN. Exosomes are important carriers of circRNAs. By forming a ‘transport carrier-functional molecule’ synergistic system with circRNA–miRNA–mRNA, exosomes can regulate fibrosis, apoptosis, inflammation, and OS, thereby participating in the progression of DN (134, 135). Therefore, investigating the exosomal circRNA–miRNA–mRNA network that can block fibrosis, apoptosis, inflammation, and OS to alleviate renal cell damage is a key approach for preventing and treating DN.

#### 4.2.1 Exosomes regulate fibrosis through the circRNA–miRNA–mRNA network to intervene in DN progression

Clinical studies have identified significant up-regulation of circ_0125310 in both serum and renal tissues of DN patients, demonstrating a positive correlation with proteinuria severity (136). *In vitro* studies revealed that HG-stimulated GMCs secrete exosomes enriched with circ_0125310, which undergo efficient cellular internalization, Notably, exposure to HG-conditioned GMCs exosomes induced: increased EdU-positive cell proportion; elevated G2/M phase cell population; up-regulated fibrotic markers p-cadherin, ZO-1, crucially, KO-circ_0125310 reversed these pro-fibrotic effects, confirming exosomal circ_0125310 role in promoting MC proliferation and ECM remodeling (136). Furthermore, circ_0125310 directly binds to miR-422a and inhibits its expression, miR-422a targets the 3’ UTR of IGF1R and inhibits its expression, and overexpression of IGF1R activates the downstream p38 signaling pathway, in addition, circ_0125310 overexpression in turn upregulates the miR-422a-dependent IGF1R/p38 axis, promoting GMCs proliferation and fibrosis (136). Suggests that HG-induced exosomes from GMCs promote MCs proliferation and fibrosis by delivering circ_0125310, adsorbing miR-422a, deregulating its inhibition of IGF1R, and activating the p38 signaling pathway.

Li et al. also found that circ_TAOK1 was greatly elevated in exosomes secreted by HG-treated GMCs and serum exosomes from DN patients, with significant up-regulation of the levels of PCNA, cytokinin D1, α-SMA, FN, N-cadherin, and SMAD3 and significant down-regulation of the level of E-cadherin, which promoted the proliferation, fibrosis, accelerated fibrosis and EMT, while KO-circTAOK1 reversed the above effects, suggesting that exosomal circ_TAOK1 promotes GMC injury (137). Furthermore, it was found that circ_TAOK1 directly binds to miR-520h and represses its expression, which in turn targets the 3’ UTR of SMAD3 and represses its transcription, and furthermore, KO-miR-520h or overexpression of SMAD3 in turn reverses the protective effect of KO-circTAOK1 on GMC (137). From the above, it can be seen that exosomal circ_TAOK1 delivery to GMC, by adsorbing miR-520h, deregulates its inhibition of SMAD3, which in turn promotes GMC proliferation, fibrosis and EMT.

It was also found that miR-143 expression was greatly down-regulated in HG-treated GMCs, DN patient serum and DN rat kidneys, exosomal circ_DLGAP4 expression was significantly elevated and positively correlated with UAER and eGFR, overexpression of circ_DLGAP4 up-regulated EdU positivity, and significantly promoted the proliferation of GMCs, G2/M phase of the cell cycle block, and fibrosis markers p-cadherin, Mcp-1 expression, and promoted GMCs injury, while KO-circ_DLGAP4 reversed the above phenotypes (138). It was also found that miR-143 is a direct target of circ_DLGAP4 and ERBB3 is a downstream target gene of miR-143. miR-143 inhibits the expression of ERBB3 by binding to its 3’ UTR, whereas circ_DLGAP4 up-regulates ERBB3 through the inhibition of miR-143, which in turn activates the NF-κB pathway and induces MMP-2 expression, exacerbating fibrosis (138). It suggests that exosomal circ_DLGAP4 exacerbates DN progression by adsorbing miR-143 to deregulate ERBB3 and activating the NF-κB/MMP-2 pathway to promote thylakoid proliferation and fibrosis.

**Figure 7:**
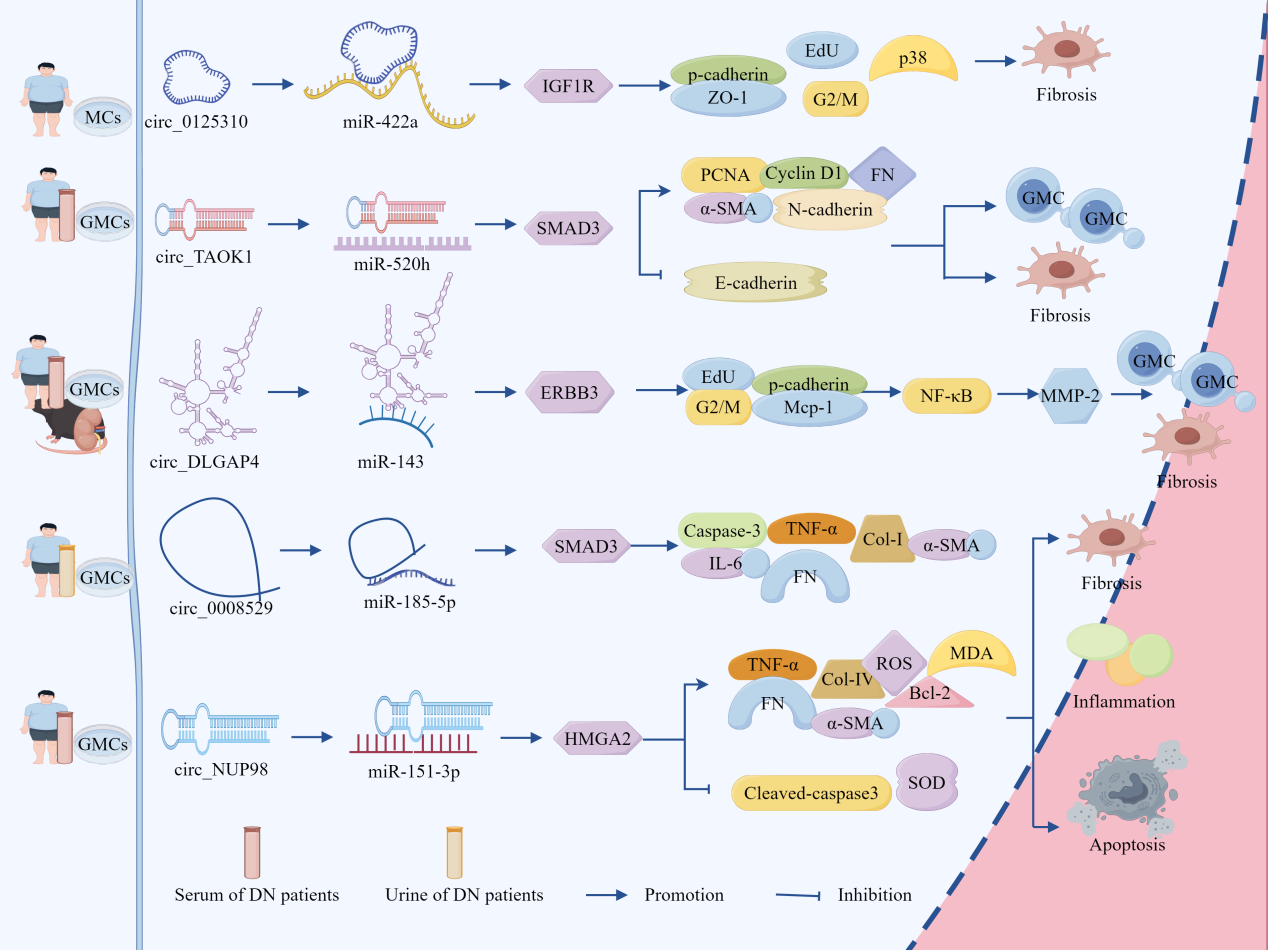
Exosomes intervene in DN progression through the circRNA-miRNA-mRNA network. Exosomes circ_0125310, circ_TAOK1 and circ_DLGAP4 up-regulated IGF1R, SMAD3 and expressed ERBB3 through sponge adsorption of miR-422a, miR-520h and miR-143, respectively, where activation of the circ_0125310/miR-422a/IGF1R axis in turn up-regulated its downstream p38 signaling pathway, whereas activation of the circ_DLGAP4/miR-143/ERBB3 axis up-regulated the NF-κB/MMP-2 pathway, both of which promoted fibrosis and GMCs proliferation via the circRNA-miRNA-mRNA network, thereby exacerbating DN progression. In addition, exosomes circ_0008529 and circ_NUP98 up-regulated SMAD2 and HMGA2 through sponge adsorption of miR-185-5p and miR-151-3p, respectively, which promoted cell proliferation, fibrosis, and inflammation and accelerated DN progression.

Similarly, exosomes circ_0125310, circ_TAOK1 and circ_DLGAP4 up-regulated IGF1R, SMAD3 and expressed ERBB3 through sponge adsorption of miR-422a, miR-520h and miR-143, respectively, where circ_0125310/miR-422a/IGF1R axis activation in turn up-regulated its downstream p38 signaling pathway, whereas activation of the circ_DLGAP4/miR-143/ERBB3 axis up-regulated the NF-κB/MMP-2 pathway, both of which contributed to fibrosis via the circRNA-miRNA-mRNA network, thereby exacerbating DN progression (**Figure 7**, **Table 4**).

**Table 4:** Exosomes intervene in DN progression through the circRNA-miRNA-mRNA network.

Spongy molecule	miRNA	mRNA	Mechanism of action	Pathway	Study subjects	Functional experiments	Result	Refes
circ_ 0125310	422a	IGF1R	Up-regulated: EdU, G2/M, p-cadherin, ZO-1, p38	Fibrosis	HG-induced MCs exosomes	Gain	Promoted proliferation and fibrosis of GMCs	(136)
circ_ TAOK1	520h	SMAD3	Down-regulated: E-cadherin Up-regulated: PCNA, CCND1, α-SMA, FN, N-cadherin, SMAD3		Exosomes secreted by GMC treated with HG and exosomes in serum from DN patients	Gain and loss	Promoted fibrosis and EMT	(137)
circ_ DLGAP4	143	ERBB3	Up-regulated: EdU, G2/M, p-cadherin, Mcp-1, NF-κB, MMP-2		HG-treated MCs, serum from DN patients, and kidneys from DN rats	Gain and loss	Promoted proliferation and fibrosis of GMCs	(138)
circ_ 0008529	185-5p	SMAD2	Up-regulated: caspase-3, TNF-α, IL-6, FN, Col-I, α-SMA	Apoptosis, inflammation, and fibrosis	HG-stimulated HK-2 cells and exosomes from the urine of DN patients	Gain	Promoted apoptosis, inflammation and fibrosis in renal tubular cells	(139)
circ_ NUP98	151-3p	HMGA2	Down-regulated: caspase-3, SOD Up-regulated: FN, Col-IV, Bcl-2, TNF-α, IL-6, IL-1β, ROS, MDA		DN patients’ serum and HG-stimulated GMCs	Gain and loss	Inhibition fibrosis, inflammatory response and OS, promoted apoptosis	(140)

#### 4.2.2 Exosomes regulate apoptosis, inflammation, and fibrosis through the circRNA–miRNA–mRNA network to intervene in DN progression

In HG-stimulated HK-2 cells and urinary exosomes from DN patients, circ_0008529 and SMAD2 expression were significantly up-regulated, and miR-185-5p expression was down-regulated. KO-circ_0008529 increased cell viability, inhibited caspase-3 activation, and reduced cell cycle arrest, TNF-α, IL -6 release and FN, Col-I, and α-SMA accumulation (139). In addition, inhibition of miR-185-5p or overexpression of SMAD2 up-regulated circ_0008529 expression, reversed the protective effect of KO-circ_0008529 on HK-2 cells, and exacerbated cellular injury, whereas circ_0008529 directly bound miR-185-5p and sponged to deregulate the effect of miR-185-5p on the SMAD2 inhibition (139). It is shown that exosome circ_0008529 upregulates SMAD2 expression through adsorption of miR-185-5p, promotes renal tubular cell apoptosis, inflammation and fibrosis, and exacerbates DN progression.

It was also found that exosomal circ_NUP98 expression was significantly elevated and miR-151-3p expression was marked reduction in serum and HG-stimulated GMCs from DN patients, KO-circ_NUP98 inhibited HG-induced cell proliferation, down-regulated FN, Col-IV, Bcl-2, TNF-α, IL-6, IL-1β, ROS and MDA, up-regulated cleaved-caspase3, SOD, inhibited fibrosis, inflammatory response and OS, promoted apoptosis, and attenuated HG-induced injury in HMCs, whereas inhibition of miR-151-3p or overexpression of HMGA2 both reversed the protective effect of KO-circ_NUP98 on HMCs (140). Furthermore, it was found that miR-151-3p directly targets HMGA2, whereas circ_NUP98 can upregulate HMGA2 expression by inhibiting miR-151-3p, and HMGA2 overexpression reverses the protective effect of miR-151-3p, revealing that circ_NUP98 upregulates HMGA2 expression by adsorbing miR-151-3p that promotes HG-induced proliferation, fibrosis and inflammation and OS in HMCs (140).

Ultimately, targeted inhibition of the circ_0008529/miR-185-5p/SMAD2 and circ_NUP98/miR-151-3p/HMGA2 axis is one of the pathways to inhibit HG-induced cell proliferation, fibrosis, and inflammation, which helps to provide a new perspective for the mechanistic study and treatment of DN (**Figure 7**, **Table 4**).

### 4.3 Stem cell exosomes intervene in DN progression through ncRNA networks

Stem cells have unlimited or immortal self-renewal capabilities and can produce at least one highly differentiated offspring (141). Stem cell-derived exosomes encapsulate the activity of parent cells and are smaller and simpler than the cells themselves (142). Therefore, stem cell-derived exosomes have become a popular topic in medical research in recent years.

Several studies have shown that stem cell-derived exosomal ncRNAs participate in the pathophysiology of DN. For example, in a DN rat model, circ_ATG7 derived from urinary stem cells (USCs) was significantly downregulated, overexpression of circ_ATG7 significantly alleviated kidney damage in DN rats; however, after knocking out circ_ATG7 derived from USCs, the improvement in kidney function and therapeutic effect in DN rats were lost (143). circ_ATG7 can target miR-4500 and regulate the SOCS1/STAT3 signaling pathway through miR-4500, thereby inhibiting the release of inflammatory factors and promoting the conversion of macrophage phenotype from M1 to M2, thus inhibiting DN progression (143). In addition, human urine-derived stem cells (hUSCs) have been shown to have great potential in the treatment of DM (144). Under HG conditions, exo-miR-16–5p released by hUSCs alleviates foot cell apoptosis in rats with DN and improves foot cell proliferation by inhibiting VEGFA expression (145). Furthermore, Zhong et al. found that miR-451a in hUCMSCs-Exo not only enhances the viability of HK-2 cells from DN mice and reduces their damage, but also downregulates α-SMA and upregulates E-cadherin expression, thereby helping to restart the blocked cell cycle, miR-451a can also inhibit the expression of P15 and P19 by targeting their 3′UTR regions, and reverse EMT both *in vivo* and *in vitro*, thereby inhibiting renal fibrosis (146). In addition, The study also found that miR-451a in hUCMSCs-Exo can reduce renal fibrosis by downregulating p15INK4b and p19INK4d, significantly improving renal pathological changes (146) (**Table 5**).

**Table 5:** The regulatory role of stem cell-derived exosomes and their ncRNA in DN.

Exosome origin	Carried ncRNA	Mechanism of action	Effects	Model	Refs
USC	Exo-circ_ ATG7	USC-derived exosomes Circ_ATG7 regulate the SOCS1/STAT3 signaling pathway by targeting miR-4500, thereby inhibiting the release of inflammatory factors and promoting the conversion of macrophage phenotype from M1 to M2.	Reduced renal damage in DN rats and inhibited DN progression	DN rats	(143)
hUSCs	Exo-miR- 16-5p	Exo-miR-16–5p released by hUSCs suppresses VEGFA expression.	Reduce podocytes apoptosis and promote podocytes proliferation	DN rats	(145)
	Exo-miR- 451a	Exo-miR-451a not only downregulates α-SMA, p15INK4b, and p19INK4d and upregulates E-cadherin expression, but also targets the 3′UTR regions of P15 and P19 to inhibit their expression, thereby reversing EMT *in vivo* and *in vitro*.	Reduce renal fibrosis and significantly improve renal pathological changes.	DN mice and HK-2 cells	(146)
ADSCs	Exo-miR- 26a-5p	Exo-miR-26a-5p inactivated the NF-κB pathway and downregulated VEGFA expression by targeting TLR4.	Protecting HG-induced MP5 cells from damage and exerting a protective effect on DN	MP5 cells	(147)
	Exo-miR- 251-5p	Exo-miR-251-5p inhibits transcription factor ZEB2	Inhibition of HG-induced EMT	podocytes	(148)
	Exo-miR- 125a	Exo-miR-125a directly acts on histone deacetylase 1, activates endothelin-1, and reduces blood glucose levels, serum creatinine, and 24-hour proteinuria in DN rats.	Inhibition of mesangial proliferation and renal fibrosis	DN rats	(149)
BMSCs	Exo-miR- 30e-5p	Exo-miR-30e-5p targets ELAVL1 and inhibits ELAVL1 expression.	Inhibited ferroptosis in HK-2 cells	HK-2 cell	(150)
hP-MSCs	Exo-miR-99b-5p	hP-MSCs-Exo-miR-99b-5p increases autophagy activity and reduces GMC proliferation and ECM accumulation by targeting and inhibiting mTOR signaling.	Reduced renal fibrosis in DN and improved renal function	DM rats, MCs cells	(151)

This study also showed that adipose-derived mesenchymal stem cells (ADSCs) intervene in DN progression by secreting ncRNA exosomes. Peng et al. reported that Exo-miR-125a secreted by ADSCs could improve damaged mesangial cells (152). Exo-miR-26a-5p derived from ADSCs protects HG-induced MP5 cells from damage by targeting TLR4, inactivating the NF-κB pathway, and downregulating vascular endothelial growth factor A, thereby exerting a protective effect on DN (147). Additionally, ADSC-Exos can carry miR-251-5p into podocytes, potentially inhibiting ZEB2 transcriptional repression and thereby suppressing HG-induced EMT (148). In addition, Hao revealed that ADSCs Exos, by carrying miR-125a, can directly act on histone deacetylase 1, thereby regulating the activation of endothelin-1, leading to a decrease in blood glucose levels, serum creatinine, and 24-hour urine protein excretion in DN rats and inhibition of mesangial proliferation and renal fibrosis (149). These studies suggest that the intervention effect of ADSCs-Exo on DN progression may be closely related to the miRNAs they carry, providing multiple targets and pathways for the treatment of DN (**Table 5**).

In addition, MSC-Exos exert a significant regulatory effect on DN progression by promoting tissue repair, inhibiting fibrosis, and reducing inflammation. BMSC-Exos, another type of MSC-Exos, also demonstrated regulatory potential in DN progression. For example, depletion of miR-30e-5p derived from BMSC-Exos promotes ferroptosis in HK-2 cells, and ELAVL1 is a target of miR-30e-5p. Overexpression of miR-30e-5p or KO-ELAVL1 reversed the effects of BMSC-Exos on HK-2 cells, indicating that inhibiting BMSCs-Exo-miR-30e-5p is one of the pathways for preventing and treating DN (150). In addition, human placental mesenchymal stem cells (hP-MSCs) also play an important regulatory role in DN. Li et al. found that hP-MSCs-Exo-miR-99b-5p target and inhibit mTOR signaling, thereby increasing autophagy activity and reducing proliferation and ECM accumulation in GMCs of diabetic mice and HG-treated mice, significantly alleviating renal fibrosis in DN and improving renal function (151) (**Table 5**).

As a whole, exosomes provide the possibility of cell-free therapy, thereby minimizing the safety issues associated with live cell administration. By contrast, stem cell-derived exosomes protect primary victims of DN (such as podocytes, TECs, and GMCs). Therefore, stem cell-derived exosomes can be considered a potential therapeutic approach for DN and hold great promise for DN treatment.

## 5 Targeted regulation of ncRNA as intervention in DN progression

To date, no specific treatment exists for DN. Traditional treatments include controlling blood sugar and blood pressure, limiting protein intake, reducing fat intake, lowering blood sugar and blood pressure, and maintaining water, electrolyte, and acid–base balance (25, 26). These treatments are ineffective in preventing the progression of DN and increase the risk of adverse reactions such as acute kidney injury and hyperkalaemia (153). Therefore, new therapies are urgently needed to improve kidney function in DN and reduce its progression rate.

### 5.1 Biomolecule-targeted regulation of the ceRNA network intervenes in DN progression

**Figure 8:**
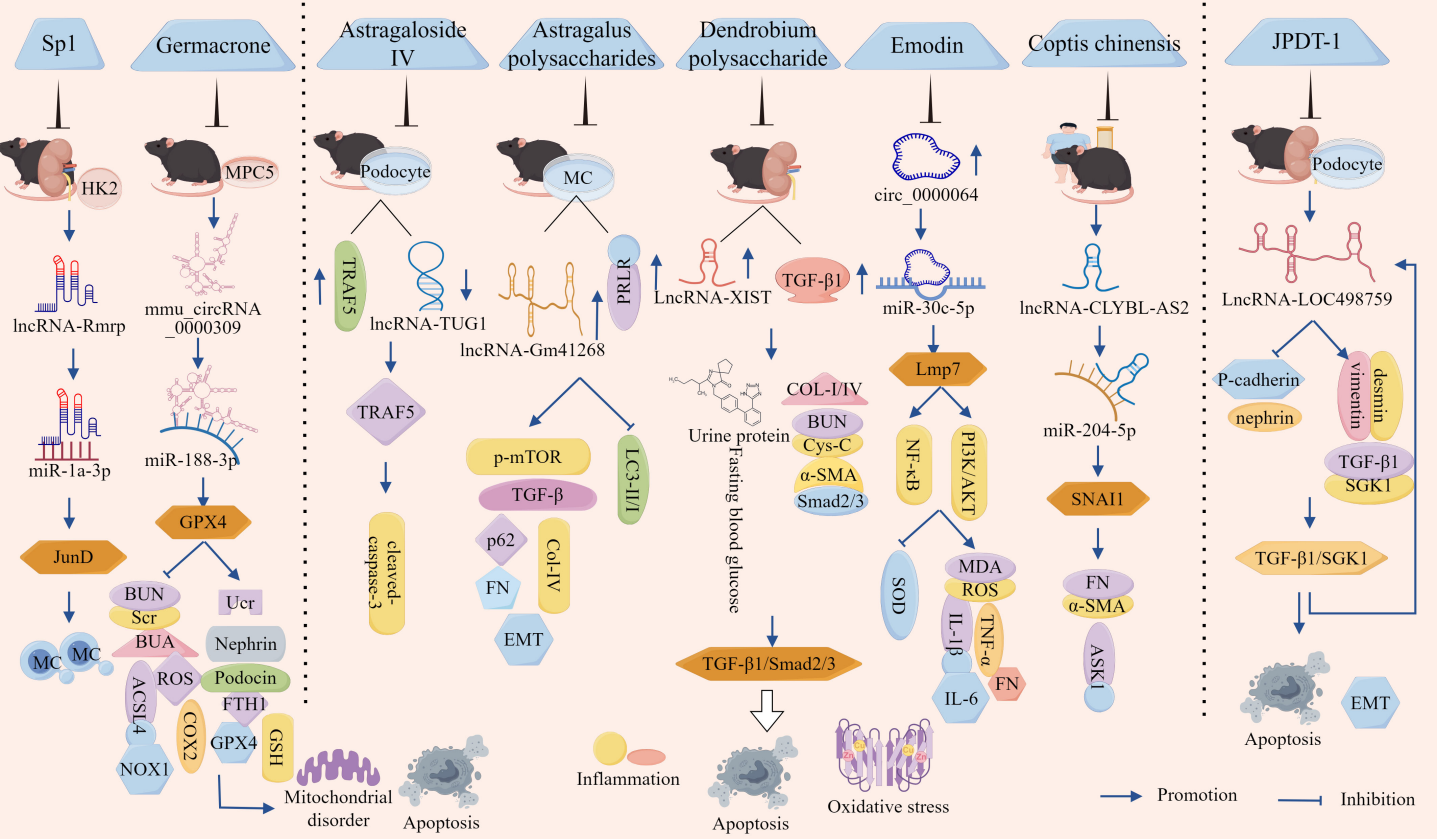
Targeted regulation of ncRNA intervenes in DN progression. The transcription factor Sp1 competes for binding to miR-1a-3p by regulating lncRNA-Rmrp, deregulating the inhibitory effect of miR-1a-3p on JunD, promoting MC proliferation and fibrosis, and thus driving DN progression. Germacrone reverses mmu_circRNA_0000309/miR-188-3p/GPX4 axis activation damage to podocytes. In addition, Astragaloside IV promoted the ubiquitinated degradation of TRAF5 protein by up-regulating lncRNA-TUG1, thereby reducing podocyte apoptosis and ameliorating DN pathology. Astragalus polysaccharide promoted autophagy and inhibited renal fibrosis by targeting the lncRNA-Gm41268/PRLR pathway; Dendrobium polysaccharide slowed down the process of renal interstitial fibrosis in DN by inhibiting the expression of LncRNA-XIST, which in turn down-regulated TGF-β1 and its downstream pro-fibrotic signals Smad2/3. Emodin inhibited the NF-κB and PI3K/AKT pathways by regulating the circ_0000064/miR-30c-5p/Lmp7 axis, thereby alleviating HG-induced OS, inflammation, and fibrosis in thylakoid cells. Coptis chinensis reversed EMT and fibrosis and ameliorated DN pathological process by inhibiting lncRNA-CLYBL/miR-204-5p/SNAI1 axis. JPDT-1 improved DN through TGF-β1/SGK1/LncRNA-LOC498759 axis.

In recent years, the proposition of the ceRNA mechanism has established targeted intervention in ceRNA networks as a novel therapeutic strategy for DN (154, 155). Research has revealed that biomolecules, functioning as operational executors of biological processes, constitute sophisticated regulatory networks through dynamic interactions, playing a pivotal role in modulating DN progression through ceRNA network interventions (**Figure 8**, **Table 6**). For instance, studies have demonstrated that lncRNA-Rmrp is remarkably up-regulated in the kidneys of DN mice and HG-treated GMCs. Rmrp overexpression markedly promoted MC proliferation and fibrosis, whereas Rmrp inhibition reversed these effects (156). Research further revealed that Sp1 transcription factor overexpression up-regulated Rmrp expression, while KO-Sp1 produced the opposite outcome, indicating transcriptional regulation of Rmrp by Sp1 (156). Moreover, investigations identified JunD as being up-regulated in DN and serving as a direct target of miR-1a-3p. This microRNA suppressed MC proliferation and fibrosis through JunD inhibition, with KO-JunD counteracting the pro-proliferative and pro-fibrotic effects induced by Rmrp overexpression (156). Collectively, these findings demonstrate that transcription factor Sp1 facilitates DN progression by modulating the lncRNA-Rmrp/miR-1a-3p/JunD axis: Sp1-mediated regulation of Rmrp enables competitive binding to miR-1a-3p, thereby relieving miR-1a-3p’s suppression of JunD and ultimately promoting MC proliferation and fibrosis. Therefore, targeting and inhibiting the transcription factor Sp1 to intervene in the lncRNA-Rmrp/miR-1a-3p/JunD axis provides a new approach for treating DN.

**Table 6:** Targeted regulation of ncRNA intervenes in DN progression.

Biomolecule/ TCM	Spongy molecule	miRNA	mRNA	Study subjects	Functional experiments	Mechanism of action	Refs
Sp1	lncRNA- Rmrp	1a-3p	JunD	DN mouse kidneys and HG-treated MCs	Gain and loss	Down-regulated: MC proliferation, fibrillation	(156)
Germacrone	mmu_circRNA_0000309	188-3p	GPX4	DN mice and MPC5 cells	Loss	Down-regulated: Ucr, Nephrin, Podocin, FTH1, GSH Up-regulated: 24hUP, BUN, Scr, BUA, ROS, COX2, ACSL4, NOX1	(157)
Astragaloside IV	lncRNA- TUG1	/	TRAF5	DN rats and HG-induced podocytes	Gain and loss	Down-regulated: Apoptosis rate, cleaved caspase-3	(158)
Astragalus polysaccharides (APS)	lncRNA- Gm41268	/	PRLR	*db/db* mouse kidney and HG-induced NRK-52E	Loss	Down-regulated: 24hUP, FBG, OGTT, p-mTOR, p62, TGF-β, Col-IV, FN Up-regulated: LC3-II/I	(30)
Dendrobium polysaccharides	LncRNA- XIST	/	TGF-β1	*db/db* mouse kidney tissue	Loss	Down-regulated: FBG, urinary protein excretion rate, BUN, Cys-C, TGF-β1, COL-I, COL-IV, α-SMA, Smad2/3	(159)
Emodin	circ_ 0000064	30c-5p	Lmp7	HG-induced MCs	Gain and loss	Down-regulated: MDA, ROS, IL-1β, IL-6, TNF-α, FN, Col-I, NF-κB, PI3K/AKT Up-regulated: SOD	(160)
Coptis chinensis	lncRNA- CLYB	204-5p	SNAI1	DN patients, mouse serum, and HK-2 cells	Gain and loss	Down-regulated: FN, α-SMA, ASK1	(161)
Jixuepaidu Tang-1 (JPDT-1)	LncRNA-LOC498759	/	TGF-β1/SGK1	DN mouse kidney tissue and HG-stimulated podocytes	Gain and loss	Down-regulated: desmin, vimentin, TGF-β1, SGK, 24hUP, HG, ECM, apoptosis Up-regulated: nephrin, P-cadherin	(162)

Beyond targeting the lncRNA/miRNA/mRNA axis to regulate DN progression, biomolecules can also exert similar therapeutic effects through modulation of the circRNA/miRNA/mRNA axis. Jin et al. demonstrated that mmu_circRNA_0000309 expression was significantly reduced in DN mice and MPC5 cells, concomitant with elevated 24-hour proteinuria, renal function indices BUN, Scr, BUA, ROS/lipid ROS production, and ferroptosis-related proteins COX2, ACSL4, NOX1, alongside decreased Ucr levels, podocyte foot process markers Nephrin, Podocin, and antioxidant indicators FTH1, GPX4, GSH. Germacrone intervention reversed these pathological changes and ameliorated DN progression, whereas KO-mmu_circRNA_0000309 or miR-188-3p mimics abolished Germacrone’s anti-apoptotic and cytoprotective effects (157). Mechanistic investigations revealed miR-188-3p as a sponge target of mmu_circRNA_0000309, with circRNA KO significantly enhancing miR-188-3p expression. Furthermore, GPX4 was identified as a downstream target of miR-188-3p through direct binding, exhibiting positive correlation with mmu_circRNA_0000309 levels (157). This molecular axis delineates a ceRNA regulatory mechanism wherein mmu_circRNA_0000309 functions as a miR-188-3p sponge, subsequently elevating GPX4 bioavailability to mitigate ferroptotic mitochondrial damage and attenuate podocyte apoptotic signaling – establishing a novel pathomechanistic framework for DN therapeutics (**Figure 8**, **Table 6**).

### 5.2 Targeted regulation of ncRNA network by TCM intervenes in DN progression

The escalating prevalence of DN has intensified the demand for improved therapeutic strategies. While conventional Western pharmaceuticals demonstrate partial efficacy, their associated adverse effects have diminished patient acceptance and compliance, necessitating the development of safer, more effective, and cost-efficient therapeutic alternatives (163, 164). Advances in molecular biological techniques have revealed that TCM operates through a ‘multi-component, multi-target, multi-pathway’ regulatory paradigm. This systemic approach not only enables targeted modulation of ncRNAs or ceRNA networks - harmonizing holistic regulation with precision medicine principles - but also offers superior safety and cost-effectiveness compared to conventional drug therapies, thereby enhancing patient receptiveness to TCM-based interventions (165–167). Moreover, bioactive TCM constituents (both single agents and compound formulations) have demonstrated unique therapeutic advantages in DN management by targeting ncRNA/ceRNA networks to impede disease progression. Their mechanisms of action exhibit remarkable congruence with core TCM theoretical tenets of ‘holistic perspective’ and ‘dynamic equilibrium’, further validating their clinical relevance (168, 169).

#### 5.2.1 Herbal monomers targeting and regulating ncRNAs intervene in DN progression

Experimental investigations revealed significantly elevated TRAF5 levels and reduced lncRNA-TUG1 expression in both DN rat models and HG-induced podocytes. Astragaloside IV treatment effectively reversed these pathological alterations (158). Mechanistic studies further demonstrated physical interaction between TUG1 and TRAF5, with TUG1 overexpression specifically promoting TRAF5 degradation via the ubiquitin-proteasome pathway. Notably, TUG1 transfection counteracted Astragaloside IV’s suppressive effects on TRAF5, confirming that Astragaloside IV inhibits TRAF5 expression through TUG1 up-regulation (158). Additionally, HG conditions markedly increased podocyte apoptosis rates and caspase-3 protein levels, both of which were significantly attenuated by Astragaloside IV treatment, TUG1 transfection abolished this cytoprotective effect (158). These findings collectively demonstrate that Astragaloside IV ameliorates DN progression by enhancing lncRNA-TUG1-mediated ubiquitin-proteasomal degradation of TRAF5 protein, thereby reducing podocyte apoptosis.

Furthermore, *in vivo* studies revealed that *db/db* mice exhibited mesangial expansion, glomerular basement membrane thickening, increased fibrosis, and tubulointerstitial fibrosis in the kidney tubular region, these animals also demonstrated significantly elevated 24-hour urinary protein levels, fasting blood glucose concentrations, and area under the curve (AUC) values in oral glucose tolerance tests (OGTT), along with marked up-regulation of lncRNA-Gm41268 and PRLR expression (30). Both *in vivo* and *in vitro* experiments demonstrated that Astragalus polysaccharide treatment effectively reversed these pathological alterations, ameliorated renal impairment, and concurrently reduced p-mTOR, p62, TGF-β, Col-IV, and FN, the intervention notably enhanced the LC3-II/I ratio, indicating that Astragalus polysaccharides (APS) alleviated HG-induced autophagy inhibition and renal fibrosis (30). Crucially, subsequent investigations revealed that either pharmacological inhibition of Gm41268 or genetic KO-PRLR abolished the renal protective effects mediated by APS (30). Collectively, these findings demonstrate that APS mitigate DN progression by enhancing autophagy and suppressing renal fibrosis through targeted modulation of the lncRNA-Gm41268/PRLR signaling pathway.

Furthermore, investigations demonstrated significantly elevated expression of LncRNA-XIST and TGF-β1 in renal tissues of *db/db* mice, with notable co-localization observed between XIST and TGF-β1 (159). Intervention with Dendrobium polysaccharides or KO-XIST substantially attenuated renal pathological manifestations, manifesting attenuation of tubulointerstitial fibrotic lesions, amelioration of GMCs hypertrophy, and preservation of podocyte foot process architecture (159). These interventions significantly decreased fasting blood glucose levels, urinary protein excretion rate, BUN, Cys-C concentrations, and kidney-to-body weight ratio. Concurrently, marked down-regulation was observed in XIST and TGF-β1 expression, alongside reduced levels of fibrosis biomarkers COL-I, COL-IV, α-SMA, and phosphorylated Smad2/3, indicating suppression of the TGF-β1/Smad2/3 signaling pathway (159). These findings collectively suggest that Dendrobium polysaccharides mitigate DN progression by inhibiting LncRNA-XIST expression, thereby downregulating TGF-β1 and its downstream profibrotic effector Smad2/3, ultimately delaying renal interstitial fibrosis in DN.

As natural small-molecule compounds, herbal monomers not only modulate ncRNA expression to influence DN progression but also regulate ceRNA networks to intervene in DN pathogenesis. For instance, studies demonstrated that under HG conditions, elevated levels of MDA, ROS, IL-1β, IL-6, TNF-α, FN, and Col-I were observed alongside reduced SOD activity, these alterations intensified OS, inflammatory responses, fibrotic processes, and cellular damage, all of which were dose-dependently reversed by emodin treatment (160). Mechanistically, HG-induced up-regulation of circ_0000064 expression was suppressed by emodin. Crucially, overexpression of circ_0000064 abolished the protective effects of emodin, while circ_0000064 was shown to directly bind miR-30c-5p and inhibit its activity. miR-30c-5p itself targets the 3’ UTR of Lmp7 to suppress its expression (159). KO-circ_0000064 or overexpression of miR-30c-5p significantly reduced Lmp7 levels, ameliorating HG-induced cellular injury. Furthermore, emodin counteracted HG-induced activation of the NF-κB and PI3K/AKT pathways by inhibiting Lmp7, whereas Lmp7 overexpression neutralized emodin’s suppressive effects on these pathways (160). Collectively, these findings demonstrate that emodin alleviates HG-induced mesangial cell OS, inflammation, and fibrosis through modulation of the circ_0000064/miR-30c-5p/Lmp7 axis, thereby suppressing NF-κB and PI3K/AKT signaling.

Cai et al. demonstrated significantly down-regulated miR-204-5p and up-regulated SNAI1 and lncRNA-CLYBL-AS2 in the serum of DN patients and murine models, CLYBL-AS2 exhibited a negative correlation with miR-204-5p and a positive association with SNAI1 expression and DN staging (161). Under HG conditions, CLYBL-AS2 overexpression in HK-2 cells reduced miR-204-5p levels while elevating SNAI1 expression, thereby promoting cellular migration, invasion, and the expression of fibrotic biomarkersFN, α-SMA, and ASK1. Conversely, KO-CLYBL-AS2 suppressed these pathological phenotypes (161). Dual-luciferase reporter assays confirmed that CLYBL-AS2 shares binding sites with miR-204-5p, competitively inhibiting miR-204-5p-mediated targeting of SNAI1, Intervention with Coptis chinensis significantly reduced CLYBL-AS2 expression in DN mice in a dose-dependent manner (161). These findings indicate that Coptis chinensis ameliorates DN progression by suppressing the lncRNA-CLYBL-AS2/miR-204-5p/SNAI1 axis, thereby reversing EMT and fibrotic processes.

Overall, herbal monomers regulate DN progression by modulating ncRNA expression or interfering with ceRNA networks (**Figure 8**, **Table 6**). This discovery not only expands the functional understanding of ncRNAs and ceRNA networks in DN pathogenesis but also provides novel evidence for the nephroprotective effects of herbal monomers. Furthermore, it establishes a critical theoretical foundation for developing natural product-based therapeutic strategies against DN and offers experimental insights into mechanistic exploration and targeted therapies for this condition.

#### 5.2.2 Targeted regulation of ncRNA by Chinese medicine compound intervenes in DN progression

Traditional herbal formulae, grounded in the Jun-Chen-Zuo-Shi compatibility principle, exert multi-dimensional interventions in DN progression through synergistic multi-target effects. These formulations not only circumvent drug resistance inherent to single-target pharmacological agents but also enable coordinated targeting of critical ncRNA nodes via their multi-component synergy, thereby achieving comprehensive modulation of DN pathogenesis (170, 171).

Jin et al. demonstrated that HG stimulation remarkably suppressed podocyte viability and promoted apoptosis, while treatment with Jixuepaidu Tang-1 (JPDT-1) markedly reversed these HG-induced alterations (162). Further investigations revealed that JPDT-1 up-regulated epithelial markers nephrin, P-cadherin and down-regulated mesenchymal markers desmin, vimentin, effectively counteracting HG-triggered EMT in podocytes (162). Additionally, JPDT-1 reduced HG-elevated levels of TGF-β1/SGK1, notably, recombinant TGF-β1 or SGK1 administration abolished the protective effects of JPDT-1, whereas SGK1 silencing produced opposing outcomes (162). These findings collectively indicate that JPDT-1 mitigates HG-induced podocyte injury and EMT progression through suppression of the TGF-β1/SGK1 signaling axis. Further investigations revealed elevated expression of lncRNA-LOC498759 in renal tissues of DN mice and HG-stimulated podocytes, JPDT-1 significantly suppressed LOC498759 expression, whereas LOC498759 overexpression attenuated JPDD-1-mediated inhibition of EMT. Conversely, KO-LOC498759 potentiated its protective effects (162). Additionally, TGF-β1/SGK1 pathway activation up-regulated LOC498759 expression, though LOC498759 overexpression itself exerted no significant regulatory effect on TGF-β1/SGK1 levels, suggesting LOC498759 operates downstream of this signaling axis (162). JPDT-1 treatment reduced kidney-to-body weight ratio, 24-hour urinary protein excretion, and blood glucose levels in DN mice. Histologically, it ameliorated glomerular matrix deposition and renal apoptosis while downregulating renal TGF-β1, SGK1, and mesenchymal marker expression. Notably, LOC498759 overexpression diminished JPDD-1’s renoprotective effects and EMT suppression, yet had no significant impact on TGF-β1/SGK1 signaling activity, further corroborating LOC498759’s position downstream of this pathway (162).

Collectively, these findings demonstrate that JPDT-1 ameliorates DN progression through modulation of the TGF-β1/SGK1/lncRNA-LOC498759 axis (**Figure 8**, **Table 6**). This not only provides experimental validation for the modernization of traditional herbal formulae but also offers critical mechanistic insights and potential clinical relevance for their application in DN treatment.

As can be seen from the previous discussion, although biological molecule-targeted regulation of the ceRNA network has shown some effectiveness in regulating DN progression; however, because of the complementary nature of the ncRNA seed region with non-target genes, the uptake of the vector by non-target cells, and the saturation of the RNAi mechanism at high ncRNA doses, as well as interference with the endogenous miRNA pathway, the specificity of ncRNA target genes is relatively poor. Consequently, off-target effects frequently occur when ncRNA-targeted therapies are used (172). Second, during DN, reactions such as inflammation and OS occur, causing changes in the renal microenvironment and significantly reducing intracellular release efficiency. The glomerular filtration barrier and the repulsion of charges on the basement membrane result in the clearance of numerous carriers, with only a small fraction reaching the kidneys. Unmodified renal targeting ligands on the carrier surface, as well as serum nuclease degradation and opsonin effects, cause the intervention drugs to accumulate non-specifically in the liver/spleen as well as reduce the half-life of the drug, leading to low delivery efficiency (173). Finally, because of the differing sensitive targets of ncRNAs across different DN subtypes (inflammatory and fibrotic types), generic therapies may be ineffective or damage normal kidney tissue, advanced DN glomerulosclerosis reduces carrier permeability, necessitating the development of penetration-enhancing peptides to enhance carrier penetration capacity (174). Therefore, the treatment of DN presents a unique challenge.

The low bioavailability of TCM components can be attributed to several factors. First, the low transmembrane permeability of large polar molecules limits their absorption. In addition, the metabolism by liver CYP450 enzymes results in a strong first-pass effect, and the degradation of glycoside components by intestinal microbiota further reduces bioavailability. Second, most TCM components cannot accumulate in diseased tissues, and their strong binding to plasma proteins reduces free drug concentrations, thus leading to impaired distribution of TCM components within the body. The complex interactions between multiple components and active metabolites in TCM also result in unpredictable metabolism. Furthermore, owing to the high molecular weight of TCM components, which cannot be filtered by the glomeruli, a high risk of accumulation and prolonged half-life exists in the enterohepatic circulation, leading to impaired drug excretion (175). Therefore, although strategies based on bioactive molecules and targeted regulation of ncRNAs in TCM show great potential in the prevention and treatment of DN, their clinical translation still faces multiple cross-scale bottlenecks (delivery efficiency, off-target effects, DN heterogeneity of diabetic nephropathy, and safety), requiring further study.

## 6 Summarization and prospect

This review summarizes the roles of ncRNAs, ceRNA networks, and DN, as well as the regulation of DN progression through ncRNA intervention and targeted modulation of ncRNA intervention in DN progression. This study specifically highlights the regulatory roles of ceRNA networks in modulating renal cell responses to DN progression through cell death, fibrosis, and cell death–fibrosis pathways, the roles of exosomes in modulating DN progression through miRNA, circRNA–miRNA–mRNA, and stem cell exosomes through ncRNA networks, the roles of biomolecular targeted regulation of ceRNA networks in modulating DN progression, and the roles of single Chinese herbal compounds and Chinese herbal formulae in modulating DN progression through targeted regulation of ncRNAs. ncRNAs, ceRNA networks, exosomal ncRNAs, and stem cell exosomal ncRNAs all play important regulatory roles in DN progression. Among these, exosome ncRNAs and stem cell exosome ncRNAs are crucial for DN progression and have the potential for cell-free therapy. In addition, a summary of ncRNA-targeted therapies for DN using biomolecules, single Chinese herbal medicine components, and Chinese herbal medicine formulas revealed that biomolecular-targeted regulation of the ceRNA network has shown some efficacy in regulating DN progression. However, owing to off-target effects, low delivery efficiency, and the special nature of DN treatment during ncRNA therapy, these results have not been particularly outstanding. Due to the low bioavailability of herbal compounds, suboptimal *in vivo* distribution, and impaired drug excretion, research on TCM formulations targeting ncRNA-mediated regulation of DN progression remains limited. This limitation creates multi-scale translational barriers in clinical applications. Furthermore, while our article comprehensively summarizes ncRNA regulatory mechanisms in DN progression through cell death, fibrosis, and their interactions, it does not address ncRNA-mediated interventions via other pathways, resulting in incomplete mechanistic coverage.

This review also showed that ncRNAs can simultaneously reflect inflammation, fibrosis, metabolic disorders, and other conditions and are thereby associated with multiple mechanisms. They are currently used in clinical settings. For example, urinary miRNAs and serum circRNAs have been used for early DN screening (176, 177), and lncRNAs, miRNAs, and circRNAs have been used for disease classification (inflammation-dominant type, fibrosis-progressive type, and metabolic disorder type) (101). In addition, miRNA inhibitors or miRNA mimics have been used in the treatment of cardiovascular and immune-related diseases (178), and lncRNA inhibitors and circRNA interventions are currently used in the management of neoplastic diseases (179, 180). Thus, ncRNAs have transitioned from the mechanistic exploration to the clinical validation phase and have demonstrated irreplaceable value.

However, owing to potential RNase attacks, pH fluctuations leading to hydrolysis, repeated freeze–thaw cycles causing damage, and temperature-induced degradation during the process from sample collection to analysis, free ncRNAs may experience a shortened half-life, accelerated degradation rate, increased loss rate, and reduced integrity, resulting in poor stability in bodily fluids. In addition, because of differences in the inherent stability of lncRNAs, miRNAs, and circRNAs, as well as the characteristics of samples from different sources (ncRNA in urine is more susceptible to degradation than in serum) and the higher stability of ncRNAs encapsulated in exosomes compared to free forms, it is challenging to improve the stability of bodily fluids. Significant differences in the results were also observed owing to variations in sample preprocessing, RNA extraction, reverse transcription, and quantification platforms. Deficiencies in detection standardization have been attributed to factors such as missing reference genes, inconsistent threshold settings, and conflicting platform sensitivities. Finally, circRNAs and lncRNAs exhibit phylogenetic conservation, indicating that they are highly conserved and have species-specific conservation (primate-specific) across mammalian species, such as humans and mice, and also follow a conservation-tissue association pattern, with the circRNA conservation ranking as follows: brain tissue > kidney > liver; lncRNA conservation ranking: embryo development-related > tissue-specific. These factors have significantly hindered the basic research and clinical applications of ncRNAs.

The study further revealed that the ceRNA mechanism necessitates miRNAs to be in a sub-saturation state (incompletely bound to targets), however, in most cells, miRNA expression substantially exceeds that of their targets (mRNAs/lncRNAs), consequently, under physiological conditions, only a minority of high-abundance ncRNAs may satisfy this requirement; Secondly, two ceRNAs must share more than 40% of the miRNA pool and have similar expression levels in order to regulate each other, a condition that is extremely rare in physiological environments; Additionally, most ceRNA interaction studies rely on overexpression systems, which induce non-physiological miRNA sponge effects and thus fail to reflect authentic physiological states; Furthermore, cells possess multiple redundant miRNA regulatory backup mechanisms, therefore, ceRNA networks likely function as fine-tuning modulators rather than dominant factors (181). Collectively, although the ceRNA mechanism may amplify in pathological contexts, its contribution in homeostatic physiological systems is likely overestimated—it appears to act primarily as a “noise buffer” within gene regulatory networks, not as a primary driver. Consequently, before clinical application, the robustness, disease-specific mechanisms, and therapeutic efficacy of ceRNA-based approaches require further validation.

It is essential to strengthen research on the regulatory roles of ncRNAs, ceRNA, exosomal ncRNAs, stem cell exosomal ncRNAs, ceRNA networks, biomolecules, and drug-targeted regulation of ncRNA/ceRNA networks in the progression of DN. Particularly, in the study of ncRNA and ceRNA networks, it is necessary to innovate sample processing methods, such as using time-stable techniques, exosome-locking technologies, and enhanced artificial modifications to enhance the stability of bodily fluids. In addition, it is essential to establish unified technical standards, such as strictly adhering to the MIQE guidelines, promoting the SPIRE standard, and conducting bioinformatics corrections, including algorithm normalization and artificial intelligence calibration, to achieve consistency between laboratory variables and clinical outcomes. Furthermore, the cross-species conservation of ncRNAs must be considered, such as whether mouse-conserved circRNAs and lncRNAs can predict human efficacy in model selection. Although highly conserved ncRNA targets are more likely to succeed in clinical translation, the limitations of validating human-specific ncRNAs in animal models must be considered. In the field of ceRNA research, it is essential to develop single-cell-level ceRNA kinetic models, employ endogenous gene-tagged KO/knock-in models, and integrate multi-omics data to construct panoramic views of ceRNA-protein interaction networks.

In the study of exosomal ncRNAs and stem cell exosomal ncRNAs, it is essential to establish standardized procedures for the preparation and quality control of both and to explore effective methods for obtaining functionally uniform, high-purity exosomal samples to ensure the accuracy and reproducibility of experimental results. In addition, no specific methods are currently available to reverse or halt the progression of DN. Although ncRNAs, exosome ncRNAs, stem cell exosome ncRNAs, ceRNA networks, biomolecules, and TCMs have shown potential for treating DN, their efficacy still needs improvement. Therefore, future research should assess the safety of treatment methods in relation to DN and explore the use of stem cell gene engineering, biotechnology, immunosuppressive therapy, and multitherapy synergism to enhance efficacy and improve treatment outcomes. Only by overcoming these challenges can ncRNA and ceRNA networks become core tools for the prevention and treatment of DN, ultimately achieving a diagnostic and therapeutic closed-loop system characterized by noninvasive classification, targeted intervention, and real-time assessment.

## References

[1] Umpierrez GE Davis GM ElSayed NA Fadini GP Galindo RJ Hirsch IB. Hyperglycemic crises in adults with diabetes: A consensus report. Diabetes Care. (2024) 47:1257–75. doi: 10.2337/dci24-0032, PMID: 39052901 PMC11272983

[2] Zheng W Guo J Lu X Qiao Y Liu D Pan S. cAMP-response element binding protein mediates podocyte injury in diabetic nephropathy by targeting lncRNA DLX6-AS1. Metabolism. (2022) 129:155155. doi: 10.1016/j.metabol.2022.155155, PMID: 35093327

[3] Selby NM Taal MW. An updated overview of diabetic nephropathy: Diagnosis, prognosis, treatment goals and latest guidelines. Diabetes Obes Metab. (2020) 1:3–15. doi: 10.1111/dom.14007, PMID: 32267079

[4] Jin Q Liu T Qiao Y Liu D Yang L Mao H. Oxidative stress and inflammation in diabetic nephropathy: role of polyphenols. Front Immunol. (2023) 14:1185317. doi: 10.3389/fimmu.2023.1185317, PMID: 37545494 PMC10401049

[5] Jiang WJ Xu CT Du CL Dong JH Xu SB Hu BF. Tubular epithelial cell-to-macrophage communication forms a negative feedback loop via extracellular vesicle transfer to promote renal inflammation and apoptosis in diabetic nephropathy. Theranostics. (2022) 12:324–39. doi: 10.7150/thno.63735, PMID: 34987648 PMC8690920

[6] Liu Z Nan P Gong Y Tian L Zheng Y Wu Z. Endoplasmic reticulum stress-triggered ferroptosis via the XBP1-Hrd1-Nrf2 pathway induces EMT progression in diabetic nephropathy. BioMed Pharmacother. (2023) 164:114897. doi: 10.1016/j.biopha.2023.114897, PMID: 37224754

[7] Gupta S Dominguez M Golestaneh L. Diabetic kidney disease: an update. Med Clin North Am. (2023) 107:689–705. doi: 10.1016/j.mcna.2023.03.004, PMID: 37258007

[8] Yan H Zhou Q Wang Y Tu Y Zhao Y Yu J. Associations between cardiometabolic indices and the risk of diabetic kidney disease in patients with type 2 diabetes. Cardiovasc Diabetol. (2024) 23:142. doi: 10.1186/s12933-024-02228-9, PMID: 38664793 PMC11046854

[9] Denhez B Rousseau M Dancosst DA Lizotte F Guay A Auger-Messier M. Diabetes-induced DUSP4 reduction promotes podocyte dysfunction and progression of diabetic nephropathy. Diabetes. (2019) 68:1026–39. doi: 10.2337/db18-0837, PMID: 30862678

[10] Jiang S Su H. Cellular crosstalk of mesangial cells and tubular epithelial cells in diabetic kidney disease. Cell Commun Signal. (2023) 21:288. doi: 10.1186/s12964-023-01323-w, PMID: 37845726 PMC10577991

[11] Wang H Yu X Liu D Qiao Y Huo J Pan S. VDR activation attenuates renal tubular epithelial cell ferroptosis by regulating nrf2/HO-1 signaling pathway in diabetic nephropathy. Adv Sci (Weinh). (2024) 11:e2305563. doi: 10.1002/advs.202305563, PMID: 38145959 PMC10933633

[12] Chen LL Kim VN. Small and long non-coding RNAs: Past, present, and future. Cell. (2024) 187:6451–85. doi: 10.1016/j.cell.2024.10.024, PMID: 39547208

[13] Butz H Patócs A Igaz P. Circulating non-coding RNA biomarkers of endocrine tumours. Nat Rev Endocrinol. (2024) 20:600–14. doi: 10.1038/s41574-024-01005-8, PMID: 38886617

[14] Młynarska E Buławska D Czarnik W Hajdys J Majchrowicz G Prusinowski F. Novel insights into diabetic kidney disease. Int J Mol Sci. (2024) 25:10222. doi: 10.3390/ijms251810222, PMID: 39337706 PMC11432709

[15] Chen Y He Y Zhou H. The potential role of lncRNAs in diabetes and diabetic microvascular complications. Endocr J. (2020) 67:659–68. doi: 10.1507/endocrj.EJ19-0574, PMID: 32404556

[16] Wang Q Zhu Y Dong Q Zhang L Zhang W. A novel circ_Arf3/miR-452-5p/mbnl1 axis regulates proliferation and expression of fibrosis-related proteins of mouse mesangial cells under high glucose. Diabetes Metab Syndr Obes. (2023) 16:2105–16. doi: 10.2147/DMSO.S400, PMID: 37457110 PMC10349572

[17] Tang B Li W Ji TT Li XY Qu X Feng L. Circ-AKT3 inhibits the accumulation of extracellular matrix of mesangial cells in diabetic nephropathy via modulating miR-296-3p/E-cadherin signals. J Cell Mol Med. (2020) 24:8779–88. doi: 10.1111/jcmm.15513, PMID: 32597022 PMC7412430

[18] Wu R Niu Z Ren G Ruan L Sun L. CircSMAD4 alleviates high glucose-induced inflammation, extracellular matrix deposition and apoptosis in mouse glomerulus mesangial cells by relieving miR-377-3p-mediated BMP7 inhibition. Diabetol Metab Syndr. (2021) 13:137. doi: 10.1186/s13098-021-00753-1, PMID: 34801077 PMC8606083

[19] Wang T Cui S Liu X Han L Duan X Feng S. LncTUG1 ameliorates renal tubular fibrosis in experimental diabetic nephropathy through the miR-145-5p/dual-specificity phosphatase 6 axis. Ren Fail. (2023) 45:2173950. doi: 10.1080/0886022X.2023.2173950, PMID: 36794657 PMC9937007

[20] He YX Wang T Li WX Chen YX. Long noncoding RNA protein-disulfide isomerase-associated 3 regulated high glucose-induced podocyte apoptosis in diabetic nephropathy through targeting miR-139-3p. World J Diabetes. (2024) 15:260–74. doi: 10.1080/0886022X.2023.2173950, PMID: 38464366 PMC10921158

[21] Meldolesi J. Exosomes and ectosomes in intercellular communication. Curr Biol. (2018) 28:R435–44. doi: 10.1016/j.cub.2018.01.059, PMID: 29689228

[22] Kalluri R LeBleu VS. The biology, function, and biomedical applications of exosomes. Science. (2020) 367:eaau6977. doi: 10.1126/science.aau6977, PMID: 32029601 PMC7717626

[23] Iqbal Z Rehman K Mahmood A Shabbir M Liang Y Duan L. Exosome for mRNA delivery: strategies and therapeutic applications. J Nanobiotechnol. (2024) 22:395. doi: 10.1186/s12951-024-02634-x, PMID: 38965553 PMC11225225

[24] Wei L Liu L Bai M Ning X Sun S. CircRNAs: versatile players and new targets in organ fibrosis. Cell Commun Signal. (2023) 21:90. doi: 10.1186/s12964-023-01051-1, PMID: 37131173 PMC10152639

[25] Neuen BL Heerspink HJL Vart P Claggett BL Fletcher RA Arnott C. Estimated lifetime cardiovascular, kidney, and mortality benefits of combination treatment with SGLT2 inhibitors, GLP-1 receptor agonists, and nonsteroidal MRA compared with conventional care in patients with type 2 diabetes and albuminuria. Circulation. (2024) 149:450–62. doi: 10.1161/CIRCULATIONAHA.123.067584, PMID: 37952217

[26] Sugahara M Pak WLW Tanaka T Tang SCW Nangaku M. Update on diagnosis, pathophysiology, and management of diabetic kidney disease. Nephrol (Carlton). (2021) 26:491–500. doi: 10.1111/nep.13860, PMID: 33550672

[27] Tian J Jin D Bao Q Ding Q Zhang H Gao Z. Evidence and potential mechanisms of traditional Chinese medicine for the treatment of type 2 diabetes: A systematic review and meta-analysis. Diabetes Obes Metab. (2019) 21:1801–16. doi: 10.1111/dom.13760, PMID: 31050124

[28] Liu XJ Hu XK Yang H Gui LM Cai ZX Qi MS. A review of traditional chinese medicine on treatment of diabetic nephropathy and the involved mechanisms. Am J Chin Med. (2022) 50:1739–79. doi: 10.1142/S0192415X22500744, PMID: 36222120

[29] Xu W Li B Xu M Yang T Hao X. Traditional Chinese medicine for precancerous lesions of gastric cancer: A review. BioMed Pharmacother. (2022) 146:112542. doi: 10.1016/j.biopha.2021.112542, PMID: 34929576

[30] Chen Z Liang H Yan X Liang Q Bai Z Xie T. Astragalus polysaccharide promotes autophagy and alleviates diabetic nephropathy by targeting the lncRNA Gm41268/PRLR pathway. Ren Fail. (2023) 45:2284211. doi: 10.1080/0886022X.2023.2284211, PMID: 37994436 PMC11001349

[31] Wang J Wang X Ma T Xie Y. Research progress on Alpinia oxyphylla in the treatment of diabetic nephropathy. Front Pharmacol. (2024) 15:1390672. doi: 10.3389/fphar.2024.1390672, PMID: 38948461 PMC11211572

[32] Gonzales LR Blom S Henriques R Bachem CWB Immink RGH. LncRNAs: the art of being influential without protein. Trends Plant Sci. (2024) 29:770–85. doi: 10.1016/j.tplants.2024.01.006, PMID: 38368122

[33] Liang WW Müller S Hart SK Wessels HH Méndez-Mancilla A Sookdeo A. Transcriptome-scale RNA-targeting CRISPR screens reveal essential lncRNAs in human cells. Cell. (2024) 187:7637–7654.e29. doi: 10.1016/j.cell.2024.10.021, PMID: 39532094 PMC11682925

[34] Bao Y Teng S Zhai H Zhang Y Xu Y Li C. SE-lncRNAs in cancer: classification, subcellular localisation, function and corresponding TFs. J Cell Mol Med. (2024) 28:e70296. doi: 10.1111/jcmm.70296, PMID: 39690143 PMC11652108

[35] Gluba-Sagr A Franczyk B Rysz-Górzyńska A Olszewski R Rysz J. The role of selected lncRNAs in lipid metabolism and cardiovascular disease risk. Int J Mol Sci. (2024) 25:9244. doi: 10.3390/ijms25179244, PMID: 39273193 PMC11395304

[36] Nadhan R Isidoro C Song YS Dhanasekaran DN. Signaling by lncRNAs: structure, cellular homeostasis, and disease pathology. Cells. (2022) 11:2517. doi: 10.3390/cells11162517, PMID: 36010595 PMC9406440

[37] Yu S Li Y Lu X Han Z Li C Yuan X. The regulatory role of miRNA and lncRNA on autophagy in diabetic nephropathy. Cell Signal. (2024) 118:111144. doi: 10.1016/j.cellsig.2024.111144, PMID: 38493883

[38] Li L Wu YQ Yang JE. Stress-related lncRNAs and their roles in diabetes and diabetic complications. Int J Mol Sci. (2025) 26:2194. doi: 10.3390/ijms26052194, PMID: 40076814 PMC11900361

[39] Hwang H Chang HR Baek D. Determinants of functional microRNA targeting. Mol Cells. (2023) 46:21–32. doi: 10.14348/molcells.2023.2157, PMID: 36697234 PMC9880601

[40] Li Y Chen S Rao H Cui S Chen G. MicroRNA gets a mighty award. Adv Sci (Weinh). (2025) 12:e2414625. doi: 10.1002/advs.202414625, PMID: 39836690 PMC11831481

[41] Tealdi S Ferro E Campa CC Bosia C. microRNA-mediated encoding and decoding of time-dependent signals in tumorigenesis. Biomolecules. (2022) 12:213. doi: 10.3390/biom1202013, PMID: 35204714 PMC8961662

[42] Wang H Shi J Wang J Hu Y. MicroRNA−378: An important player in cardiovascular diseases (Review). Mol Med Rep. (2023) 28:172. doi: 10.3892/mmr.2023.13059, PMID: 37503766 PMC10436248

[43] Liu X Jiang L Zeng H Gao L Guo S Chen C. Circ-0000953 deficiency exacerbates podocyte injury and autophagy disorder by targeting Mir665-3p-Atg4b in diabetic nephropathy. Autophagy. (2024) 20:1072–97. doi: 10.1080/15548627.2023.2286128, PMID: 38050963 PMC11135827

[44] Zhang H Yan Y Hu Q Zhang X. LncRNA MALAT1/microRNA let-7f/KLF5 axis regulates podocyte injury in diabetic nephropathy. Life Sci. (2021) 266:118794. doi: 10.1080/15548627.2023.2286128, PMID: 33232688

[45] Ji JL Shi HM Li ZL Jin R Qu GT Zheng H. Satellite cell-derived exosome-mediated delivery of microRNA-23a/27a/26a cluster ameliorates the renal tubulointerstitial fibrosis in mouse diabetic nephropathy. Acta Pharmacol Sin. (2023) 44:2455–68. doi: 10.1038/s41401-023-01140-4, PMID: 37596398 PMC10692096

[46] Zhang K Wu D Huang C. Crosstalk between non-coding RNA and apoptotic signaling in diabetic nephropathy. Biochem Pharmacol. (2024) 230:116621. doi: 10.1016/j.bcp.2024.116621, PMID: 39542182

[47] Yi Q Feng J Lan W Shi H Sun W Sun W. CircRNA and lncRNA-encoded peptide in diseases, an update review. Mol Cancer. (2024) 23:214. doi: 10.1186/s12943-024-02131-7, PMID: 39343883 PMC11441268

[48] Xu F Xiao Q Du WW Wang S Yang BB. CircRNA: functions, applications and prospects. Biomolecules. (2024) 14:1503. doi: 10.3390/biom14121503, PMID: 39766210 PMC11673012

[49] Miao S Yang L Xu T Liu Z Zhang Y Ding L. A novel circPIK3C2A/miR-31-5p/TFRC axis drives ferroptosis and accelerates myocardial injury. MedComm (2020). (2024) 5:e571. doi: 10.1002/mco2.571, PMID: 38840772 PMC11151151

[50] Wang H Zhao H Chen Z Cai X Wang X Zhou P. Hypoxic Bone Mesenchymal Stem Cell-Derived Exosomes Direct Schwann Cells Proliferation, Migration, and Paracrine to Accelerate Facial Nerve Regeneration via circRNA_Nkd2/miR-214-3p/MED19 Axis. Int J Nanomed. (2024) 19:1409–29. doi: 10.2147/IJN.S443036, PMID: 38371458 PMC10871042

[51] Zhang J Luo Z Zheng Y Duan M Qiu Z Huang C. CircRNA as an Achilles heel of cancer: characterization, biomarker and therapeutic modalities. J Transl Med. (2024) 22:752. doi: 10.1186/s12967-024-05562-4, PMID: 39127679 PMC11316389

[52] Peng F Gong W Li S Yin B Zhao C Liu W. circRNA_010383 Acts as a Sponge for miR-135a, and Its down-regulated Expression Contributes to Renal Fibrosis in Diabetic Nephropathy. Diabetes. (2021) 70:603–15. doi: 10.2337/db20-0203, PMID: 33472945

[53] Zhang L Jin G Zhang W Wang Q Liang Y Dong Q. CircRNA Arf3 suppresses glomerular mesangial cell proliferation and fibrosis in diabetic nephropathy via miR-107-3p/Tmbim6 axis. J Bioenerg Biomembr. (2024) 56:543–52. doi: 10.1007/s10863-024-10027-w, PMID: 39120858 PMC11455692

[54] Zhang C Yu Z Yang S Liu Y Song J Mao J. ZNF460-mediated circRPPH1 promotes TNBC progression through ITGA5-induced FAK/PI3K/AKT activation in a ceRNA manner. Mol Cancer. (2024) 23:33. doi: 10.1186/s12943-024-01944-w, PMID: 38355583 PMC10865535

[55] Kohansal M Alghanimi YK Banoon SR Ghasemian A Afkhami H Daraei A. CircRNA-associated ceRNA regulatory networks as emerging mechanisms governing the development and biophysiopathology of epilepsy. CNS Neurosci Ther. (2024) 30:e14735. doi: 10.1111/cns.14735, PMID: 38676299 PMC11053249

[56] Hsiao YW Wang L Lu TP. ceRNAR: An R package for identification and analysis of ceRNA-miRNA triplets. PloS Comput Biol. (2022) 8:e1010497. doi: 10.1371/journal.pcbi.1010497, PMID: 36084156 PMC9491567

[57] Karagkouni D Karavangeli A Paraskevopoulou MD Hatzigeorgiou AG. Characterizing miRNA-lncRNA interplay. Methods Mol Biol. (2021) 2372:243–62. doi: 10.1007/978-1-0716-1697-0_21, PMID: 34417757

[58] Wang Y Tan J Xu C Wu H Zhang Y Xiong Y. Identification and construction of lncRNA-associated ceRNA network in diabetic kidney disease. Med (Baltimore). (2021) 100:e26062. doi: 10.1097/MD.0000000000026062, PMID: 34087849 PMC8183707

[59] Zhuang L Jin G Wang Q Ge X Pei X. Long non-coding RNA ZFAS1 regulates fibrosis and scortosis in the cell model of diabetic nephropathy through miR-525-5p/SGK1 axis. Appl Biochem Biotechnol. (2024) 196:3731–46. doi: 10.1007/s12010-023-04721-5, PMID: 37768477

[60] Fu JX Sun GQ Wang HL Jiang HX. LncRNA OIP5-AS1 induces epithelial-to-mesenchymal transition and renal fibrosis in diabetic nephropathy via binding to miR-30c-5p. J Biol Regul Homeost Agents. (2020) 34:961–8. doi: 10.23812/20-199-A-68, PMID: 32519534

[61] Zhan JF Huang HW Huang C Hu LL Xu WW. Long Non-Coding RNA NEAT1 Regulates Pyroptosis in Diabetic Nephropathy via Mediating the miR-34c/NLRP3 Axis. Kidney Blood Press Res. (2020) 45:589–602. doi: 10.1159/000508372, PMID: 32721950

[62] Chen W Xu J Wu Y Liang B Yan M Sun C. The potential role and mechanism of circRNA/miRNA axis in cholesterol synthesis. Int J Biol Sci. (2023) 19:2879–96. doi: 10.7150/ijbs.84994, PMID: 37324939 PMC10266072

[63] Ma B Wang S Wu W Shan P Chen Y Meng J. Mechanisms of circRNA/lncRNA-miRNA interactions and applications in disease and drug research. BioMed Pharmacother. (2023) 162:114672. doi: 10.1016/j.biopha.2023.114672, PMID: 37060662

[64] Zhang M Bai X Zeng X Liu J Liu F Zhang Z. circRNA-miRNA-mRNA in breast cancer. Clin Chim Acta. (2021) 523:120–30. doi: 10.1016/j.cca.2021.09.013, PMID: 34537217

[65] Zheng Y Wen S Jiang S He S Qiao W Liu Y. CircRNA/lncRNA-miRNA-mRNA network and gene landscape in calcific aortic valve disease. BMC Genomics. (2023) 24:419. doi: 10.1186/s12864-023-09441-y, PMID: 37491214 PMC10367311

[66] Gao M Zhang Z Sun J Li B Li Y. The roles of circRNA-miRNA-mRNA networks in the development and treatment of osteoporosis. Front Endocrinol (Lausanne). (2022) 13:945310. doi: 10.3389/fendo.2022.945310, PMID: 35992137 PMC9388761

[67] Li Y Yu W Xiong H Yuan F. Circ_0000181 regulates miR-667-5p/NLRC4 axis to promote pyroptosis progression in diabetic nephropathy. Sci Rep. (2022) 12:11994. doi: 10.1038/s41598-022-15607-7, PMID: 35835791 PMC9283475

[68] Zhao L Chen H Zeng Y Yang K Zhang R Li Z. Circular RNA circ_0000712 regulates high glucose-induced apoptosis, inflammation, oxidative stress, and fibrosis in (DN) by targeting the miR-879-5p/SOX6 axis. Endocr J. (2021) 68:1155–64. doi: 10.1507/endocrj.EJ20-0739, PMID: 33980772

[69] Li B Sun G Yu H Meng J Wei F. Circ_0114428 promotes proliferation, fibrosis and EMT process of high glucose-induced glomerular mesangial cells through regulating the miR-185-5p/SMAD3 axis. Autoimmunity. (2022) 55:462–72. doi: 10.1080/08916934.2022.2103797, PMID: 35880624

[70] Anderson AH Xie D Wang X Baudier RL Orlandi P Appel LJ. Novel risk factors for progression of diabetic and nondiabetic CKD: findings from the chronic renal insufficiency cohort (CRIC) study. Am J Kidney Dis. (2021) 77:56–73.e1. doi: 10.1053/j.ajkd.2020.07.011, PMID: 32866540 PMC7752839

[71] Li X Zhang Y Xing X Li M Liu Y Xu A. Podocyte injury of diabetic nephropathy: Novel mechanism discovery and therapeutic prospects. BioMed Pharmacother. (2023) 168:115670. doi: 10.1016/j.biopha.2023.115670, PMID: 37837883

[72] Barutta F Bellini S Gruden G. Mechanisms of podocyte injury and implications for diabetic nephropathy. Clin Sci (Lond). (2022) 136:493–520. doi: 10.1042/CS20210625, PMID: 35415751 PMC9008595

[73] Liu M Liang K Zhen J Zhou M Wang X Wang Z. Sirt6 deficiency exacerbates podocyte injury and proteinuria through targeting Notch signaling. Nat Commun. (2017) 8:413. doi: 10.1038/s41467-017-00498-4, PMID: 28871079 PMC5583183

[74] Wang X Zhao J Li Y Rao J Xu G. Epigenetics and endoplasmic reticulum in podocytopathy during diabetic nephropathy progression. Front Immunol. (2022) 13:1090989. doi: 10.3389/fimmu.2022.1090989, PMID: 36618403 PMC9813850

[75] Fu Y Sun Y Wang M Hou Y Huang W Zhou D. Elevation of JAML promotes diabetic kidney disease by modulating podocyte lipid metabolism. Cell Metab. (2020) 32:1052–1062.e8. doi: 10.1016/j.cmet.2020.10.019, PMID: 33186558

[76] Tsai YC Kuo MC Hung WW Wu PH Chang WA Wu LY. Proximal tubule-derived exosomes contribute to mesangial cell injury in diabetic nephropathy via miR-92a-1-5p transfer. Cell Commun Signal. (2023) 21:10. doi: 10.1186/s12964-022-00997-y, PMID: 36639674 PMC9838003

[77] Dong Z Sun Y Wei G Li S Zhao Z. Ergosterol ameliorates diabetic nephropathy by attenuating mesangial cell proliferation and extracellular matrix deposition via the TGF-β1/smad2 signaling pathway. Nutrients. (2019) 11:483. doi: 10.3390/nu11020483, PMID: 30823598 PMC6412245

[78] Su X Guo H Zhou Y Cao A Shen Q Zhu B. Astragaloside IV attenuates high glucose-induced NF-κB-mediated inflammation through activation of PI3K/AKT-ERK-dependent Nrf2/ARE signaling pathway in glomerular mesangial cells. Phytother Res. (2023) 37:4133–48. doi: 10.1002/ptr.7875, PMID: 37189016

[79] Yao L Liang X Liu Y Li B Hong M Wang X. Non-steroidal mineralocorticoid receptor antagonist finerenone ameliorates mitochondrial dysfunction via PI3K/Akt/eNOS signaling pathway in diabetic tubulopathy. Redox Biol. (2023) 68:102946. doi: 10.1016/j.redox.2023.102946, PMID: 37924663 PMC10661120

[80] Xu W Ye S Liu W Guo H Zhang L Wei S. Single-cell RNA-seq analysis decodes the kidney microenvironment induced by polystyrene microplastics in mice receiving a high-fat diet. J Nanobiotechnol. (2024) 22:13. doi: 10.1186/s12951-023-02266-7, PMID: 38167034 PMC10762848

[81] Lin YC Chang YH Yang SY Wu KD Chu TS. Update of pathophysiology and management of diabetic kidney disease. J Formos Med Assoc. (2018) 117:662–75. doi: 10.1016/j.jfma.2018.02.007, PMID: 29486908

[82] Raval N Kumawat A Kalyane D Kalia K Tekade RK. Understanding molecular upsets in diabetic nephropathy to identify novel targets and treatment opportunities. Drug Discov Today. (2020) 25:862–78. doi: 10.1016/j.drudis.2020.01.008, PMID: 31981791

[83] Guo J Zheng W Liu Y Zhou M Shi Y Lei M. Long non-coding RNA DLX6-AS1 is the key mediator of glomerular podocyte injury and albuminuria in diabetic nephropathy by targeting the miR-346/GSK-3β signaling pathway. Cell Death Dis. (2023) 14:172. doi: 10.1038/s41419-023-05695-2, PMID: 36854759 PMC9975222

[84] Cai Q Wang C Huang L Wu C Yan B Chen T. Long non-coding RNA small nucleolar RNA host gene 5 (SNHG5) regulates renal tubular damage in diabetic nephropathy via targeting miR-26a-5p. Horm Metab Res. (2021) 53:818–24. doi: 10.1055/a-1678-6556, PMID: 34891212

[85] Zhang M Zhao S Xu C Shen Y Huang J Shen S. Ablation of lncRNA MIAT mitigates high glucose-stimulated inflammation and apoptosis of podocyte via miR-130a-3p/TLR4 signaling axis. Biochem Biophys Res Commun. (2020) 533:429–36. doi: 10.1016/j.bbrc.2020.09.034, PMID: 32972755

[86] Jiang Z Qian L Yang R Wu Y Guo Y Chen T. LncRNA TCF7 contributes to high glucose-induced damage in human podocytes by up-regulating SEMA3A via sponging miR-16-5p. J Diabetes Investig. (2023) 14:193–204. doi: 10.1111/jdi.13904, PMID: 36583231 PMC9889678

[87] Xia J Sun W Dun J. LncRNA 1500026H17Rik knockdown ameliorates high glucose-induced mouse podocyte injuries through the miR-205-5p/EGR1 pathway. Int Urol Nephrol. (2023) 55:1045–57. doi: 10.1007/s11255-022-03396-x, PMID: 36306049

[88] Long B Wan Y Zhang S Lv L. LncRNA XIST protects podocyte from high glucose-induced cell injury in diabetic nephropathy by sponging miR-30 and regulating AVEN expression. Arch Physiol Biochem. (2023) 129:610–7. doi: 10.1080/13813455.2020.1854307, PMID: 33332155

[89] Ye L Chen JH Zhu SL Xu DD Yang Y Shi MP. Hsa_circ_0001162 inhibition alleviates high glucose-induced human podocytes injury by the miR-149-5p/MMP9 signaling pathway. Appl Biochem Biotechnol. (2023) 195:7255–76. doi: 10.1007/s12010-023-04431-y, PMID: 36988849

[90] Li Y Wang W Liu N Wang K Ren F. A novel CIRC_SUPT3/MIR-185-5P/G3BP2 cerna network regulates high glucose-induced injury in mouse podocyte MPC5 cells. Shock. (2024) 62:227–34. doi: 10.1097/SHK.0000000000002389, PMID: 38813926

[91] Yao T Zha D Hu C Wu X. Circ_0000285 promotes podocyte injury through sponging miR-654-3p and activating MAPK6 in diabetic nephropathy. Gene. (2020) 747:144661. doi: 10.1016/j.gene.2020.144661, PMID: 32275999

[92] Ma P He Y Wang B Qiu D Xu Q. CircGAB1 facilitates podocyte injury through sponging miR-346 and activating MAPK6 in diabetic nephropathy. Appl Biochem Biotechnol. (2024) 196:1863–75. doi: 10.1007/s12010-023-04645-0, PMID: 37440116

[93] Fang R Cao X Zhu Y Chen Q. Hsa_circ_0037128 aggravates high glucose-induced podocytes injury in diabetic nephropathy through mediating miR-31-5p/KLF9. Autoimmunity. (2022) 55:254–63. doi: 10.1080/08916934.2022.2037128, PMID: 35285770

[94] Chen H Guo Y Cheng X. Long non-cording RNA XIST promoted cell proliferation and suppressed apoptosis by miR-423-5p/HMGA2 axis in diabetic nephropathy. Mol Cell Biochem. (2021) 476:4517–28. doi: 10.1007/s11010-021-04250-x, PMID: 34532814

[95] Wang S Yi P Wang N Song M Li W Zheng Y. LncRNA TUG1/miR-29c-3p/SIRT1 axis regulates endoplasmic reticulum stress-mediated renal epithelial cells injury in diabetic nephropathy model in *vitro* . PloS One. (2021) 16:e0252761. doi: 10.1371/journal.pone.0252761, PMID: 34097717 PMC8183992

[96] Liu C Zhuo H Ye MY Huang GX Fan M Huang XZ. LncRNA MALAT1 promoted high glucose-induced pyroptosis of renal tubular epithelial cell by sponging miR-30c targeting for NLRP3. Kaohsiung J Med Sci. (2020) 36:682–91. doi: 10.1002/kjm2.12226, PMID: 32391974 PMC11896152

[97] Yu R Zhang Y Lu Z Li J Shi P Li J. Long-chain non-coding RNA UCA1 inhibits renal tubular epithelial cell apoptosis by targeting microRNA-206 in diabetic nephropathy. Arch Physiol Biochem. (2022) 128:231–9. doi: 10.1080/13813455.2019.1673431, PMID: 31608712

[98] Liu Q Cui Y Ding N Zhou C. Knockdown of circ_0003928 ameliorates high glucose-induced dysfunction of human tubular epithelial cells through the miR-506-3p/HDAC4 pathway in diabetic nephropathy. Eur J Med Res. (2022) 27:55. doi: 10.1186/s40001-022-00679-y, PMID: 35392987 PMC8991937

[99] Wang H Huang S Hu T Fei S Zhang H. Circ_0000064 promotes high glucose-induced renal tubular epithelial cells injury to facilitate diabetic nephropathy progression through miR-532-3p/ROCK1 axis. BMC Endocr Disord. (2022) 22:67. doi: 10.1186/s12902-022-00968-x, PMID: 35291991 PMC8922934

[100] Rong L Xue H Hao J Liu J Xu H. Long non-coding RNA MEG3 silencing weakens high glucose-induced mesangial cell injury by decreasing LIN28B expression by sponging and sequestering miR-23c. Kidney Res Clin Pract. (2024) 43:600–13. doi: 10.23876/j.krcp.23.090, PMID: 38148128 PMC11467368

[101] Zha F Qu X Tang B Li J Wang Y Zheng P. Long non-coding RNA MEG3 promotes fibrosis and inflammatory response in diabetic nephropathy via miR-181a/Egr-1/TLR4 axis. Aging (Albany NY). (2019) 11:3716–30. doi: 10.18632/aging.102011, PMID: 31195367 PMC6594792

[102] Qin Y Xu Y Peng H Cao M Zhao K Zhu Y. Circ_0123996 promotes the proliferation, inflammation, and fibrosis of mesangial cells by sponging miR-203a-3p to upregulate SOX6 in diabetic nephropathy. J Biochem Mol Toxicol. (2022) 36:e23139. doi: 10.1002/jbt.23139, PMID: 36073553

[103] Fang Z Wang D Sun F Chang J Yuan D Lin S. Circ-Luc7l Absence Attenuates Diabetic Nephropathy Progression by Reducing Mesangial Cell Excessive Proliferation, Inflammation, and Extracellular Matrix Accumulation via Mediating the miR-205-5p/Tgfbr1 Pathway. Biochem Genet. (2024) 62:4896–913. doi: 10.1007/s10528-024-10694-9, PMID: 38376578

[104] Liu J Duan P Xu C Xu D Liu Y Jiang J. CircRNA circ-ITCH improves renal inflammation and fibrosis in streptozotocin-induced diabetic mice by regulating the miR-33a-5p/SIRT6 axis. Inflammation Res. (2021) 70:835–46. doi: 10.1007/s00011-021-01485-8, PMID: 34216220

[105] Sun A Sun N Liang X Hou Z. Circ-FBXW12 aggravates the development of diabetic nephropathy by binding to miR-31-5p to induce LIN28B. Diabetol Metab Syndr. (2021) 13:141. doi: 10.1186/s13098-021-00757-x, PMID: 34863268 PMC8642853

[106] Yun J Ren J Liu Y Dai L Song L Ma X. Circ-ACTR2 aggravates the high glucose-induced cell dysfunction of human renal mesangial cells through mediating the miR-205-5p/HMGA2 axis in diabetic nephropathy. Diabetol Metab Syndr. (2021) 13:72. doi: 10.1186/s13098-021-00692-x, PMID: 34174955 PMC8236153

[107] Shu S Xu Z Lu H Li Z Zhang Y. CircHOMER1 aggravates oxidative stress, inflammation and extracellular matrix deposition in high glucose-induced human mesangial cells. Nephrol (Carlton). (2022) 27:983–93. doi: 10.1111/nep.14115, PMID: 36181383

[108] Zheng H Liu X Song B. Circular RNA circADAM9 Promotes Inflammation, Oxidative Stress, and Fibrosis of Human Mesangial Cells via the Keap1-Nrf2 Pathway in Diabetic Nephropathy. Exp Clin Endocrinol Diabetes. (2023) 131:491–9. doi: 10.1055/a-2105-4921, PMID: 37463596

[109] Chen B Li Y Liu Y Xu Z. circLRP6 regulates high glucose-induced proliferation, oxidative stress, ECM accumulation, and inflammation in mesangial cells. J Cell Physiol. (2019) 234:21249–59. doi: 10.1002/jcp.28730, PMID: 31087368

[110] Calle P Hotter G. Macrophage phenotype and fibrosis in diabetic nephropathy. Int J Mol Sci. (2020) 21:2806. doi: 10.3390/ijms21082806, PMID: 32316547 PMC7215738

[111] Wang X Liu Y Rong J Wang K. LncRNA HCP5 knockdown inhibits high glucose-induced excessive proliferation, fibrosis and inflammation of human glomerular mesangial cells by regulating the miR-93-5p/HMGA2 axis. BMC Endocr Disord. (2021) 21:134. doi: 10.1186/s12902-021-00781-y, PMID: 34187448 PMC8243433

[112] Ge X Xu B Xu W Xia L Xu Z Shen L. Long noncoding RNA GAS5 inhibits cell proliferation and fibrosis in diabetic nephropathy by sponging miR-221 and modulating SIRT1 expression. Aging (Albany NY). (2019) 11:8745–59. doi: 10.18632/aging.102249, PMID: 31631065 PMC6834398

[113] Du Y Feng Y Cai Y Tian C. CircLARP1B promotes pyroptosis of high glucose-induced renal mesangial cells by regulating the miR-578/TLR4 axis. Int Urol Nephrol. (2024) 56:283–93. doi: 10.1007/s11255-023-03672-4, PMID: 37341906

[114] Zhou J Peng X Ru Y Xu J. Correction to: Circ_0060077 Knockdown Alleviates High−Glucose−Induced Cell Apoptosis, Oxidative Stress, Inflammation and Fibrosis in HK−2 Cells via miR−145−5p/VASN Pathway. Inflammation. (2023) 46:780. doi: 10.1007/s10753-022-01740-y, PMID: 36112240

[115] Feng F Yang J Wang G Huang P Li Y Zhou B. Circ_0068087 Promotes High Glucose-Induced Human Renal Tubular Cell Injury through Regulating miR-106a-5p/ROCK2 Pathway. Nephron. (2023) 147:212–22. doi: 10.1159/000525440, PMID: 35871508

[116] Liu SY Wang H Yang B Hou B Sun LS Pang H. CircTAOK1 regulates high glucose induced inflammation, oxidative stress, ECM accumulation, and apoptosis in diabetic nephropathy via targeting miR-142-3p/SOX6 axis. Environ Toxicol. (2024) 39:2197–207. doi: 10.1002/tox.24076, PMID: 38124441

[117] Bao Z Yu X Zhang L. The circ_0003928/miR-31-5p/MAPK6 cascade affects high glucose-induced inflammatory response, fibrosis and oxidative stress in HK-2 cells. Transpl Immunol. (2024) 86:102078. doi: 10.1016/j.trim.2024.102078, PMID: 38964515

[118] An L Ji D Hu W Wang J Jin X Qu Y. Interference of Hsa_circ_0003928 Alleviates High Glucose-Induced Cell Apoptosis and Inflammation in HK-2 Cells via miR-151-3p/Anxa2. Diabetes Metab Syndr Obes. (2020) 13:3157–68. doi: 10.2147/DMSO.S265543, PMID: 32982348 PMC7494388

[119] Zhu Q Zhang C Qu T Lu X He X Li W. MNX1-AS1 promotes phase separation of IGF2BP1 to drive c-myc-mediated cell-cycle progression and proliferation in lung cancer. Cancer Res. (2022) 82:4340–58. doi: 10.1158/0008-5472.CAN-22-1289, PMID: 36214649

[120] Tian F Wang J Zhang Z Yang J. LncRNA SNHG7/miR-34a-5p/SYVN1 axis plays a vital role in proliferation, apoptosis and autophagy in osteoarthritis. Biol Res. (2020) 53:9. doi: 10.1186/s40659-020-00275-6, PMID: 32066502 PMC7027214

[121] Vasudevan SO Behl B Rathinam VA. Pyroptosis-induced inflammation and tissue damage. Semin Immunol. (2023) 69:101781. doi: 10.1016/j.smim.2023.101781, PMID: 37352727 PMC10598759

[122] Coll RC Schroder K Pelegrín P. NLRP3 and pyroptosis blockers for treating inflammatory diseases. Trends Pharmacol Sci. (2022) 43:653–68. doi: 10.1016/j.tips.2022.04.003, PMID: 35513901

[123] Noonin C Thongboonkerd V. Exosome-inflammasome crosstalk and their roles in inflammatory responses. Theranostics. (2021) 11:4436–51. doi: 10.7150/thno.54004, PMID: 33754070 PMC7977448

[124] Yue B Yang H Wang J Ru W Wu J Huang Y. Exosome biogenesis, secretion and function of exosomal miRNAs in skeletal muscle myogenesis. Cell Prolif. (2020) 53:e12857. doi: 10.1111/cpr.12857, PMID: 32578911 PMC7377932

[125] DiStefano JK. The emerging role of long noncoding RNAs in human disease. Methods Mol Biol. (2018) 1706:91–110. doi: 10.1007/978-1-4939-7471-9_6, PMID: 29423795

[126] Merberg D Moreland R Su Z Li B Crooker B Palmieri K. Combined miRNA transcriptome and proteome analysis of extracellular vesicles in urine and blood from the Pompe mouse model. Ann Med. (2024) 56:2402503. doi: 10.1080/07853890.2024.2402503, PMID: 39445404 PMC11504521

[127] He X Kuang G Wu Y Ou C. Emerging roles of exosomal miRNAs in diabetes mellitus. Clin Transl Med. (2021) 11:e468. doi: 10.1002/ctm2.468, PMID: 34185424 PMC8236118

[128] Neal CS Michael MZ Pimlott LK Yong TY Li JY Gleadle JM. Circulating microRNA expression is reduced in chronic kidney disease. Nephrol Dial Transpl. (2011) 26:3794–802. doi: 10.1093/ndt/gfr485, PMID: 21891774

[129] Wang J Tao Y Zhao F Liu T Shen X Zhou L. Expression of urinary exosomal miRNA-615-3p and miRNA-3147 in diabetic kidney disease and their association with inflammation and fibrosis. Ren Fail. (2023) 45:2121929. doi: 10.1080/0886022X.2022.2121929, PMID: 36695327 PMC9879181

[130] Zhang H Jiang Y Song J Wang S Lu J Wei F. Urinary exosomes exacerbate diabetic kidney disease by promoting NLRP3 inflammasome activation via the microRNA-516b-5p/SIRT3/AMPK pathway. Am J Physiol Endocrinol Metab. (2025) 328:E911–23. doi: 10.1152/ajpendo.00527.2024, PMID: 40337989

[131] Gao C Wang B Chen Q Wang M Fei X Zhao N. Serum exosomes from diabetic kidney disease patients promote pyroptosis and oxidative stress through the miR-4449/HIC1 pathway. Nutr Diabetes. (2021) 11:33. doi: 10.1038/s41387-021-00175-y, PMID: 34732690 PMC8566490

[132] Wang L Zhen H Sun Y Rong S Li B Song Z. Plasma exo-miRNAs correlated with AD-related factors of chinese individuals involved in Aβ Accumulation and cognition decline. Mol Neurobiol. (2022) 59:6790–804. doi: 10.1007/s12035-022-03012-0, PMID: 36040555 PMC9425792

[133] Backes C Meese E Keller A. Specific miRNA disease biomarkers in blood, serum and plasma: challenges and prospects. Mol Diagn Ther. (2016) 20:509–18. doi: 10.1007/s40291-016-0221-4, PMID: 27378479

[134] Xu YX Pu SD Li X Yu ZW Zhang YT Tong XW. Exosomal ncRNAs: Novel therapeutic target and biomarker for diabetic complications. Pharmacol Res. (2022) 178:106135. doi: 10.1016/j.phrs.2022.106135, PMID: 35192956

[135] Liu M Zhao J. Circular RNAs in diabetic nephropathy: updates and perspectives. Aging Dis. (2022) 13:1365–80. doi: 10.14336/AD.2022.0203, PMID: 36186139 PMC9466972

[136] Zhu Y Zha F Tang B Ji TT Li XY Feng L. Exosomal hsa_circ_0125310 promotes cell proliferation and fibrosis in diabetic nephropathy via sponging miR-422a and targeting the IGF1R/p38 axis. J Cell Mol Med. (2022) 26:151–62. doi: 10.1111/jcmm.17065, PMID: 34854210 PMC8742240

[137] Li B Sun G Yu H Meng J Wei F. Exosomal circTAOK1 contributes to diabetic kidney disease progression through regulating SMAD3 expression by sponging miR-520h. Int Urol Nephrol. (2022) 54:2343–54. doi: 10.1007/s11255-022-03139-y, PMID: 35142978

[138] Bai S Xiong X Tang B Ji T Li X Qu X. Exosomal circ_DLGAP4 promotes diabetic kidney disease progression by sponging miR-143 and targeting ERBB3/NF-κB/MMP-2 axis. Cell Death Dis. (2020) 11:1008. doi: 10.1038/s41419-020-03169-3, PMID: 33230102 PMC7683700

[139] Niu Z Ren G Huang L Mu L. Circ_0008529 Contributes to Renal Tubular Cell Dysfunction in High Glucose Stress via miR-185-5p/SMAD2 Pathway in Diabetic Nephropathy. Biochem Genet. (2023) 61:963–78. doi: 10.1007/s10528-022-10296-3, PMID: 36316592

[140] Dong Q Dong L Zhu Y Wang X Li Z Zhang L. Circular ribonucleic acid nucleoporin 98 knockdown alleviates high glucose-induced proliferation, fibrosis, inflammation and oxidative stress in human glomerular mesangial cells by regulating the microribonucleic acid-151-3p-high mobility group AT-hook 2 axis. J Diabetes Investig. (2022) 13:1303–15. doi: 10.1111/jdi.13821, PMID: 35482475 PMC9340880

[141] Yamanaka S. Pluripotent stem cell-based cell therapy-promise and challenges. Cell Stem Cell. (2020) 27:523–31. doi: 10.1016/j.stem.2020.09.014, PMID: 33007237

[142] Tan F Li X Wang Z Li J Shahzad K Zheng J. Clinical applications of stem cell-derived exosomes. Signal Transduct Target Ther. (2024) 9:17. doi: 10.1038/s41392-023-01704-0, PMID: 38212307 PMC10784577

[143] Sun Y Zhao Y Lu Y Li H Xiang J Yang D. Urinary stem cell-derived exocrine circRNA ATG7 regulates the SOCS1/STAT3 signaling pathway through miR-4500, inhibits M1 macrophage polarization, and alleviates the progression of diabetes nephropathy. Int Urol Nephrol. (2024) 56:1449–63. doi: 10.1007/s11255-023-03819-3, PMID: 37815664 PMC10924005

[144] Fan MH Zhang XZ Jiang YL Pi JK Zhang JY Zhang YQ. Exosomes from hypoxic urine-derived stem cells facilitate healing of diabetic wound by targeting SERPINE1 through miR-486-5p. Biomaterials. (2025) 314:122893. doi: 10.1016/j.biomaterials.2024.122893, PMID: 39418849

[145] Yong W Ma H Na M Gao T Zhang Y Hao L. Roles of melatonin in the field of reproductive medicine. BioMed Pharmacother. (2021) 144:112001. doi: 10.1016/j.biopha.2021.112001, PMID: 34624677

[146] Zhong L Liao G Wang X Li L Zhang J Chen Y. Mesenchymal stem cells-microvesicle-miR-451a ameliorate early diabetic kidney injury by negative regulation of P15 and P19. Exp Biol Med (Maywood). (2018) 243:1233–42. doi: 10.1177/1535370218819726, PMID: 30614256 PMC6384448

[147] Duan Y Luo Q Wang Y Ma Y Chen F Zhu X. Adipose mesenchymal stem cell-derived extracellular vesicles containing microRNA-26a-5p target TLR4 and protect against diabetic nephropathy. J Biol Chem. (2020) 295:12868–84. doi: 10.1074/jbc.RA120.012522, PMID: 32580945 PMC7489897

[148] Deng P Yuan Q Cheng Y Li J Liu Z Liu Y. Loss of KDM4B exacerbates bone-fat imbalance and mesenchymal stromal cell exhaustion in skeletal aging. Cell Stem Cell. (2021) 28:1057–1073.e7. doi: 10.1016/j.stem.2021.01.010, PMID: 33571444 PMC8178178

[149] Hao Y Miao J Liu W Cai K Huang X Peng L. Mesenchymal stem cell-derived exosomes carry microRNA-125a to protect against diabetic nephropathy by targeting histone deacetylase 1 and downregulating endothelin-1. Diabetes Metab Syndr Obes. (2021) 14:1405–18. doi: 10.2147/DMSO.S286191, PMID: 33790607 PMC8006976

[150] Lv J Hao YN Wang XP Lu WH Xie LY Niu D. Bone marrow mesenchymal stem cell-derived exosomal miR-30e-5p ameliorates high-glucose induced renal proximal tubular cell pyroptosis by inhibiting ELAVL1. Ren Fail. (2023) 45:2177082. doi: 10.1080/0886022X.2023.2177082, PMID: 36794663 PMC9937013

[151] Li R Tao H Pan K Li R Guo Z Chen X. Extracellular vesicles derived from mesenchymal stem cells alleviate renal fibrosis via the miR-99b-5p/mTOR/autophagy axis in diabetic kidney disease. Stem Cell Res Ther. (2025) 16:142. doi: 10.1186/s13287-025-04265-x, PMID: 40103007 PMC11921689

[152] Su W Yin Y Zhao J Hu R Zhang H Hu J. Exosomes derived from umbilical cord-derived mesenchymal stem cells exposed to diabetic microenvironment enhance M2 macrophage polarization and protect against diabetic nephropathy. FASEB J. (2024) 38:e23798. doi: 10.1096/fj.202400359R, PMID: 38989582

[153] Kaze AD Zhuo M Kim SC Patorno E Paik JM. Association of SGLT2 inhibitors with cardiovascular, kidney, and safety outcomes among patients with diabetic kidney disease: a meta-analysis. Cardiovasc Diabetol. (2022) 21:47. doi: 10.1186/s12933-022-01476-x, PMID: 35321742 PMC9491404

[154] Cao H Rao X Jia J Yan T Li D. Identification of tubulointerstitial genes and ceRNA networks involved in diabetic nephropathy via integrated bioinformatics approaches. Hereditas. (2022) 159:36. doi: 10.1186/s41065-022-00249-6, PMID: 36154667 PMC9511769

[155] Sun H Chen T Li X Zhu Y Zhang S He P. The relevance of the non-invasive biomarkers lncRNA GAS5/miR-21 ceRNA regulatory network in the early identification of diabetes and diabetic nephropathy. Diabetol Metab Syndr. (2023) 15:197. doi: 10.1186/s13098-023-01179-7, PMID: 37821982 PMC10566063

[156] Yang H Wang J Zhang Z Peng R Lv D Liu H. Sp1-Induced lncRNA Rmrp Promotes Mesangial Cell Proliferation and Fibrosis in Diabetic Nephropathy by Modulating the miR-1a-3p/JunD Pathway. Front Endocrinol (Lausanne). (2021) 12:690784. doi: 10.3389/fendo.2021.690784, PMID: 34512545 PMC8429906

[157] Jin J Wang Y Zheng D Liang M He Q. A Novel Identified Circular RNA, mmu_mmu_circRNA_0000309, Involves in Germacrone-Mediated Improvement of Diabetic Nephropathy Through Regulating Ferroptosis by Targeting miR-188-3p/GPX4 Signaling Axis. Antioxid Redox Signal. (2022) 36:740–59. doi: 10.1089/ars.2021.0063, PMID: 34913724

[158] Lei X Zhang L Li Z Ren J. Astragaloside IV/lncRNA-TUG1/TRAF5 signaling pathway participates in podocyte apoptosis of diabetic nephropathy rats. Drug Des Devel Ther. (2018) 12:2785–93. doi: 10.2147/DDDT.S166525, PMID: 30233141 PMC6132489

[159] Zhang Y Deng Y Yang Y Yang Z Yin Y Xie J. Polysaccharides from Dendrobium officinale delay diabetic kidney disease interstitial fibrosis through LncRNA XIST/TGF-β1. BioMed Pharmacother. (2024) 175:116636. doi: 10.1016/j.biopha.2024.116636, PMID: 38677245

[160] Sun L Han Y Shen C Luo H Wang Z. Emodin alleviates high glucose-induced oxidative stress, inflammation and extracellular matrix accumulation of mesangial cells by the circ_0000064/miR-30c-5p/Lmp7 axis. J Recept Signal Transduct Res. (2022) 42:302–12. doi: 10.1080/10799893.2021.1933028, PMID: 34151713

[161] Cai S Liu J Ma Q Bao Y Chen J Li Y. Coptis inhibited epithelial-mesenchymal transition and fibrogenesis of diabetic nephropathy through lncRNA CLYBL-AS2-miR-204-5p-SNAI1 axis. J Drug Targeting. (2020) 28:939–48. doi: 10.1080/1061186X.2020.1759077, PMID: 32310009

[162] Jin J Zhang Z Chen J Liu Y Chen Q Wang Q. Jixuepaidu Tang-1 inhibits epithelial-mesenchymal transition and alleviates renal damage in DN mice through suppressing long non-coding RNA LOC498759. Cell Cycle. (2019) 18:3125–36. doi: 10.1080/15384101.2019.1669986, PMID: 31564202 PMC6816411

[163] Lerma E White WB Bakris G. Effectiveness of nonsteroidal mineralocorticoid receptor antagonists in patients with diabetic kidney disease. Postgrad Med. (2023) 135:224–33. doi: 10.1080/00325481.2022.2060598, PMID: 35392754

[164] Bonnesen K Heide-Jørgensen U Christensen DH Christiansen CF Lash TL Hennessy S. Effectiveness of empagliflozin vs dapagliflozin for kidney outcomes in type 2 diabetes. JAMA Intern Med. (2025) 185:314–23. doi: 10.1001/jamainternmed.2024.7381, PMID: 39836391 PMC11877187

[165] Cai Z Liu M Zeng L Zhao K Wang C Sun T. Role of traditional Chinese medicine in ameliorating mitochondrial dysfunction via non-coding RNA signaling: Implication in the treatment of neurodegenerative diseases. Front Pharmacol. (2023) 14:1123188. doi: 10.3389/fphar.2023.1123188, PMID: 36937876 PMC10014574

[166] Li C Meng X Wang L Dai X. Mechanism of action of non-coding RNAs and traditional Chinese medicine in myocardial fibrosis: Focus on the TGF-β/Smad signaling pathway. Front Pharmacol. (2023) 14:1092148. doi: 10.3389/fphar.2023.1092148, PMID: 36843918 PMC9947662

[167] Wu H Tian J Dai D Liao J Wang X Wei X. Efficacy and safety assessment of traditional Chinese medicine for metabolic syndrome. BMJ Open Diabetes Res Care. (2020) 8:e001181. doi: 10.1136/bmjdrc-2020-001181, PMID: 32220922 PMC7170408

[168] Li Y Miao R Liu Y Zhang J Dou Z Zhao L. Efficacy and safety of tripterygium glycoside in the treatment of diabetic nephropathy: A systematic review and meta-analysis based on the duration of medication. Front Endocrinol (Lausanne). (2021) 12:656621. doi: 10.3389/fendo.2021.656621, PMID: 33959100 PMC8095376

[169] Sheng X Dong Y Cheng D Wang N Guo Y. Efficacy and safety of Bailing capsules in the treatment of type 2 diabetic nephropathy: a meta-analysis. Ann Palliat Med. (2020) 9:3885–98. doi: 10.21037/apm-20-1799, PMID: 33222468

[170] Li SH Li L Yang RN Liang SD. Compounds of traditional Chinese medicine and neuropathic pain. Chin J Nat Med. (2020) 18:28–35. doi: 10.1016/S1875-5364(20)30002-9, PMID: 31955821

[171] Li K Xiao K Zhu S Wang Y Wang W. Chinese herbal medicine for primary liver cancer therapy: perspectives and challenges. Front Pharmacol. (2022) 13:889799. doi: 10.3389/fphar.2022.889799, PMID: 35600861 PMC9117702

[172] Sun G Wang J Huang Y Yuan CW Zhang K Hu S. Differences in silencing of mismatched targets by sliced versus diced siRNAs. Nucleic Acids Res. (2018) 46:6806–22. doi: 10.1093/nar/gky287, PMID: 29718312 PMC6061797

[173] Alshaer W Zureigat H Al Karaki A Al-Kadash A Gharaibeh L Hatmal MM. siRNA: Mechanism of action, challenges, and therapeutic approaches. Eur J Pharmacol. (2021) 905:174178. doi: 10.1016/j.ejphar.2021.174178, PMID: 34044011

[174] Ali Zaidi SS Fatima F Ali Zaidi SA Zhou D Deng W Liu S. Engineering siRNA therapeutics: challenges and strategies. J Nanobiotechnol. (2023) 21:381. doi: 10.1186/s12951-023-02147-z, PMID: 37848888 PMC10583313

[175] Gu JF Feng L Zhang MH Qin D Jia XB. New exploration on effect of characteristics of traditional Chinese medicine components structure on multi-ingredient/component pharmacokinetics. Zhongguo Zhong Yao Za Zhi. (2014) 39:2782–6., PMID: 25272515

[176] Peng L Chen Y Shi S Wen H. Stem cell-derived and circulating exosomal microRNAs as new potential tools for diabetic nephropathy management. Stem Cell Res Ther. (2022) 13:25. doi: 10.1186/s13287-021-02696-w, PMID: 35073973 PMC8785577

[177] Hu F Sha W Dai H Yang X Hu P Chu Y. Lower expression of Hsa_circRNA_102682 in diabetic hyperhomocysteinemia negatively related to creatinemia is associated with TGF-β and CTGF. J Clin Lab Anal. (2021) 35:e23860. doi: 10.1002/jcla.23860, PMID: 34296783 PMC8373364

[178] Yang M Song JJ Yang XC Zhong GZ Zhong JC. MiRNA-122-5p inhibitor abolishes angiotensin II-mediated loss of autophagy and promotion of apoptosis in rat cardiofibroblasts by modulation of the apelin-AMPK-mTOR signaling. Vitro Cell Dev Biol Anim. (2022) 58:136–48. doi: 10.1007/s11626-022-00651-4, PMID: 35133561

[179] Fang D Ou X Sun K Zhou X Li Y Shi P. m6A modification-mediated lncRNA TP53TG1 inhibits gastric cancer progression by regulating CIP2A stability. Cancer Sci. (2022) 113:4135–50. doi: 10.1111/cas.15581, PMID: 36114757 PMC9746054

[180] Mao W Wang K Xu B Zhang H Sun S Hu Q. ciRS-7 is a prognostic biomarker and potential gene therapy target for renal cell carcinoma. Mol Cancer. (2021) 20:142. doi: 10.1186/s12943-021-01443-2, PMID: 34740354 PMC8570002

[181] Bosson AD Zamudio JR Sharp PA. Endogenous miRNA and target concentrations determine susceptibility to potential ceRNA competition. Mol Cell. (2014) 56:347–59. doi: 10.1016/j.molcel.2014.09.018, PMID: 25449132 PMC5048918

